# Supplementary material for: The soft explosive model of placental mammal evolution
Source: BMC Evol Biol. 2018 Jul 3;18:104. doi: 10.1186/s12862-018-1218-x (PMC6029115; doi:10.1186/s12862-018-1218-x)
Supplement: Supplementary file 1 — Addressing claims of “zombie” lineages on Phillips’ (2016) timetree. Table S1. and Figure S1. (PDF 588 kb) [file 12862_2018_1218_MOESM1_ESM.pdf]

Additional file 1: Addressing claims of "zombie" lineages on Phillips' (2016) timetree

The soft explosive model of placental mammal evolution

Matthew J. Phillips*[,1] and Carmelo Fruciano[1]

[1]School of Earth, Environmental and Biological Sciences, Queensland University of Technology, Brisbane, Australia

*Corresponding author: E-mail: m9.phillips@qut.edu.au

Contents

Addressing claims of "zombie" lineages on Phillips' (2016) timetree 1

Incorrect or poorly supported fossil placements 1

Figure S1 4

Table S1 6

References 7

Addressing claims of "zombie lineages" on Phillips' (2016) timetree

Phillips [1] found extreme divergence underestimation among large, long-lived taxa that were not calibrated, and argued that calibrating these taxa instead shifted the impact of the underlying rate model misspecification to inflating the ages of nodes deeper in the tree. To avoid this "error-shift inflation", Phillips [1] first inferred divergences with dos Reis et al.'s [2] calibrations, most of which are set among taxa with plesiomorphic life-history (tree 1). Next, the final timetree (tree 2) was inferred with further calibrations added among large, long-lived taxa, but with maximum bounds on several superordinal clades based on broad agreement between tree 1 and fossil records for major diversification following the KPg.

Here we consider Springer et al.'s [3] claims that divergences among small, short-lived taxa in Phillips [1] are underestimated relative to first fossil appearances. Each of these claims is invalid. For 20 of the 22 clades, either (1) the fossil placement is incorrect or poorly supported (see text below) or (2) the molecular and fossil dates are consistent, with the fossil date falling within the 95% CI from Phillips' [1] tree 1 (Table S1). For the remaining two clades, the 95% CIs from Phillips' [1] final molecular dates (tree 2) are entirely older than the proposed fossil minima. The tree 2 analysis was primarily designed to test rate variation hypotheses, and still retains some dubious fossil calibrations from Meredith et al. [2]. But it is notable that our primary (dR40) dating estimates in the present study are also consistent with these proposed fossil minima (Additional file 3). Thus, Springer et al.'s [3] claim of "zombie" lineages among smaller, shorter lived taxa is unfounded.

Incorrect or poorly supported fossil placements

1. Hyracoidea (*Dendrohyrax*, 6.08 Ma): Pickford and Hlusko [4] assigned fragmentary material to *Dendrohyrax*, primarily on the presence of ridges on the base of the mandibular symphysis that are "usually only found in *Dendrohyrax*", although even in *Dendrohyrax* they are often absent. This similarity criterion is not a phylogenetic statement, because no outgroups or other rationale for determining polarity were employed to distinguish the character as synapomorphic for *Dendrohyrax* or primitive for Procaviidae. The authors also note another similarly aged hyrax assigned to *Dendrohyrax*, but again, whether the assignment is based on apomorphic or

plesiomorphic similarity is not tested. Moreover, our tree, and that of Springer et al. [3] does not include *Dendrohyrax*. Springer et al. [3] assume that *Dendrohyrax* is sister to *Procavia*, but this is not the traditional morphological relationship. Even the molecular analysis of Kuntner et al. [5] does not robustly resolve affinities among hyraxes, although weakly favours *Dendrohyrax/Procavia*. If however, the total evidence analysis of Puttick and Thomas [6] is correct, then *Dendrohyrax* falls outside of the hyraxes that are included in the present study (*Heterohyrax* and *Procavia*), delegitimizing the calibration.

2. *Prionodon – Felis* and **3. Felidae – Eupleridae** (*Proailurus*, *Stenogale*, 28. 1 Ma): See Additional file 2: "New calibrations" (3. Feliformia) for discussion on the inappropriateness of *Proailurus* and *Stenogale* as reference taxa for calibrating these clades.

4. Ferae (=Ostentoria, cat – pangolin divergence) (*Ravenictis*, 64 Ma): *Ravenictis* was regarded as a stem carnivoran based on a single upper molar [7]. A further two, rather damaged lower molars from the same Rav W-1 horizon within a quarry in the Ravenscrag Formation were also attributed to an unnamed carnivoran by Fox et al. [8]. Neither of these papers provided any formal phylogenetic analysis (matrix based or otherwise) and instead relied on similarity. Perhaps more importantly, neither paper considered pangolins (the modern sister group of Carnivora). Inferring whether *Ravenictis* and the unnamed lower molar taxon diverged from the carnivoran stem before or after pangolins is not directly possible, because pangolins lack teeth. Even the extinct, probable pangolin relatives, the palaeanodonts have reduced or derived molars, thus clouding their deeper affinities based on teeth. Without being able to confidently place the origin of pangolins within this carnivoramorph framework the Rav W-1 teeth cannot be used to calibrate Ferae. Some authors have also instead placed *Ravenictis* with cimolestids (e.g. [9]).

Another slight variation on this calibration is that Fox et al. [8] acknowledge that "correlation of the Rav W-1 horizon has been contentious", and that it is possibly middle or upper Puercan, such that the appropriate bound would be 63.5 Ma [10].

5. Emballonuroidea and **6. Emballonuroidea – Noctilionoidea** (*Tachypteron*, 47.8 Ma): As discussed in the main text, *Tachypteron* was assigned by Storch et al. [11] only on the basis of similarities, and within a framework that considered emballonurids as sister to rhinolophoids. These two groups are now known to fall on opposite sides of the chiropteran root [12], and some other Eocene European bats previously assigned to Emballonuridae and Rhinolophoidea have

since been placed in a new family of uncertain affinities [13]. *Tachypteron* was not considered in that study. Ravel et al.'s [14] cladistic analysis of *Tachypteron* and *Vespertiliavus* included only emballonuroids among crown bats. Hence, the placement of *Tachypteron* requires confirmation. Noctilionoids were also not considered in these studies.

7. Erinaceidae – Soricidae, **8. Erinaceidae/Soricidae – Talpidae**, and **9. Eulipotyphla** (*Adunator*, 61.6 Ma): Springer et al. [3] cite five articles in support of *Adunator* being an 'erinaceomorph', but all are secondary references (including their own previous molecular dating studies) and rely on the calibration compendia of Benton et al. [15, 16]. Looking within these compendia, two primary research sources are cited, but neither provides a compelling argument for placing *Adunator* within Lipotyphla, let alone as a stem erinaceid. One of these studies, Novacek et al. [17], states that *Adunator* is in "limbo between primitive insectivorans and primitive condylarths", and "we do not exclude the possibility that *Adunator* is a very primitive hyopsodontid". The other study, Smith et al. [18], only sampled *Adunator* alongside the nyctithere, *Leptacodon* (which was assumed to be 'basal') and five species of the putative lipotyphlan fossil genus *Macrocranion* in unrooted maximum parsimony analyses. Hence, Smith et al. [18] could only be used to infer whether the affinities of *Adunator* lie within or outside the sampled genus *Macrocranion*.

Another, more recent study [19], which admittedly also sampled few lipotyphlans, instead places *Adunator* with elephant shrews and several enigmatic Paleogene taxa. A further concern is that these and earlier attempts to infer the relationships of *Adunator* depend primarily on younger taxa, such as the European *Adunator lehmani*. The calibration reference (*Adunator ladae*) has not been tested in formal phylogenetic analyses, which is important, since Secord [20] has placed this older species in a different, resurrected genus (*Mckennatherium*), with even less certain affinities.

As an alternative to *Adunator* we use *Litolestes* as the reference for a 57.8 Ma minimum bound for the erinaceid-soricid clade. This follows Phillips [1], but is tentative, as that paper noted that the erinaceomorph affinities of *Litolestes* require further confirmation. Thus the additional leeway the joint marginal prior allows for this soft (minimum-only) bound node may be appropriate (see Fig. 4). In our primary dR40 MCMCtree analyses, the posterior estimates for the Erinaceidae-Soricidae divergence fall below the specified prior (but within the marginal prior 95% CI). Any potential calibration error here, however, has very little influence on divergence estimation.

Excluding the erinaceid-soricid calibration only shifted the posterior estimates for this clade from 55.38 Ma to 55.32 Ma for the independent rates model and from 54.19 Ma to 54.14 Ma for the autocorrelated rates model. Near-by nodes were similarly little affected.

10. Lorisiformes (*Saharagalago*, 38 Ma): As discussed in the main text, *Saharagalago* is known from just two molars. Phillips ([1], fig. 2) showed this calibration to be an extreme outlier for apparent dating error (or rate distortion). The most likely explanation is that *Saharagalago* (and *Karanisia* from the same locality) falls outside Lorisiformes, as two recent phylogenetic analyses found [21, 22].

11. Lagomorpha (Vastan ankle bones, 53.7 Ma): In the main text we argue that the Vastan ankle bones do not provide a robust reference for the minimum age of crown lagomorphs. Firstly, Rose et al. [23] did not consider sampling error and noted that the morphological signal may be confounded by functional similarities. Secondly, Zhang et al.'s [24] µCT scans show that a key character, the calcaneal canal, is also present in stem lagomorphs. Our reanalysis of Rose et al. [23] excluding the calcaneal canal character places the Vastan ankle bones outside crown lagomorphs, although their affinities remain statistically unresolved (Figure S1).

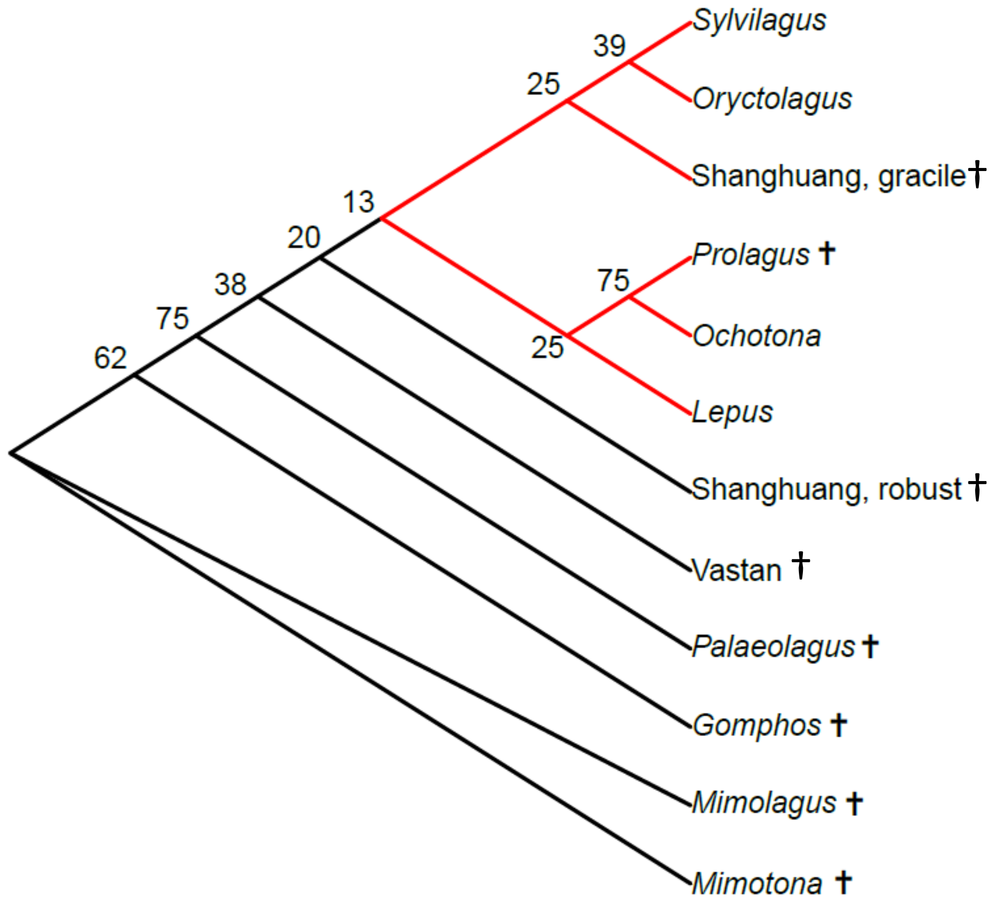

Figure S1: Lagomorph phylogeny from 500 maximum parsimony bootstrap pseudoreplicates, with the crown branches indicated red. The very large number of equally parsimonious trees among Rose et al.'s [23] full (71 taxa) dataset may explain why they did not bootstrap their data. However, bootstrapping becomes tractable when including only the lagomorphs.

12. Aplodontidae – Sciuridae (*Spurimus,* 45.7 Ma): *Spurimus* has been excluded from most phylogenetic analyses. When *Spurimus* was included by Heissig [25] it was placed with primitive ischyromyids that fall far deeper in the rodent phylogeny when datasets have been extended beyond dental characters [26, 27]. Korth's [28] placement of *Spurimus* as an aplodontoid was a "secondary" derivation, based on its potential as an ancestor to *Pelycomys*, and not based on any unambiguous synapomorphies. Hence, *Spurimus* is not acceptable as a reference taxon for strong priors. Even the placement of *Pelecomys* is somewhat ambiguous. Hopkins [29] showed affinities of several putative aplodontoids, including *Pelycomys* lying outside Aplodontoidea, and regardless of the root position, being separated from these by ischyromyids (which may not even be crown rodents). Vianey-Liaud et al. [30] did group *Pelycomys* with aplodontoids, but did not include Springer et al.'s [3] poorly known reference taxon, *Spurimus* or the apparently non-monophyletic ischyromyid taxa that were included by Hopkins [20].

13. Geomyoidea (*Proheteromys*, 31.4 Ma): The heteromyid affinities of *Proheteromys* within Geomyoidea have been questioned by Wahlert [31], who was unable to rule out stem-geomyoid placement. This may in part be explained by Korth and Samuels [32] removing some species described as members of *Proheteromys* and placing them as tentative florentiamyids, which may be stem or crown geomyoids. Korth and Samuels [32] then consider that the earliest *Proheteromys* appearances are Arikareean (Ar1), and at least as old as the Blue Basin Tuff dated 28.8 Ma [33]. However, Korth [34] also places *Proharramys* as a heteromyid, and based on his interpretation of the Cedar Pass Fauna as Whitneyan, this would place a minimum bound on Geomyoidea of 30.6 Ma. However, as for *Proheteromys*, placement of *Proharramys* as a basal heteromyid is based on similarity criteria, and as far as we are aware has not been confirmed with phylogenetic analyses (matrix-based or otherwise). It is premature to calibrate or validate molecular dating with these taxa.

14. Rhinolophidae-Hipposideridae (*Hipposideros*, 38 Ma): Springer et al. [3] cite papers that include bat fossil record compendia as secondary references for the first appearance of *Hipposideros*. But see our discussion on Rhinolophidae-Hipposideridae in Additional file 2:

“New calibrations” (7. Rhinolophidae - Hipposideridae), for problems associated with calibrating this clade.

Table S1. Molecular dates from Phillips’ [1] initial (tree 1) and final (tree 2) timetrees, compared with Springer et al.’s [3] fossil minima for the eight proposed “zombie” lineages among smaller sized, shorter longevity mammal clades that were not included in the above section, *Incorrect or poorly supported fossil placements*. Molecular date 95% CIs are shown in green when they overlap or pre-date proposed fossil minima and in red when they post-date the proposed fossil minima (inducing “zombie” lineages).

Clade	Springer et al.’s [3] fossil minimum (Ma)	Reference fossil	Phillips (2016) Tree 1, 95% CI	Phillips (2016) Tree 2, 95% CI
Tardigrada	15.97	*Imagocnus*	11.7-17.1	15.1-20.6
Xenarthra	47.8	*Riostegotherium*	42.5-51.4	58.0-60.8
Carnivora	38	*Hesperocyon*	34.5-39.1	43.4-47.6
Fereuungulata	64[a]	*Ravenictis*	59.0-64.1	64.2-65.4
Scrotifera	64[a]	*Ravenictis*	59.1-64.2	64.3-65.5
Octodontoidea	24.5[b]	*Sallamys, Xylechimys, Deseadomys*	21.9-24.9	24.4-27.5
Feliformia	28.1[c]	*Proailurus, Stenogale*	22.3-26.1	30.3-35.3
Mustelidae/Ailuridae /Procyonidae	24.8[d]	*Promartes*	17.4-20.5	28.5-30.7

[a] The appropriate date is 63.5 Ma for *Ravenictis*, and the phylogenetic placement of this fossil taxon is highly speculative; see “Incorrect or poorly supported fossil placements” (4. Ferae).

[b]Among two recent papers, Verzi et al. [35] place all three of these reference taxa within crown Octodontoidea, while Arnal and Vucetich [36] place each of these reference taxa outside or in a basal polytomy with crown Octodontoidea. In our bootstrap analyses of these datasets, neither resolves any of the reference taxa as either within or outside the octodontoid crown (not shown).

[c]There is uncertainty over the placements of *Proailurus* and *Stengale*, see Additional file 2: “New Calibrations” (3. Feliformia).

[d]There is uncertainty over the placement of *Promartes*, see Additional file 2: “New Calibrations” (4. Musteloidea).

References

1. Phillips MJ. Geomolecular dating and the origin of placental mammals. Syst Biol. 2016;65(3):546-557.
2. dos Reis M, Inoue J, Hasegawa M, Asher RJ, Donoghue PCJ, Yang Z. Phylogenomic datasets provide both precision and accuracy in estimating the timescale of placental mammal phylogeny. Proc Roy Soc B. 2012;279(1742):3491-3500.
3. Springer MS, Emerling CA, Meredith RW, Janečka JE, Eizirik E, Murphy WJ. Waking the undead: Implications of a soft explosive model for the timing of placental mammal diversification. Mol Phylogenet Evol. 2017;106:86-102.
4. Pickford M, Hlusko LJ. Late Miocene procaviid hyracoids (Hyracoidea, *Dendrohyrax*) from Lemudong'o, Kenya. Kirtlandia. 2007;56:106-111.
5. Kuntner M, May-Collado LJ, Agnarsson I. Phylogeny and conservation priorities of afrotherian mammals (Afrotheria, Mammalia). Zool Scr. 2011;40(1):1-15.
6. Puttick MN, Thomas GH. Fossils and living taxa agree on patterns of body mass evolution: a case study with Afrotheria. Proc Roy Soc B. 2015;282(1821):20152023.
7. Fox RC, Youzwyshyn GP. New primitive carnivorans (Mammalia) from the Paleocene of western Canada, and their bearing on relationships of the order. J Vertebr Paleontol. 1994;14(3):382-404.
8. Fox RC, Scott CS, Rankin BD. New Early Carnivoran Specimens from the Puercan (Earliest Paleocene) of Saskatchewan, Canada. J Paleontol. 2010;84(6):1035-1039.
9. McKenna MC, Bell SK. Classification of mammals above the species level. New York: Columbia University Press. 1997.
10. Ogg JG. Geomagnetic polarity time scale. In Gradstein, FM., Ogg JG, Schmitz MD, Ogg G, editors. The Geologic Time Scale 2012; 2012. Oxford: Elsevier; p. 85-113.
11. Storch G, Sigé B, Habersetzer J. *Tachypteron franzeni* n. gen., n. sp., earliest emballonurid bat from the Middle Eocene of Messel (Mammalia, Chiroptera). Paläontologische Zeitschrift. 2002;76(2):189-199.
12. Teeling EC, Springer MS, Madsen O, Bates P, Brien SJ, Murphy WJ. A molecular phylogeny for bats illuminates biogeography and the fossil record. Science. 2005;307:580-584.
13. Maitre E, Sigé B, Escarguel G. A new family of bats in the Paleogene of Europe: systematics and implications for the origin of emballonurids and rhinolophoids. Neues Jahrbuch fur Geologie und Palaontologie-Abhandlungen. 2008;250(2):199-216.

14. Ravel A, Adaci M, Bensalah M, Charruault A-L, Essid EM, Ammar HK, Marzougui W, Mahboubi M, Mebrouk F, Merzeraud G et al. Origine et radiation initiale des chauves-souris modernes : nouvelles découvertes dans l'Éocène d'Afrique du Nord. Geodiversitas. 2016;38(3):355-434.
15. Benton MJ, Donoghue PC, Asher RJ, Friedman M, Near TJ, Vinther J. Constraints on the timescale of animal evolutionary history. Palaeontol Electronica. 2015;18(1):1-106.
16. Benton MJ, Donoghue PCJ, Asher RJ. Calibrating and constraining molecular clocks. In: Hedges B, Kumar S, editors. The timetree of life. Oxford: Oxford University Press. 2009. p. 35-86.
17. Novacek MJ, Bown TM, Schankler DM. On the classification of early Tertiary Erinaceomorpha (Insectivora, Mammalia). Am Mus Novit. 1985;2813:1-22.
18. Smith T, Bloch JI, Strait SG, Gingerich PD. New species of *Macrocranion* (Mammalia, Lipotyphla) from the earliest Eocene of North America and its biogeographic implications. Contrib Mus Paleontol Univ Michigan. 2002;30:373-384.
19. Hooker JJ, Russell DE. Early Palaeogene Louisinidae (Macroscelidea, Mammalia), their relationships and north European diversity. Zool J Linn Soc. 2012;164(4):856-936.
20. Secord R. The Tiffanian Land-Mammal Age (Middle and Late Paleocene) in the northern Bighorn Basin, Wyoming. University of Michigan Papers on Paleontology 2008;35:1–192.
21. Marivaux L, Ramdarshan A, Essid EM, Marzougui W, Ammar HK, Lebrun R, Marandat B, Merzeraud G, Tabuce R, Vianey-Liaud M. *Djebelemur*, a tiny pre-tooth-combed primate from the Eocene of Tunisia: a glimpse into the origin of crown strepsirhines. PLoS One. 2013;8(12):e80778.
22. Herrera JP, Dávalos LM. Phylogeny and divergence times of lemurs inferred with recent and ancient fossils in the tree. Syst Biol. 2016;65(5):772-791.
23. Rose KD, DeLeon VB, Missiaen P, Rana RS, Sahni A, Singh L, Smith T. Early Eocene lagomorph (Mammalia) from Western India and the early diversification of Lagomorpha. Proc Roy Soc B. 2008;275(1639):1203-1208.
24. Zhang Z, Li C, Wang J. Presence of the calcaneal canal in basal Glires. Vertebrata PalAsiatica. 2016;54(3):235-242.
25. Heissig K. Origin and early dispersal of the squirrels and their relatives. Deinsea. 2003;10(17):277-286.
26. Asher RJ, Meng J, Wible JR, McKenna MC, Rougier GW, Dashzeveg D, Novacek MJ. Stem Lagomorpha and the antiquity of Glires. Science. 2005;307(5712):1091-1094.

27. Wible JR, Wang Y, Li C, Dawson MR. Cranial anatomy and relationships of a new ctenodactyloid (Mammalia, Rodentia) from the early Eocene of Hubei Province, China. Ann Carnegie Mus 2005;74(2):91-150.
28. Korth WW. Aplodontid Rodents of the genus *Pelycomys* Galbreath from the Orellan (Middle Oligocene) of Nebraska. J Mammal. 1986;67(3):545-550.
29. Hopkins SSB. Phylogeny and evolutionary history of the Aplodontoidea (Mammalia: Rodentia). Zool J Linn Soc. 2008;153(4):769-838.
30. Vianey-Liaud M, Gomes Rodrigues H, Marivaux L. Early adaptive radiations of Aplodontoidea (Rodentia, Mammalia) on the Holarctic region: systematics, and phylogenetic and paleobiogeographic implications. Paläontologische Zeitschrift. 2013;87(1):83-120.
31. Wahlert J. The fossil record. In: Genoways HH, Brown JH, editors. Biology of the Heteromyidae. Special Publication, American Society of Mammalogists. 1993;10:1-37.
32. Korth WW, Samuels JX. New Rodent material from the John Day Formation (Arikareean, Middle Oligocene to Early Miocene) of Oregon. Ann Carnegie Mus 2015;83(1):19-84.
33. Albright LB, Woodburne MO, Fremd TJ, Swisher CC, MacFadden BJ, Scott GR. Revised chronostratigraphy and biostratigraphy of the John Day Formation (Turtle Cove and Kimberly Members), Oregon, with implications for updated calibration of the Arikareean North American Land Mammal Age. J Geol. 2008;116(3):211-237.
34. Korth WW. Rodents (Mammalia) from the Whitneyan (Middle Oligocene) Cedar Pass Fauna of South Dakota. Ann Carnegie Mus. 2014;82(4):373-398.
35. Verzi DH, Olivares AI, Morgan CC. Phylogeny and evolutionary patterns of South American octodontoid rodents. Acta Palaeontol Pol. 2014;59(4):757-769.
36. Arnal M, Vucetich MG. Main radiation events in Pan-Octodontoidea (Rodentia, Caviomorpha). Zool J Linn Soc. 2015;175(3):587-606.
