# Supplementary material for: The soft explosive model of placental mammal evolution
Source: BMC Evol Biol. 2018 Jul 3;18:104. doi: 10.1186/s12862-018-1218-x (PMC6029115; doi:10.1186/s12862-018-1218-x)
Supplement: Supplementary file 2 — MCMCtree timetrees. Table S2. Figure S2. and Figure S3. (PDF 525 kb) [file 12862_2018_1218_MOESM2_ESM.pdf]

Additional File 2: MCMCtree timetrees

The soft explosive model of placental mammal evolution

Matthew J. Phillips*[,1] and Carmelo Fruciano[1]

[1]School of Earth, Environmental and Biological Sciences, Queensland University of Technology, Brisbane, Australia

*Corresponding author: E-mail: m9.phillips@qut.edu.au

Contents

Calculation of the midpoint of the placental mammal diversification......................................1

Table S2...2

Rate change across nodes ……………………………………………………………...…3

Figure S2 ……………………………………………………………………………..…..5

Joint marginal priors for the primary dR40 analyses …………………………………....…6

Figure S3 6

MCMCtree timetrees 11

122-taxon dR32 trees 111

122-taxon dR40 trees 113

dR40 trees with key calibration bounds from Springer et al. (2017) 16

dR40 trees with key maximum bounds from Springer et al. (2017) ………….……......18

128-taxon trees...21

57-taxon trees...23

References...25

Calculation of the midpoint of the placental mammal diversification

Most recent molecular dating studies (e.g. [1-4]) acknowledge that there is close temporal correspondence between the initial diversifications within Laurasiatheria, Euarchontoglires, and Afrotheria, while the divergences between these superorders occurred somewhat earlier, and the paenungulate crown originated later. To standardize the variation in timing of the primary interordinal diversification of placental mammals across studies we calculated the diversification midpoint as the median of the relevant interordinal nodes. Hence, our median is calculated from among twelve node ages: the five Laurasiatheria nodes among Lipotyphla, Chiroptera, Artiodactyla, Perissodactyla, Carnivora, and Pholidota, the four Euarchontoglires nodes among Primates, Dermoptera, Scandentia, Rodentia, and Lagomorpha, and the three Afrotheria nodes among Paenungulata, Macroscelidea, Afrosoricida, and Tubulidentata. Relationships within the superorders vary slightly across studies, and so we do not assume a particular phylogeny, but regard the median as falling between the 6th and 7th oldest of the twelve nodes across those three superorders.

In some cases not all nodes are present, but the appropriate midpoint for comparison can still be deduced. One example is our 122-taxon dR32 analysis, which is missing Perissodactyla (therefore, the Zooamata node) and Tubulidentata (therefore, the Afroinsectiphilia node). Among the ten available nodes, we can see that the parent nodes of Zooamata and Afroinsectiphilia are already younger than the 6th and 7th oldest nodes and therefore, the missing nodes must be younger still. Hence, we can still calculate the median between the 6th and 7th oldest of the twelve relevant nodes.

In rare cases the 6th or 7th oldest node was missing due to the exclusion of large, long-lived taxa. This could be interpolated with reference to the result from the full dataset. For example, Zooamata would be the 7th oldest node for the 57-taxon, autocorrelated timetree. The clade age was interpolated between the estimated dates for Scrotifera and Ferae, given the timing of Zooamata at 74% of the age difference between these older and younger nodes for the full dataset autocorrelated analysis.

Where studies presented more than one set of dates, we focused on those that were presented in the main figure or table. When no single tree was clearly preferred we averaged the median over multiple timetrees (e.g. the four combinations of model and bound type for [2]). For Halliday et al. [5], divergence times were obtained based on all the trees they obtained across multiple analyses using different constraints (T.J. Halliday, *pers. comm.*). Each tree was first pruned to only the species these authors assigned to an order. Then, for each of our focal nodes described above, we retained only those trees in which the node was monophyletic, and computed the median time across all the retained trees.

Table S2. Placental interordinal diversification midpoints. Timetrees are classified into models of placental interordinal diversification: short-fuse has interordinal and most ordinal crown divergences in the Cretaceous, long-fuse has Cretaceous interordinal divergences and mostly Cenozoic ordinal crown divergences, hard explosive has all interordinal divergences close to or following the 66 Ma KPg boundary, soft explosive has most interordinal divergences close to or following the 66 Ma KPg boundary, but allows for earlier divergences between the superorders (Laurasiatheria, Euarchontoglires, Afrotheria, and Xenarthra). Note that some timetrees have elements of two models.

Study	Midpoint (Ma)	Placental diversification model & (method)
Bininda-Emonds et al. [6][a]	88.4	Short-fuse (molecular dating)
Murphy et al. [7]	84.4	Short/long-fuse (molecular dating)
Springer et al. [8]	82.0	Short/long-fuse (molecular dating)
Meredith et al. [3]	81.4	Short/long-fuse (molecular dating)
Timetree.org (30/01/2017)	78.5	Short/long-fuse (molecular dating)
Lartillot et al. [9] [b]	75.0	Long-fuse (molecular dating)
Springer et al. [2]	74.1	Long-fuse (molecular dating)
Tarver et al. [10]	72.0	Long-fuse (molecular dating)
dos Reis et al. [4]	71.0	Long-fuse (molecular dating)
Liu et al. [11][c]	68.0	Long-fuse/soft explosive (molecular dating)
Ronquist et al. [12][d]	67.0	Long-fuse/soft explosive (Total evidence)
Halliday et al. [5]	65.1	Hard explosive (Fossil tip dating)
Phillips [1][e]	64.6	Soft explosive (molecular dating)
**This study (122-taxon dR40)**[f]	**64.5**	Soft explosive (molecular dating)
**This study (57-taxon)**[c]	**63.2**	Soft explosive (molecular dating)

[a]Based on the corrected dates associated with the published corrigendum
[b]Mixed-clock (independent/autocorrelated), tip dating, DNA matrix only

[c]Liu et al.'s [11] favoured genewise-partitioned, independent rates analyses, averaged over their three "1st quintile" 200 gene alignments.

[d]Total evidence dating, rapid diversification model

[e]dR27 analysis, with independent rates, soft calibration bounds – the preferred tree for unconstrained interordinal divergences.

[f]Average of the independent and autocorrelated rates analyses.

Rate change across nodes

Fossil record and phylogenetic arguments against a calibration can be supplemented by identification of anomalous rate shifts induced by the calibration, as we have previously argued for monotremes [13]. Here we follow Barba-Montoya et al. [14] in plotting rate changes across nodes for the placental mammals in the 122-taxon dataset on our primary dR40 timetree and for the $dR40_{Springer}$ analysis, which substitutes in key lagomorph, lorisiform, erinaceid-soricid, emballonuroid, and basal rodent, primate and chiropteran calibrations employed by Springer et al. [2]. Here we focus on the independent rates timetrees, because autocorrelated rates are less free to vary between adjacent branches.

To allow several comparisons with the analyses of Meredith et al. [15] and Springer et al. [2], for which branch-times are given, but not rates, we first estimated DNA branch-lengths from the 26-gene DNA alignment from [15], which is also the base alignment employed in [2] and for our primary analyses. The same 26 gene partitions and GTR+Γ models employed for our MCMCtree analyses were used to estimate the DNA branch-lengths in IQ-Tree [16]. The rate of DNA evolution was then given by dividing DNA branch-lengths by branch times. Figure S2 shows relative rate changes across nodes. A daughter lineage rate twice the parent rate will give a unit-less rate acceleration of 2.0, and we use the negative inverse to give comparable decelerations, such that a daughter lineage rate that is half the parent rate gives a rate deceleration of -2.0. No rate change across branches gives 1.0. Each key node is represented by two dots, one for the rate change from the stem to each daughter lineage.

For our primary dR40 timetree, most of the rate changes across the key calibrated nodes (indicated by coloured dots in Figure S2A) fall within the 95% CI (-1.9 – 1.75), with only the chiropteran stem to Yangochiroptera rate acceleration (1.80) slightly above. The 95% CI

widens (-2.11 – 1.81) for the dR40$_{Springer}$ analysis with key nodes calibrated following Springer et al. [2], but now the lagomorph stem to hare (-2.38) and both lorisiform lineages (-2.14, -2.15) fall lower than the 95% CI. The mean lagomorph divergence is close to that clade's calibration minimum bound. However, the mean age estimate for Lorisiformes when employing the key calibrations from Springer et al. [2] is 23.8 Ma, only 63% of their minimum age bound. As such, using soft bounds underestimates the rate change that would be induced by the actual fossil minimum. The orange arrows in Figure S2 indicate extreme rate shifts (4.69-fold deceleration) across Lorisiformes for both daughter lineages if instead we use the relevant branch times for the parent and daughter lineages from the hard-bound, independent rates analysis of Springer et al. [2].

The next greatest rate change in the 122-taxon dataset is across Hystricognathi, to the porcupine *Hystrix*. However, this shift is largely independent of the calibration regime, being -2.86 for our preferred dR40 analysis, and -2.77 for dR40$_{Springer}$, with the key calibrations from [2]. Substantial deceleration may be consistent with a life history rate correlate shift along the *Hystrix* lineage to larger body mass and longer generation times. If we consider the dates from Meredith et al. [15], the large rate decelerations across nodes are associated with increasing body size and generation time, such as across Sirenia (-3.22 to manatees) and Cetacea (-2.86 to baleen whales). It is important to note here that the "rate-shift" inflation that relates to undercompensating for parallel rate shifts in the simulated data (Fig. 2), and that we propose can partly explain the interordinal divergence overestimation on the empirical data, is underpinned by several consecutive rate decelerations, not just rate changes across individual nodes.

No substantial life history shifts are apparent among Lorisiformes. The extreme rate deceleration across Lorisifomes is likely to be an artefact of calibration error, given that the *Saharagalago* reference fossil is known from only two molars and recent studies have placed the taxon outside Lorisiformes (see Additional file 1: "Incorrect or poorly supported fossil placements": 10. Lorisiformes).

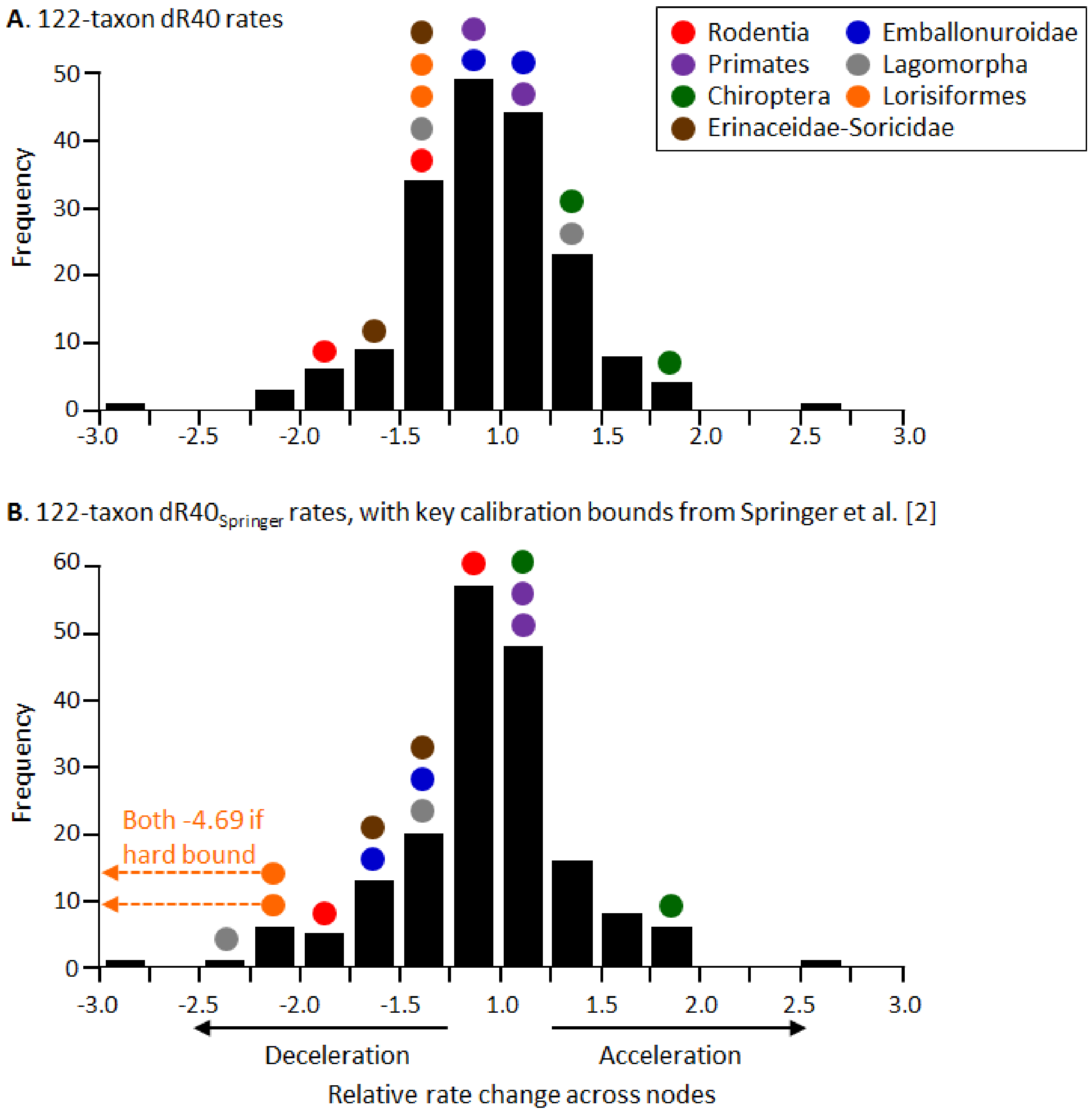

Figure S2. Histogram for relative rate changes across all placental mammal nodes in the 122-taxon, independent rates MCMCtree timetrees for (A) our primary dR40 analysis and (B) the $dR40_{Springer}$ analysis with key calibration bounds substituted from Springer et al. [2]. The coloured dots indicate where the key calibrated nodes fit on the histograms. Each node is represented by two dots, one for the rate change from the stem to each daughter lineage. In most cases the mean divergence estimates are within the soft calibration bounds (or fall less than 10% from the relevant bound. The orange arrows indicate more extreme rate deceleration (4.69-fold) across Lorisiformes for both daughter lineages if we use the relevant branch times for the parent and daughter lineages from the hard-bound, independent rates analysis of Springer et al. [2].

Joint marginal priors for the primary dR40 analyses

The dR40 posterior estimates for the placental crown divergence fall somewhat younger than the joint marginal prior, and so do not appear to be driven by the prior, which the data may instead be "pulling" against. A caveat here, is that the influence of the marginal priors might not be directly translatable upon the inclusion of data, which alters the context of branch rates and times.

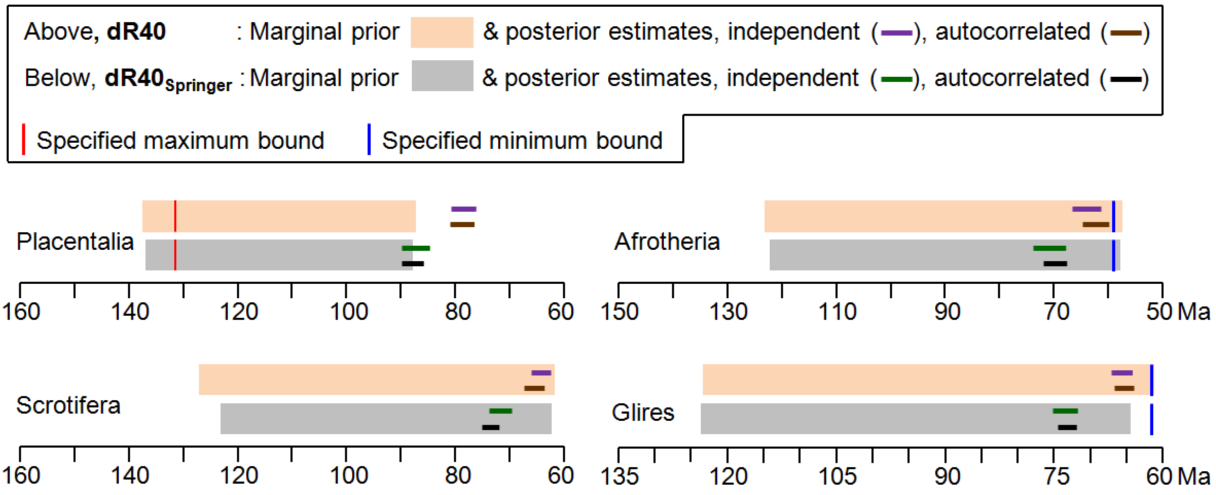

Figure S3. Comparison of the dR40 and dR40$_{\text{Springer}}$ calibration bounds, joint marginal priors, and posterior divergence estimates for Placentalia and the interordinal divergences closest to the midpoint of the primary placental diversification within the superorders Afrotheria, Laurasiatheria and Euarchontoglires. For each clade the calibration bounds and 95% CIs for marginal priors and posterior estimates are shown (above) for our preferred dR40 calibration set, and (below) for the dR40$_{\text{Springer}}$ calibration set with calibration bounds substituted in from Springer et al. [2]. Posterior estimates are shown separately for the autocorrelated and independent rates models, however, the marginal priors under these two rates models are effectively the same, and here for clarity we average over the slight, primarily stochastic differences between them (marginal priors and posterior estimates are provided below).

Joint marginal prior for the 122-taxon dR40 trees

A. Autocorrelated rates

(((((((((Didelphis: 0.194889, Monodelphis: 0.194889) '0.116-0.285': 0.145488, Glironia: 0.340377) '0.165-0.558': 0.126925, Caluromys: 0.467302) '0.256-0.656': 0.224320, (Dromiciops: 0.610776, ((Notoryctes: 0.466701, (((Isoodon: 0.128085, Echymipera: 0.128085) '0.043-0.235': 0.137236, Macrotis: 0.265321) '0.091-0.466': 0.124696, (Dasyurus: 0.271840, Myrmecobius: 0.271840) '0.154-0.466': 0.118177) '0.244-0.558': 0.076684) '0.303-0.612': 0.096490, ((((Acrobates: 0.387730, ((Petaurus: 0.298896, Pseudochirops: 0.298896) '0.244-0.399':

0.046582, Tarsipes: 0.345478) '0.261-0.487': 0.042251) '0.283-0.542': 0.049536, ((Macropus: 0.263608, Aepyprymnus: 0.263608) '0.170-0.416': 0.093357, Hypsiprymnodon: 0.356965) '0.252-0.506': 0.080301) '0.319-0.567': 0.039365, (Cercartetus: 0.355056, Trichosurus: 0.355056) '0.249-0.517': 0.121575) '0.351-0.589': 0.039425, Phascolarctos: 0.516055) '0.372-0.667': 0.047136) '0.414-0.726': 0.047586) '0.475-0.783': 0.080846) '0.558-0.820': 0.080577, (Caenolestes: 0.082726, Rhyncholestes: 0.082726) '0.006-0.160': 0.689474) '0.624-0.847': 0.717119, (((((Echinops: 0.398610, (Amblysomus: 0.256624, Chrysochloris: 0.256624) '0.012-0.502': 0.141987) '0.163-0.632': 0.158449, (Elephantulus: 0.329898, Rhynchocyon: 0.329898) '0.032-0.589': 0.227161) '0.331-0.930': 0.287126, (Heterohyrax: 0.386945, Procavia: 0.386945) '0.042-0.765': 0.457240) '0.571-1.227': 0.188488, (Dasypus: 0.721446, ((Choloepus: 0.271424, Bradypus: 0.271424) '0.159-0.403': 0.164882, Cyclopes: 0.436306) '0.237-0.782': 0.285140) '0.462-1.193': 0.311227) '0.663-1.319': 0.158226, ((((Tragulus: 0.773695, (((((Prionodon: 0.230238, Felis: 0.230238) '0.016-0.385': 0.105929, ((Fossa: 0.179961, Suricata: 0.179961) '0.006-0.360': 0.095794, Genetta: 0.275755) '0.075-0.391': 0.060411) '0.209-0.407': 0.037949, Nandinia: 0.374115) '0.294-0.416': 0.101253, ((Ailurus: 0.230156, Procyon: 0.230156) '0.011-0.388': 0.115721, Mephitis: 0.345877) '0.252-0.414': 0.129490) '0.397-0.605': 0.103631, (Manis_pentadactyla: 0.339377, Manis_tricuspis: 0.339377) '0.032-0.578': 0.239621) '0.433-0.856': 0.194697) '0.576-1.169': 0.120774, (((((Craseonycteris: 0.235424, Megaderma: 0.235424) '0.010-0.435': 0.120199, Rhinopoma: 0.355623) '0.145-0.524': 0.100426, (Hipposideros: 0.392005, Rhinolophus: 0.392005) '0.334-0.509': 0.064044) '0.352-0.571': 0.048725, Nyctimene: 0.504774) '0.379-0.587': 0.056596, (((((Miniopterus: 0.198784, Myotis: 0.198784) '0.007-0.396': 0.101850, Tadarida: 0.300634) '0.077-0.491': 0.074029, Natalus: 0.374663) '0.184-0.548': 0.071998, Myzopoda: 0.446661) '0.289-0.574': 0.078415, ((Emballonuridae: 0.306506, Nycteris: 0.306506) '0.025-0.528': 0.167972, ((((Furipterus: 0.211154, Noctilio: 0.211154) '0.010-0.398': 0.106233, (Pteronotus: 0.211512, Artibeus: 0.211512) '0.007-0.394': 0.105875) '0.125-0.475': 0.051892, Thyroptera: 0.369279) '0.212-0.525': 0.050318, Mystacina: 0.419598) '0.277-0.558': 0.054880) '0.345-0.578': 0.050598) '0.404-0.590': 0.036294) '0.474-0.598': 0.333100) '0.619-1.267': 0.130387, ((((Erinaceus: 0.346495, Podogymnura: 0.346495) '0.033-0.588': 0.282273, Sorex: 0.628768) '0.487-0.992': 0.087931, Talpa: 0.716699) '0.520-1.168': 0.125196, Solenodon: 0.841895) '0.554-1.261': 0.182961) '0.709-1.313': 0.101126, ((((Cynocephalus: 0.368651, Galeopterus: 0.368651) '0.043-0.657': 0.326245, ((Callithrix: 0.439631, Tarsius: 0.439631) '0.338-0.561': 0.141795, ((((Microcebus: 0.209353, Propithecus: 0.209353) '0.008-0.401': 0.108204, Lemur: 0.317558) '0.104-0.490': 0.081358, Daubentonia: 0.398915) '0.241-0.536': 0.089134, (Otolemur: 0.307580, Nycticebus: 0.307580) '0.019-0.514': 0.180469) '0.384-0.560': 0.093376) '0.559-0.615': 0.113470) '0.566-1.027': 0.194575, (Ptilocercus: 0.391142, Tupaia: 0.391142) '0.045-0.796': 0.498329) '0.620-1.258': 0.148168, ((Leporidae: 0.384727, Ochotona: 0.384727) '0.036-0.750': 0.458180, ((((((((Abrocoma: 0.363795, ((Capromys: 0.265517, (Myocastor: 0.174827, Hoplomys: 0.174827) '0.005-0.361': 0.090690) '0.059-0.400': 0.061907, (Ctenomys: 0.221356, Octodontomys: 0.221356) '0.010-0.387': 0.106067) '0.174-0.438': 0.036372) '0.248-0.478': 0.040663, (Chinchilla: 0.268453, Dinomys: 0.268453) '0.020-0.438': 0.136005) '0.302-0.512': 0.038803, ((Agouti: 0.301483, (Cavia: 0.197764, Dasyprocta: 0.197764) '0.006-0.384': 0.103719) '0.096-0.443': 0.071771, Erethizon: 0.373254) '0.217-0.505': 0.070008) '0.345-0.544': 0.032900, (Heterocephalus: 0.364748, (Petromus: 0.240631, Thryonomys: 0.240631) '0.010-0.430': 0.124117) '0.154-0.516': 0.111414) '0.375-0.563': 0.032049, Hystrix: 0.508210) '0.405-0.577': 0.026788, (Ctenodactylus: 0.329753, Laonastes: 0.329753) '0.026-0.551': 0.205245) '0.448-0.586': 0.029698, (((Anomalurus: 0.294528, Pedetes: 0.294528) '0.019-0.490': 0.158185, (((((Cricetus: 0.162654, Mus: 0.162654) '0.004-0.358': 0.087174, Petromyscus: 0.249827) '0.048-0.400': 0.059176, Calomyscidae: 0.309004) '0.134-0.453': 0.048937, Spalax: 0.357941) '0.197-0.495': 0.045700, Jaculus: 0.403640) '0.274-0.527': 0.049073) '0.336-0.550': 0.052901, (Geomyidae: 0.322115, Dipodomys: 0.322115) '0.034-0.525': 0.183498) '0.412-0.562': 0.059083) '0.527-0.592': 0.028655, ((Aplodontia: 0.274080, Sciuridae: 0.274080) '0.012-0.526': 0.156461, Gliridae: 0.430541) '0.204-0.590': 0.162810) '0.563-0.616': 0.249556) '0.614-1.235': 0.194732) '0.722-1.310': 0.088342) '0.797-1.346': 0.064917) '0.886-1.379': 0.298419) '1.250-1.710': 0.372598, (Ornithorhynchus: 0.461239, Tachyglossus: 0.461239) '0.059-1.255': 1.400678) '1.631-2.083': 1.423082, ((Gallus: 0.764450, Taeniopygia: 0.764450) '0.660-0.865': 2.013830, Anolis: 2.778280) '2.560-2.999': 0.506719) '3.121-3.470': 0.272945, Xenopus: 3.557945) '3.313-3.773': 0.649247, Danio: 4.207192) '4.160-4.254';

B. Independent rates

(((((((((Didelphis: 0.195709, Monodelphis: 0.195709) '0.116-0.283': 0.145881, Glironia: 0.341590) '0.158-0.556': 0.126816, Caluromys: 0.468406) '0.244-0.656': 0.224905, (Dromiciops: 0.614436, ((Notoryctes: 0.469418, (((Isoodon: 0.127525, Echymipera: 0.127525) '0.042-0.234': 0.137490, Macrotis: 0.265015) '0.092-0.457': 0.127970, (Dasyurus: 0.276066, Myrmecobius: 0.276066) '0.155-0.460': 0.116919) '0.247-0.558': 0.076434) '0.310-0.611': 0.096835, ((((Acrobates: 0.391058, ((Petaurus: 0.298746, Pseudochirops: 0.298746) '0.243-0.400': 0.047844, Tarsipes: 0.346590) '0.260-0.474': 0.044468) '0.283-0.527': 0.050385, ((Macropus: 0.265970, Aepyprymnus: 0.265970) '0.170-0.410': 0.095109, Hypsiprymnodon: 0.361079) '0.253-0.505': 0.080364) '0.327-0.568': 0.039431, (Cercartetus: 0.356328, Trichosurus: 0.356328) '0.247-0.514': 0.124546) '0.355-0.594': 0.038585, Phascolarctos: 0.519460) '0.383-0.666': 0.046794) '0.419-0.739': 0.048182) '0.468-0.781': 0.078875) '0.560-0.819': 0.078666, (Caenolestes: 0.078556, Rhyncholestes: 0.078556) '0.004-0.159': 0.693420) '0.626-0.848': 0.714039, (((((Echinops: 0.401019, (Amblysomus: 0.255299, Chrysochloris: 0.255299) '0.007-0.503': 0.145720) '0.156-0.625': 0.157656, (Elephantulus: 0.330185, Rhynchocyon: 0.330185) '0.024-0.589': 0.228489) '0.328-0.962': 0.279761, (Heterohyrax: 0.385467, Procavia: 0.385467) '0.029-0.761': 0.452969) '0.572-1.235': 0.182535, (Dasypus: 0.713811, ((Choloepus: 0.271670, Bradypus: 0.271670) '0.159-0.406': 0.163145, Cyclopes: 0.434815) '0.240-0.739': 0.278996) '0.464-1.184': 0.307160) '0.659-1.321': 0.156778, ((((Tragulus: 0.765036, (((((Prionodon: 0.223368, Felis: 0.223368) '0.007-0.385': 0.110435, ((Fossa: 0.175101, Suricata: 0.175101) '0.003-0.354': 0.095926, Genetta: 0.271027) '0.055-0.392': 0.062775) '0.202-0.407': 0.039805, Nandinia: 0.373607) '0.293-0.416': 0.099843, ((Ailurus: 0.223730, Procyon: 0.223730) '0.006-0.388': 0.120573, Mephitis: 0.344303) '0.252-0.414': 0.129147) '0.396-0.602': 0.101709, (Manis_pentadactyla: 0.337129, Manis_tricuspis: 0.337129) '0.027-0.577': 0.238031) '0.432-0.833': 0.189877) '0.577-1.159': 0.118954, (((((Craseonycteris: 0.228733, Megaderma: 0.228733) '0.005-0.427': 0.122805, Rhinopoma: 0.351538) '0.137-0.523': 0.104846, (Hipposideros: 0.393423, Rhinolophus: 0.393423) '0.334-0.508': 0.062962) '0.355-0.569': 0.048514, Nyctimene: 0.504899) '0.381-0.586': 0.056249, (((((Miniopterus: 0.187084, Myotis: 0.187084) '0.003-0.387': 0.105549, Tadarida: 0.292633) '0.051-0.470': 0.078345, Natalus: 0.370978) '0.184-0.533': 0.073216, Myzopoda: 0.444194) '0.283-0.571': 0.078889, ((Emballonuridae: 0.302971, Nycteris: 0.302971) '0.021-0.519': 0.170899, ((((Furipterus: 0.206030, Noctilio: 0.206030) '0.005-0.396': 0.111227, (Pteronotus: 0.209092, Artibeus: 0.209092) '0.006-0.398': 0.108164) '0.119-0.472': 0.052585, Thyroptera: 0.369842) '0.207-0.523': 0.050493, Mystacina: 0.420334) '0.280-0.556': 0.053535) '0.339-0.578': 0.049213) '0.401-0.590': 0.038065) '0.479-0.597': 0.322842) '0.615-1.274': 0.128836, ((((Erinaceus: 0.348550, Podogymnura: 0.348550) '0.025-0.590': 0.282762, Sorex: 0.631312) '0.483-0.997': 0.087028, Talpa: 0.718340) '0.515-1.187': 0.119344, Solenodon: 0.837684) '0.550-1.268': 0.175142) '0.704-1.320': 0.098503, ((((Cynocephalus: 0.367929, Galeopterus: 0.367929) '0.035-0.623': 0.326229, ((Callithrix: 0.440084, Tarsius: 0.440084) '0.337-0.560': 0.142268, ((((Microcebus: 0.210617, Propithecus: 0.210617) '0.005-0.407': 0.109548, Lemur: 0.320164) '0.088-0.489': 0.081736, Daubentonia: 0.401900) '0.240-0.537': 0.088108, (Otolemur: 0.308772, Nycticebus: 0.308772) '0.018-0.517': 0.181235) '0.386-0.561': 0.092344) '0.559-0.615': 0.111806) '0.567-1.015': 0.185661, (Ptilocercus: 0.390787, Tupaia: 0.390787) '0.024-0.793': 0.489031) '0.619-1.245': 0.145501, ((Leporidae: 0.387731, Ochotona: 0.387731) '0.041-0.732': 0.449279, ((((((((Abrocoma: 0.364835, ((Capromys: 0.263219, (Myocastor: 0.168746, Hoplomys: 0.168746) '0.003-0.360': 0.094473) '0.045-0.406': 0.064085, (Ctenomys: 0.213206, Octodontomys: 0.213206) '0.005-0.392': 0.114098) '0.174-0.447': 0.037531) '0.246-0.476': 0.040998, (Chinchilla: 0.269559, Dinomys: 0.269559) '0.012-0.442': 0.136274) '0.298-0.511': 0.039583, ((Agouti: 0.298194, (Cavia: 0.192899, Dasyprocta: 0.192899) '0.003-0.391': 0.105295) '0.064-0.450': 0.074628, Erethizon: 0.372822) '0.210-0.507': 0.072595) '0.345-0.541': 0.031630, (Heterocephalus: 0.366898, (Petromus: 0.240644, Thryonomys: 0.240644) '0.007-0.436': 0.126255) '0.164-0.518': 0.110148) '0.381-0.562': 0.030078, Hystrix: 0.507125) '0.407-0.577': 0.026176, (Ctenodactylus: 0.329259, Laonastes: 0.329259) '0.018-0.547': 0.204042) '0.444-0.585': 0.030599, (((Anomalurus: 0.294242, Pedetes: 0.294242) '0.020-0.498': 0.159962, (((((Cricetus: 0.157529, Mus: 0.157529) '0.002-0.355': 0.089851, Petromyscus: 0.247380) '0.026-0.402': 0.061560, Calomyscidae: 0.308941) '0.115-0.455': 0.049120, Spalax: 0.358060) '0.194-0.495': 0.046779, Jaculus: 0.404839) '0.269-0.526': 0.049365) '0.330-0.549': 0.051366, (Geomyidae: 0.315927, Dipodomys: 0.315927) '0.018-0.521': 0.189644) '0.416-0.561': 0.058330) '0.527-0.592': 0.029406, ((Aplodontia: 0.277667, Sciuridae: 0.277667) '0.011-0.524': 0.154933, Gliridae: 0.432600) '0.200-0.589': 0.160706) '0.562-0.616': 0.243705) '0.610-1.231': 0.188309) '0.716-1.320': 0.086009) '0.777-1.345': 0.066420) '0.857-1.371': 0.308267) '1.245-1.711': 0.376553, (Ornithorhynchus: 0.452811, Tachyglossus: 0.452811) '0.041-1.235': 1.409757) '1.635-2.086': 1.422768, ((Gallus: 0.765150, Taeniopygia: 0.765150) '0.660-

0.865': 2.014275, Anolis: 2.779424) '2.560-2.997': 0.505912) '3.121-3.472': 0.272709, Xenopus: 3.558045)
'3.315-3.772': 0.649109, Danio: 4.207154) '4.160-4.254';

Joint marginal prior for the 122-taxon dR40 trees with key calibration bounds from Springer et al. (2017)

Lorisiformes 38.0-56.0 Ma, Lagomorpha 53.7-61.6 Ma, Emballonuroidea 47.8-59.2 Ma and Erinaceidae-Soricidae 61.6-83.8 Ma. This calibration scheme also incorporates Springer et al.'s [2] maximum bounds for the basal crown divergences of Chiroptera and Primates, and for the three deepest rodent clades, with each pushed back to 66 Ma, as well as for the Haplorhini maximum bound being pushed back from 57 Ma to 59.2 Ma.

A. Autocorrelated rates

(((((((((Didelphis: 0.195520, Monodelphis: 0.195520) '0.116-0.284': 0.158588, Glironia: 0.354108) '0.162-0.580':
0.134476, Caluromys: 0.488584) '0.258-0.658': 0.222613, (Dromiciops: 0.645431, ((Notoryctes: 0.510520,
(((Isoodon: 0.128759, Echymipera: 0.128759) '0.041-0.235': 0.148274, Macrotis: 0.277033) '0.092-0.493':
0.144777, (Dasyurus: 0.284909, Myrmecobius: 0.284909) '0.155-0.488': 0.136902) '0.256-0.602': 0.088709)
'0.327-0.648': 0.095346, ((((Acrobates: 0.414804, ((Petaurus: 0.305615, Pseudochirops: 0.305615) '0.245-0.427':
0.055536, Tarsipes: 0.361151) '0.262-0.512': 0.053654) '0.289-0.566': 0.060646, ((Macropus: 0.271939,
Aepyprymnus: 0.271939) '0.171-0.435': 0.102681, Hypsiprymnodon: 0.374621) '0.251-0.526': 0.100829) '0.339-
0.606': 0.046912, (Cercartetus: 0.368985, Trichosurus: 0.368985) '0.249-0.533': 0.153376) '0.377-0.635':
0.040526, Phascolarctos: 0.562887) '0.411-0.667': 0.042978) '0.474-0.750': 0.039565) '0.528-0.789': 0.065767)
'0.579-0.822': 0.069678, (Caenolestes: 0.082407, Rhyncholestes: 0.082407) '0.004-0.161': 0.698468) '0.647-
0.849': 0.701888, (((((Echinops: 0.430631, (Amblysomus: 0.272866, Chrysochloris: 0.272866) '0.014-0.553':
0.157765) '0.164-0.649': 0.159650, (Elephantulus: 0.357969, Rhynchocyon: 0.357969) '0.042-0.633': 0.232312)
'0.350-0.911': 0.237946, (Heterohyrax: 0.411580, Procavia: 0.411580) '0.036-0.752': 0.416648) '0.579-1.217':
0.175955, (Dasypus: 0.720118, ((Choloepus: 0.273418, Bradypus: 0.273418) '0.159-0.407': 0.183774, Cyclopes:
0.457191) '0.237-0.742': 0.262926) '0.469-1.192': 0.284065) '0.636-1.311': 0.172846, ((((Tragulus: 0.751946,
(((((Prionodon: 0.217797, Felis: 0.217797) '0.009-0.381': 0.112494, ((Fossa: 0.173017, Suricata: 0.173017) '0.003-
0.357': 0.093434, Genetta: 0.266451) '0.042-0.393': 0.063840) '0.191-0.408': 0.041489, Nandinia: 0.371781)
'0.292-0.416': 0.116458, ((Ailurus: 0.223736, Procyon: 0.223736) '0.009-0.383': 0.119403, Mephitis: 0.343139)
'0.253-0.413': 0.145100) '0.398-0.621': 0.105098, (Manis_pentadactyla: 0.355658, Manis_tricuspis: 0.355658)
'0.025-0.615': 0.237679) '0.448-0.817': 0.158609) '0.585-1.124': 0.113600, (((((Craseonycteris: 0.248419,
Megaderma: 0.248419) '0.011-0.489': 0.136239, Rhinopoma: 0.384659) '0.155-0.576': 0.122529, (Hipposideros:
0.409057, Rhinolophus: 0.409057) '0.336-0.523': 0.098130) '0.365-0.628': 0.061979, Nyctimene: 0.569166)
'0.412-0.652': 0.063184, (((((Miniopterus: 0.217831, Myotis: 0.217831) '0.007-0.433': 0.115626, Tadarida:
0.333457) '0.086-0.547': 0.093372, Natalus: 0.426828) '0.230-0.607': 0.090335, Myzopoda: 0.517164) '0.311-
0.637': 0.086670, ((Emballonuridae: 0.521987, Nycteris: 0.521987) '0.477-0.588': 0.052498, ((((Furipterus:
0.233159, Noctilio: 0.233159) '0.013-0.440': 0.125913, (Pteronotus: 0.233332, Artibeus: 0.233332) '0.011-0.442':
0.125739) '0.171-0.539': 0.069688, Thyroptera: 0.428759) '0.254-0.583': 0.072365, Mystacina: 0.501124) '0.335-
0.620': 0.073362) '0.496-0.643': 0.029349) '0.518-0.657': 0.028516) '0.564-0.668': 0.233196) '0.640-1.239':
0.138215, ((((Erinaceus: 0.378990, Podogymnura: 0.378990) '0.027-0.636': 0.286675, Sorex: 0.665665) '0.604-
0.815': 0.076205, Talpa: 0.741870) '0.614-1.108': 0.107710, Solenodon: 0.849581) '0.626-1.240': 0.154180)
'0.710-1.306': 0.103572, ((((Cynocephalus: 0.394123, Galeopterus: 0.394123) '0.043-0.651': 0.286321,
((Callithrix: 0.450553, Tarsius: 0.450553) '0.338-0.582': 0.145539, ((((Microcebus: 0.213050, Propithecus:
0.213050) '0.006-0.417': 0.114294, Lemur: 0.327344) '0.093-0.507': 0.089948, Daubentonia: 0.417292) '0.247-
0.547': 0.094853, (Otolemur: 0.441724, Nycticebus: 0.441724) '0.377-0.538': 0.070420) '0.415-0.564': 0.083947)
'0.559-0.654': 0.084352) '0.572-0.965': 0.193546, (Ptilocercus: 0.416520, Tupaia: 0.416520) '0.029-0.781':
0.457470) '0.625-1.241': 0.148316, ((Leporidae: 0.575215, Ochotona: 0.575215) '0.537-0.616': 0.270914,
(((((((Abrocoma: 0.388177, ((Capromys: 0.276069, (Myocastor: 0.178018, Hoplomys: 0.178018) '0.004-0.375':

0.098051) '0.057-0.430': 0.068648, (Ctenomys: 0.227023, Octodontomys: 0.227023) '0.010-0.411': 0.117694)
'0.178-0.495': 0.043460) '0.252-0.531': 0.048670, (Chinchilla: 0.282836, Dinomys: 0.282836) '0.018-0.492':
0.154011) '0.308-0.563': 0.050478, ((Agouti: 0.317446, (Cavia: 0.204590, Dasyprocta: 0.204590) '0.004-0.406':
0.112856) '0.094-0.501': 0.085260, Erethizon: 0.402706) '0.227-0.556': 0.084618) '0.365-0.596': 0.036432,
(Heterocephalus: 0.397858, (Petromus: 0.258201, Thryonomys: 0.258201) '0.009-0.493': 0.139657) '0.174-
0.571': 0.125899) '0.396-0.619': 0.034783, Hystrix: 0.558540) '0.440-0.635': 0.027128, (Ctenodactylus: 0.359642,
Laonastes: 0.359642) '0.023-0.606': 0.226026) '0.493-0.646': 0.027833, (((Anomalurus: 0.322426, Pedetes:
0.322426) '0.022-0.566': 0.190317, (((((Cricetus: 0.172748, Mus: 0.172748) '0.005-0.378': 0.096340,
Petromyscus: 0.269089) '0.053-0.453': 0.068327, Calomyscidae: 0.337416) '0.141-0.519': 0.059151, Spalax:
0.396567) '0.223-0.567': 0.058985, Jaculus: 0.455552) '0.292-0.600': 0.057191) '0.362-0.625': 0.051419,
(Geomyidae: 0.352938, Dipodomys: 0.352938) '0.038-0.592': 0.211224) '0.437-0.639': 0.049339) '0.549-0.656':
0.023729, ((Aplodontia: 0.302420, Sciuridae: 0.302420) '0.022-0.577': 0.170863, Gliridae: 0.473284) '0.198-
0.642': 0.163947) '0.582-0.663': 0.208898) '0.639-1.216': 0.176178) '0.728-1.311': 0.085026) '0.795-1.340':
0.069696) '0.876-1.369': 0.305735) '1.245-1.711': 0.380003, (Ornithorhynchus: 0.483498, Tachyglossus:
0.483498) '0.056-1.197': 1.379268) '1.631-2.085': 1.422644, ((Gallus: 0.764272, Taeniopygia: 0.764272) '0.660-
0.866': 2.014209, Anolis: 2.778482) '2.560-2.999': 0.506929) '3.122-3.469': 0.273478, Xenopus: 3.558889)
'3.312-3.773': 0.647959, Danio: 4.206847) '4.160-4.254';

B. Independent rates

(((((((((Didelphis: 0.195481, Monodelphis: 0.195481) '0.116-0.284': 0.159265, Glironia: 0.354745) '0.159-0.583':
0.134760, Caluromys: 0.489505) '0.255-0.658': 0.220208, (Dromiciops: 0.644305, ((Notoryctes: 0.511742,
(((Isoodon: 0.129137, Echymipera: 0.129137) '0.042-0.235': 0.145748, Macrotis: 0.274885) '0.091-0.493':
0.148576, (Dasyurus: 0.283935, Myrmecobius: 0.283935) '0.150-0.493': 0.139526) '0.252-0.597': 0.088281)
'0.322-0.649': 0.093267, ((((Acrobates: 0.418787, ((Petaurus: 0.305879, Pseudochirops: 0.305879) '0.243-0.440':
0.057022, Tarsipes: 0.362901) '0.260-0.518': 0.055885) '0.280-0.570': 0.060713, ((Macropus: 0.272471,
Aepyprymnus: 0.272471) '0.171-0.439': 0.105919, Hypsiprymnodon: 0.378390) '0.254-0.531': 0.101110) '0.339-
0.607': 0.044068, (Cercartetus: 0.368988, Trichosurus: 0.368988) '0.249-0.534': 0.154580) '0.376-0.635':
0.039694, Phascolarctos: 0.563262) '0.409-0.677': 0.041747) '0.480-0.742': 0.039296) '0.527-0.786': 0.065408)
'0.575-0.822': 0.071393, (Caenolestes: 0.083040, Rhyncholestes: 0.083040) '0.005-0.160': 0.698065) '0.642-
0.849': 0.703651, (((((Echinops: 0.434313, (Amblysomus: 0.274373, Chrysochloris: 0.274373) '0.014-0.557':
0.159940) '0.169-0.655': 0.157768, (Elephantulus: 0.354997, Rhynchocyon: 0.354997) '0.035-0.630': 0.237084)
'0.341-0.927': 0.243755, (Heterohyrax: 0.412787, Procavia: 0.412787) '0.040-0.789': 0.423049) '0.576-1.226':
0.175160, (Dasypus: 0.721454, ((Choloepus: 0.272600, Bradypus: 0.272600) '0.159-0.409': 0.185343, Cyclopes:
0.457943) '0.241-0.766': 0.263511) '0.467-1.184': 0.289542) '0.636-1.307': 0.167421, ((((Tragulus: 0.758345,
(((((Prionodon: 0.220428, Felis: 0.220428) '0.010-0.384': 0.111120, ((Fossa: 0.176354, Suricata: 0.176354) '0.005-
0.356': 0.092227, Genetta: 0.268580) '0.071-0.393': 0.062968) '0.198-0.409': 0.041059, Nandinia: 0.372607)
'0.291-0.416': 0.119648, ((Ailurus: 0.222820, Procyon: 0.222820) '0.011-0.387': 0.119270, Mephitis: 0.342090)
'0.252-0.414': 0.150165) '0.398-0.624': 0.102967, (Manis_pentadactyla: 0.360199, Manis_tricuspis: 0.360199)
'0.029-0.617': 0.235023) '0.448-0.805': 0.163123) '0.590-1.145': 0.118134, (((((Craseonycteris: 0.244736,
Megaderma: 0.244736) '0.010-0.487': 0.141190, Rhinopoma: 0.385926) '0.151-0.577': 0.121573, (Hipposideros:
0.409436, Rhinolophus: 0.409436) '0.335-0.525': 0.098062) '0.366-0.630': 0.062268, Nyctimene: 0.569767)
'0.418-0.653': 0.063746, (((((Miniopterus: 0.222351, Myotis: 0.222351) '0.008-0.479': 0.116168, Tadarida:
0.338519) '0.093-0.552': 0.093736, Natalus: 0.432254) '0.229-0.607': 0.089251, Myzopoda: 0.521505) '0.327-
0.640': 0.084319, ((Emballonuridae: 0.522176, Nycteris: 0.522176) '0.476-0.587': 0.053337, ((((Furipterus:
0.235563, Noctilio: 0.235563) '0.010-0.456': 0.128369, (Pteronotus: 0.234839, Artibeus: 0.234839) '0.008-0.457':
0.129093) '0.161-0.548': 0.070292, Thyroptera: 0.434225) '0.257-0.591': 0.070566, Mystacina: 0.504790) '0.339-
0.623': 0.070722) '0.497-0.645': 0.030311) '0.526-0.658': 0.027688) '0.564-0.668': 0.242967) '0.646-1.234':
0.133283, ((((Erinaceus: 0.390314, Podogymnura: 0.390314) '0.039-0.635': 0.275443, Sorex: 0.665757) '0.603-
0.815': 0.075035, Talpa: 0.740792) '0.614-1.097': 0.110217, Solenodon: 0.851009) '0.629-1.229': 0.158753)
'0.713-1.292': 0.100244, ((((Cynocephalus: 0.392361, Galeopterus: 0.392361) '0.040-0.650': 0.287593,
((Callithrix: 0.451432, Tarsius: 0.451432) '0.338-0.583': 0.144120, ((((Microcebus: 0.215827, Propithecus:
0.215827) '0.008-0.425': 0.114283, Lemur: 0.330110) '0.104-0.517': 0.090945, Daubentonia: 0.421055) '0.242-
0.548': 0.092051, (Otolemur: 0.442749, Nycticebus: 0.442749) '0.377-0.539': 0.070356) '0.418-0.565': 0.082446)
'0.559-0.653': 0.084401) '0.573-0.988': 0.192312, (Ptilocercus: 0.419855, Tupaia: 0.419855) '0.045-0.774':
0.452411) '0.621-1.224': 0.149400, ((Leporidae: 0.574827, Ochotona: 0.574827) '0.537-0.616': 0.275152,
(((((((Abrocoma: 0.397839, ((Capromys: 0.281811, (Myocastor: 0.181579, Hoplomys: 0.181579) '0.005-0.385':

0.100232) '0.065-0.466': 0.071657, (Ctenomys: 0.233500, Octodontomys: 0.233500) '0.011-0.447': 0.119968) '0.176-0.524': 0.044371) '0.253-0.550': 0.048546, (Chinchilla: 0.290490, Dinomys: 0.290490) '0.021-0.520': 0.155894) '0.310-0.576': 0.048723, ((Agouti: 0.325005, (Cavia: 0.211326, Dasyprocta: 0.211326) '0.006-0.437': 0.113678) '0.099-0.526': 0.085863, Erethizon: 0.410867) '0.230-0.564': 0.084240) '0.361-0.603': 0.033960, (Heterocephalus: 0.403014, (Petromus: 0.260516, Thryonomys: 0.260516) '0.015-0.504': 0.142498) '0.161-0.577': 0.126054) '0.403-0.621': 0.032645, Hystrix: 0.561713) '0.449-0.635': 0.025412, (Ctenodactylus: 0.366230, Laonastes: 0.366230) '0.040-0.608': 0.220895) '0.496-0.646': 0.027235, (((Anomalurus: 0.325523, Pedetes: 0.325523) '0.025-0.566': 0.189922, (((((Cricetus: 0.170660, Mus: 0.170660) '0.004-0.381': 0.095817, Petromyscus: 0.266478) '0.050-0.454': 0.070200, Calomyscidae: 0.336678) '0.132-0.524': 0.061474, Spalax: 0.398152) '0.217-0.569': 0.058897, Jaculus: 0.457049) '0.284-0.602': 0.058396) '0.357-0.623': 0.050904, (Geomyidae: 0.353446, Dipodomys: 0.353446) '0.032-0.595': 0.212903) '0.437-0.640': 0.048012) '0.548-0.656': 0.023782, ((Aplodontia: 0.302881, Sciuridae: 0.302881) '0.019-0.577': 0.173966, Gliridae: 0.476847) '0.202-0.644': 0.161296) '0.584-0.663': 0.211836) '0.639-1.195': 0.171688) '0.725-1.288': 0.088340) '0.793-1.329': 0.068411) '0.878-1.368': 0.306340) '1.247-1.711': 0.376933, (Ornithorhynchus: 0.478812, Tachyglossus: 0.478812) '0.043-1.199': 1.382878) '1.632-2.085': 1.423437, ((Gallus: 0.765059, Taeniopygia: 0.765059) '0.659-0.865': 2.011795, Anolis: 2.776854) '2.559-2.997': 0.508273) '3.122-3.471': 0.273381, Xenopus: 3.558508) '3.318-3.771': 0.648851, Danio: 4.207359) '4.160-4.254';

MCMCtree timetrees

Trees can be opened in Figtree, and include mean and 95% credible intervals.

122-taxon dR32 trees

These analyses show that retaining the calibration scheme from Phillips [1], but deleting large, long-lived taxa does not increase the age estimates for the placental interordinal diversification, contrary to the claim of Springer et al. [2]. The soft explosive model is supported.

A. Autocorrelated rates

(((((((((Didelphis: 0.225793, Monodelphis: 0.225793) '0.199-0.272': 0.059274, Glironia: 0.285068) '0.258-0.328': 0.018402, Caluromys: 0.303469) '0.275-0.351': 0.368758, (Dromiciops: 0.585299, ((Notoryctes: 0.565264, (((Isoodon: 0.104434, Echymipera: 0.104434) '0.086-0.123': 0.154303, Macrotis: 0.258737) '0.232-0.289': 0.293074, (Dasyurus: 0.272207, Myrmecobius: 0.272207) '0.241-0.310': 0.279605) '0.528-0.581': 0.013453) '0.541-0.592': 0.004328, ((((Acrobates: 0.392512, ((Petaurus: 0.327201, Pseudochirops: 0.327201) '0.306-0.349': 0.024607, Tarsipes: 0.351808) '0.330-0.373': 0.040704) '0.371-0.414': 0.046153, ((Macropus: 0.162976, Aepyprymnus: 0.162976) '0.145-0.182': 0.104771, Hypsiprymnodon: 0.267747) '0.249-0.290': 0.170918) '0.415-0.459': 0.011181, (Cercartetus: 0.420263, Trichosurus: 0.420263) '0.394-0.445': 0.029583) '0.426-0.471': 0.046567, Phascolarctos: 0.496413) '0.468-0.522': 0.073179) '0.546-0.596': 0.015706) '0.560-0.613': 0.086929) '0.650-0.704': 0.049083, (Caenolestes: 0.090828, Rhyncholestes: 0.090828) '0.070-0.120': 0.630483) '0.689-0.758': 0.961479, (((((Echinops: 0.545452, (Amblysomus: 0.084993, Chrysochloris: 0.084993) '0.064-0.108': 0.460459) '0.521-0.577': 0.041587, (Elephantulus: 0.398108, Rhynchocyon: 0.398108) '0.358-0.441': 0.188931) '0.568-0.615': 0.017973, (Heterohyrax: 0.030911, Procavia: 0.030911) '0.023-0.041': 0.574102) '0.585-0.632': 0.154675, (Dasypus: 0.523851, ((Choloepus: 0.165220, Bradypus: 0.165220) '0.122-0.204': 0.287068, Cyclopes: 0.452288) '0.424-0.478': 0.071564) '0.495-0.552': 0.235837) '0.741-0.778': 0.006181, ((((Tragulus: 0.648857, (((((Prionodon: 0.248357, Felis: 0.248357) '0.227-0.267': 0.028758, ((Fossa: 0.166411, Suricata: 0.166411) '0.143-0.186': 0.100775, Genetta: 0.267186) '0.247-0.286': 0.009929) '0.256-0.295': 0.058024, Nandinia: 0.335138) '0.312-0.356': 0.106053, ((Ailurus: 0.248037, Procyon: 0.248037) '0.224-0.269': 0.012809, Mephitis: 0.260846) '0.237-0.282': 0.180345) '0.413-0.463': 0.197781, (Manis_pentadactyla: 0.172333, Manis_tricuspis: 0.172333) '0.134-0.214': 0.466639) '0.622-0.654': 0.009885) '0.633-0.663': 0.001570, (((((Craseonycteris: 0.392676,

Megaderma: 0.392676) '0.367-0.416': 0.049823, Rhinopoma: 0.442499) '0.424-0.460': 0.015647, (Hipposideros: 0.365292, Rhinolophus: 0.365292) '0.342-0.388': 0.092854) '0.440-0.476': 0.060514, Nyctimene: 0.518660) '0.502-0.534': 0.020605, (((((Miniopterus: 0.380532, Myotis: 0.380532) '0.361-0.402': 0.025317, Tadarida: 0.405849) '0.389-0.424': 0.015177, Natalus: 0.421026) '0.405-0.439': 0.019684, Myzopoda: 0.440710) '0.425-0.458': 0.014252, ((Emballonuridae: 0.417772, Nycteris: 0.417772) '0.399-0.438': 0.035467, ((((Furipterus: 0.324173, Noctilio: 0.324173) '0.300-0.346': 0.037826, (Pteronotus: 0.320829, Artibeus: 0.320829) '0.298-0.343': 0.041170) '0.343-0.380': 0.005878, Thyroptera: 0.367878) '0.349-0.386': 0.028195, Mystacina: 0.396072) '0.379-0.413': 0.057166) '0.438-0.469': 0.001723) '0.440-0.471': 0.084303) '0.523-0.554': 0.111161) '0.634-0.665': 0.012790, ((((Erinaceus: 0.273810, Podogymnura: 0.273810) '0.246-0.306': 0.298336, Sorex: 0.572145) '0.552-0.592': 0.034081, Talpa: 0.606226) '0.588-0.622': 0.006378, Solenodon: 0.612604) '0.595-0.628': 0.050611) '0.648-0.677': 0.057303, ((((Cynocephalus: 0.054391, Galeopterus: 0.054391) '0.041-0.070': 0.605614, ((Callithrix: 0.585459, Tarsius: 0.585459) '0.571-0.598': 0.029898, ((((Microcebus: 0.254200, Propithecus: 0.254200) '0.234-0.272': 0.007886, Lemur: 0.262086) '0.242-0.281': 0.171969, Daubentonia: 0.434054) '0.416-0.451': 0.029292, (Otolemur: 0.187138, Nycticebus: 0.187138) '0.163-0.213': 0.276208) '0.447-0.480': 0.152011) '0.605-0.623': 0.044647) '0.648-0.671': 0.002654, (Ptilocercus: 0.446629, Tupaia: 0.446629) '0.413-0.476': 0.216030) '0.651-0.674': 0.002680, ((Leporidae: 0.382694, Ochotona: 0.382694) '0.344-0.419': 0.261234, ((((((((Abrocoma: 0.177686, ((Capromys: 0.119218, (Myocastor: 0.090269, Hoplomys: 0.090269) '0.081-0.099': 0.028949) '0.109-0.129': 0.046303, (Ctenomys: 0.144720, Octodontomys: 0.144720) '0.133-0.157': 0.020802) '0.155-0.177': 0.012165) '0.166-0.190': 0.088020, (Chinchilla: 0.204700, Dinomys: 0.204700) '0.189-0.221': 0.061006) '0.253-0.279': 0.021779, ((Agouti: 0.206020, (Cavia: 0.197809, Dasyprocta: 0.197809) '0.185-0.211': 0.008211) '0.193-0.219': 0.075017, Erethizon: 0.281037) '0.268-0.295': 0.006448) '0.274-0.301': 0.057042, (Heterocephalus: 0.310327, (Petromus: 0.176477, Thryonomys: 0.176477) '0.159-0.195': 0.133850) '0.296-0.325': 0.034200) '0.330-0.359': 0.018782, Hystrix: 0.363309) '0.348-0.378': 0.141652, (Ctenodactylus: 0.350542, Laonastes: 0.350542) '0.325-0.375': 0.154419) '0.488-0.520': 0.073922, (((Anomalurus: 0.458609, Pedetes: 0.458609) '0.438-0.479': 0.085860, (((((Cricetus: 0.197007, Mus: 0.197007) '0.180-0.215': 0.017095, Petromyscus: 0.214102) '0.198-0.231': 0.010969, Calomyscidae: 0.225070) '0.205-0.247': 0.144544, Spalax: 0.369615) '0.353-0.386': 0.083935, Jaculus: 0.453550) '0.438-0.469': 0.090919) '0.531-0.555': 0.006950, (Geomyidae: 0.247361, Dipodomys: 0.247361) '0.212-0.276': 0.304058) '0.538-0.562': 0.027465) '0.565-0.589': 0.003386, ((Aplodontia: 0.430150, Sciuridae: 0.430150) '0.402-0.454': 0.111727, Gliridae: 0.541877) '0.528-0.555': 0.040393) '0.569-0.593': 0.061659) '0.630-0.656': 0.021410) '0.653-0.676': 0.055180) '0.706-0.734': 0.045351) '0.748-0.782': 0.916921) '1.628-1.724': 0.229552, (Ornithorhynchus: 0.306211, Tachyglossus: 0.306211) '0.185-0.455': 1.606131) '1.875-1.950': 1.216086, ((Gallus: 0.748558, Taeniopygia: 0.748558) '0.656-0.865': 1.960218, Anolis: 2.708776) '2.561-2.865': 0.419653) '3.083-3.186': 0.598344, Xenopus: 3.726773) '3.606-3.796': 0.476286, Danio: 4.203058) '4.159-4.253': 0.013211;

B. Independent rates

(((((((((Didelphis: 0.155450, Monodelphis: 0.155450) '0.135-0.178': 0.038292, Glironia: 0.193741) '0.173-0.217': 0.013662, Caluromys: 0.207404) '0.186-0.231': 0.316061, (Dromiciops: 0.425764, ((Notoryctes: 0.408119, (((Isoodon: 0.081613, Echymipera: 0.081613) '0.069-0.096': 0.095572, Macrotis: 0.177185) '0.158-0.199': 0.221825, (Dasyurus: 0.192911, Myrmecobius: 0.192911) '0.168-0.220': 0.206098) '0.377-0.422': 0.009109) '0.387-0.432': 0.003552, ((((Acrobates: 0.267897, ((Petaurus: 0.226876, Pseudochirops: 0.226876) '0.207-0.248': 0.018267, Tarsipes: 0.245143) '0.226-0.266': 0.022754) '0.249-0.289': 0.032031, ((Macropus: 0.115234, Aepyprymnus: 0.115234) '0.101-0.131': 0.072930, Hypsiprymnodon: 0.188165) '0.171-0.208': 0.111763) '0.281-0.321': 0.007415, (Cercartetus: 0.285693, Trichosurus: 0.285693) '0.264-0.310': 0.021649) '0.288-0.329': 0.032620, Phascolarctos: 0.339963) '0.320-0.363': 0.071708) '0.391-0.434': 0.014094) '0.405-0.449': 0.097700) '0.493-0.552': 0.034257, (Caenolestes: 0.070224, Rhyncholestes: 0.070224) '0.058-0.084': 0.487498) '0.525-0.592': 1.013111, (((((Echinops: 0.552108, (Amblysomus: 0.075246, Chrysochloris: 0.075246) '0.062-0.089': 0.476862) '0.525-0.585': 0.046310, (Elephantulus: 0.420020, Rhynchocyon: 0.420020) '0.389-0.455': 0.178398) '0.578-0.628': 0.020004, (Heterohyrax: 0.030280, Procavia: 0.030280) '0.024-0.037': 0.588142) '0.595-0.647': 0.132019, (Dasypus: 0.439728, ((Choloepus: 0.128063, Bradypus: 0.128063) '0.108-0.152': 0.252178, Cyclopes: 0.380241) '0.348-0.419': 0.059487) '0.413-0.480': 0.310713) '0.729-0.774': 0.005950, ((((Tragulus: 0.629806, (((((Prionodon: 0.160519, Felis: 0.160519) '0.143-0.179': 0.027340, ((Fossa: 0.119131, Suricata: 0.119131) '0.103-0.136': 0.061884, Genetta: 0.181015) '0.164-0.199': 0.006844) '0.172-0.205': 0.034617, Nandinia: 0.222476) '0.204-0.243': 0.152186, ((Ailurus: 0.201834, Procyon: 0.201834) '0.175-0.228': 0.010832, Mephitis: 0.212665) '0.186-0.239': 0.161996) '0.339-0.404': 0.239878, (Manis_pentadactyla: 0.151845, Manis_tricuspis: 0.151845) '0.124-0.185': 0.462694) '0.592-0.637': 0.015266) '0.610-0.650': 0.001321, (((((Craseonycteris: 0.353852,

Megaderma: 0.353852) '0.322-0.385': 0.029835, Rhinopoma: 0.383686) '0.354-0.412': 0.016041, (Hipposideros: 0.280733, Rhinolophus: 0.280733) '0.243-0.315': 0.118994) '0.370-0.426': 0.048357, Nyctimene: 0.448084) '0.421-0.476': 0.031233, (((((Miniopterus: 0.339740, Myotis: 0.339740) '0.312-0.364': 0.025603, Tadarida: 0.365343) '0.341-0.388': 0.011106, Natalus: 0.376449) '0.354-0.399': 0.021842, Myzopoda: 0.398291) '0.376-0.420': 0.009444, ((Emballonuridae: 0.372801, Nycteris: 0.372801) '0.348-0.397': 0.033293, ((((Furipterus: 0.297682, Noctilio: 0.297682) '0.271-0.322': 0.030421, (Pteronotus: 0.292646, Artibeus: 0.292646) '0.267-0.315': 0.035457) '0.305-0.349': 0.004864, Thyroptera: 0.332967) '0.310-0.354': 0.021929, Mystacina: 0.354896) '0.331-0.376': 0.051198) '0.384-0.427': 0.001641) '0.386-0.428': 0.071581) '0.453-0.504': 0.151810) '0.611-0.651': 0.015012, ((((Erinaceus: 0.317130, Podogymnura: 0.317130) '0.284-0.352': 0.239094, Sorex: 0.556223) '0.535-0.577': 0.032994, Talpa: 0.589217) '0.567-0.607': 0.005501, Solenodon: 0.594718) '0.573-0.613': 0.051420) '0.627-0.665': 0.063062, ((((Cynocephalus: 0.051007, Galeopterus: 0.051007) '0.041-0.063': 0.603328, ((Callithrix: 0.571533, Tarsius: 0.571533) '0.547-0.593': 0.029987, ((((Microcebus: 0.169422, Propithecus: 0.169422) '0.147-0.194': 0.008385, Lemur: 0.177807) '0.155-0.202': 0.165274, Daubentonia: 0.343081) '0.307-0.387': 0.031036, (Otolemur: 0.144093, Nycticebus: 0.144093) '0.122-0.171': 0.230025) '0.339-0.417': 0.227403) '0.579-0.617': 0.052814) '0.639-0.670': 0.003367, (Ptilocercus: 0.427008, Tupaia: 0.427008) '0.390-0.473': 0.230694) '0.643-0.673': 0.003428, ((Leporidae: 0.397298, Ochotona: 0.397298) '0.359-0.432': 0.245265, (((((((Abrocoma: 0.226594, ((Capromys: 0.155130, (Myocastor: 0.121917, Hoplomys: 0.121917) '0.107-0.137': 0.033213) '0.140-0.171': 0.059396, (Ctenomys: 0.178476, Octodontomys: 0.178476) '0.161-0.195': 0.036050) '0.199-0.230': 0.012069) '0.211-0.242': 0.096936, (Chinchilla: 0.226385, Dinomys: 0.226385) '0.199-0.254': 0.097145) '0.307-0.342': 0.021857, ((Agouti: 0.243825, (Cavia: 0.234417, Dasyprocta: 0.234417) '0.213-0.259': 0.009408) '0.222-0.269': 0.091529, Erethizon: 0.335354) '0.317-0.355': 0.010033) '0.329-0.364': 0.057056, (Heterocephalus: 0.358678, (Petromus: 0.229303, Thryonomys: 0.229303) '0.206-0.254': 0.129375) '0.339-0.379': 0.043765) '0.385-0.420': 0.016882, Hystrix: 0.419325) '0.402-0.437': 0.113958, (Ctenodactylus: 0.379758, Laonastes: 0.379758) '0.348-0.409': 0.153525) '0.519-0.548': 0.057851, (((Anomalurus: 0.430519, Pedetes: 0.430519) '0.400-0.462': 0.124886, (((((Cricetus: 0.248813, Mus: 0.248813) '0.225-0.273': 0.022995, Petromyscus: 0.271808) '0.248-0.296': 0.011399, Calomyscidae: 0.283207) '0.259-0.310': 0.122351, Spalax: 0.405558) '0.385-0.427': 0.080066, Jaculus: 0.485624) '0.468-0.505': 0.069782) '0.545-0.567': 0.007797, (Geomyidae: 0.285534, Dipodomys: 0.285534) '0.252-0.321': 0.277669) '0.554-0.574': 0.027931) '0.584-0.601': 0.003423, ((Aplodontia: 0.399454, Sciuridae: 0.399454) '0.362-0.431': 0.149951, Gliridae: 0.549405) '0.529-0.567': 0.045151) '0.587-0.605': 0.048007) '0.630-0.657': 0.018566) '0.648-0.676': 0.048071) '0.693-0.726': 0.047191) '0.736-0.778': 0.814442) '1.475-1.660': 0.307111, (Ornithorhynchus: 0.206921, Tachyglossus: 0.206921) '0.168-0.252': 1.671023) '1.802-1.925': 1.250546, ((Gallus: 0.691875, Taeniopygia: 0.691875) '0.648-0.771': 1.937236, Anolis: 2.629111) '2.544-2.774': 0.499379) '3.085-3.184': 0.605631, Xenopus: 3.734121) '3.621-3.800': 0.481131, Danio: 4.215252) '4.163-4.255': 0.013211;

122-taxon dR40 trees

These analyses add further calibration priors (see Additional file 2: Table S2, dR40). The soft explosive model is supported.

A. Autocorrelated rates

(((((((((Didelphis: 0.260112, Monodelphis: 0.260112) '0.22431-0.284766': 0.069891, Glironia: 0.330004) '0.28679-0.359756': 0.034809, Caluromys: 0.364813) '0.319212-0.397731': 0.349240, (Dromiciops: 0.609496, ((Notoryctes: 0.569710, (((Isoodon: 0.107806, Echymipera: 0.107806) '0.0929593-0.125156': 0.131077, Macrotis: 0.238883) '0.213092-0.267081': 0.305598, (Dasyurus: 0.257221, Myrmecobius: 0.257221) '0.227701-0.286966': 0.287260) '0.508812-0.584631': 0.025229) '0.535422-0.608909': 0.012260, ((((Acrobates: 0.390109, ((Petaurus: 0.308743, Pseudochirops: 0.308743) '0.283028-0.333194': 0.040391, Tarsipes: 0.349134) '0.322405-0.375046': 0.040975) '0.360742-0.417207': 0.052812, ((Macropus: 0.163610, Aepyprymnus: 0.163610) '0.144318-0.180863': 0.117238, Hypsiprymnodon: 0.280848) '0.25711-0.305246': 0.162073) '0.411808-0.472933': 0.013840, (Cercartetus: 0.417031, Trichosurus: 0.417031) '0.386324-0.445954': 0.039730) '0.425086-0.487021': 0.046125, Phascolarctos: 0.502886) '0.470168-0.536813': 0.079084) '0.547112-0.622571': 0.027526) '0.573348-0.652516': 0.104556) '0.672074-0.764701': 0.062695, (Caenolestes: 0.137354, Rhyncholestes: 0.137354) '0.11019-0.160878': 0.639394) '0.72998-0.832748': 0.873086, (((((Echinops: 0.547265, (Amblysomus: 0.111204, Chrysochloris: 0.111204) '0.0925251-0.130007': 0.436062) '0.523119-0.573313': 0.050280, (Elephantulus:

0.422679, Rhynchocyon: 0.422679) '0.395161-0.450212': 0.174866) '0.574312-0.622245': 0.025308, (Heterohyrax: 0.036805, Procavia: 0.036805) '0.0285815-0.0460267': 0.586048) '0.599276-0.646684': 0.151531, (Dasypus: 0.546801, ((Choloepus: 0.196865, Bradypus: 0.196865) '0.174694-0.223486': 0.272804, Cyclopes: 0.469670) '0.441287-0.506904': 0.077132) '0.519503-0.582689': 0.227583) '0.750313-0.797266': 0.014765, ((((Tragulus: 0.650058, (((((Prionodon: 0.229106, Felis: 0.229106) '0.208087-0.253092': 0.051859, ((Fossa: 0.176742, Suricata: 0.176742) '0.158811-0.194712': 0.085559, Genetta: 0.262302) '0.239549-0.283444': 0.018664) '0.258377-0.303313': 0.060706, Nandinia: 0.341671) '0.315258-0.365819': 0.118282, ((Ailurus: 0.252855, Procyon: 0.252855) '0.228548-0.276359': 0.023907, Mephitis: 0.276762) '0.252859-0.299999': 0.183192) '0.436152-0.484451': 0.165630, (Manis_pentadactyla: 0.230899, Manis_tricuspis: 0.230899) '0.194487-0.266491': 0.394685) '0.606796-0.645965': 0.024475) '0.631781-0.669053': 0.006036, (((((Craseonycteris: 0.397694, Megaderma: 0.397694) '0.372851-0.422046': 0.051904, Rhinopoma: 0.449598) '0.42783-0.471687': 0.026281, (Hipposideros: 0.348731, Rhinolophus: 0.348731) '0.331256-0.368338': 0.127147) '0.455352-0.496822': 0.052921, Nyctimene: 0.528799) '0.508294-0.549545': 0.032710, (((((Miniopterus: 0.358267, Myotis: 0.358267) '0.338267-0.381983': 0.040647, Tadarida: 0.398914) '0.37835-0.420911': 0.019202, Natalus: 0.418116) '0.397483-0.439853': 0.029496, Myzopoda: 0.447612) '0.427354-0.469805': 0.014266, ((Emballonuridae: 0.414284, Nycteris: 0.414284) '0.391642-0.438398': 0.042011, ((((Furipterus: 0.313222, Noctilio: 0.313222) '0.292279-0.339523': 0.038511, (Pteronotus: 0.310231, Artibeus: 0.310231) '0.290843-0.335665': 0.041501) '0.333318-0.376189': 0.007924, Thyroptera: 0.359656) '0.341-0.38417': 0.032293, Mystacina: 0.391949) '0.372204-0.415494': 0.064346) '0.436341-0.478637': 0.005583) '0.442214-0.483982': 0.099631) '0.541609-0.582202': 0.094585) '0.637375-0.674325': 0.016426, ((((Erinaceus: 0.262066, Podogymnura: 0.262066) '0.238227-0.284455': 0.279877, Sorex: 0.541943) '0.521195-0.563294': 0.046976, Talpa: 0.588919) '0.568958-0.60746': 0.013892, Solenodon: 0.602811) '0.582836-0.621208': 0.069709) '0.654134-0.689997': 0.063509, ((((Cynocephalus: 0.103474, Galeopterus: 0.103474) '0.0823876-0.12423': 0.559893, ((Callithrix: 0.571992, Tarsius: 0.571992) '0.559061-0.583553': 0.040528, ((((Microcebus: 0.254392, Propithecus: 0.254392) '0.233263-0.274663': 0.018651, Lemur: 0.273043) '0.251136-0.293521': 0.160748, Daubentonia: 0.433791) '0.41331-0.452883': 0.045415, (Otolemur: 0.168485, Nycticebus: 0.168485) '0.149892-0.187105': 0.310722) '0.460576-0.496664': 0.133313) '0.601453-0.619249': 0.050847) '0.648973-0.674996': 0.008995, (Ptilocercus: 0.459783, Tupaia: 0.459783) '0.434267-0.484664': 0.212578) '0.658111-0.68458': 0.007696, ((Leporidae: 0.404273, Ochotona: 0.404273) '0.376159-0.43135': 0.248969, ((((((((Abrocoma: 0.156652, ((Capromys: 0.098578, (Myocastor: 0.077610, Hoplomys: 0.077610) '0.0689708-0.0864426': 0.020968) '0.0886258-0.108502': 0.045898, (Ctenomys: 0.119247, Octodontomys: 0.119247) '0.108424-0.130587': 0.025228) '0.13217-0.157068': 0.012177) '0.143652-0.170058': 0.101945, (Chinchilla: 0.184842, Dinomys: 0.184842) '0.170384-0.200083': 0.073754) '0.243678-0.273776': 0.023932, ((Agouti: 0.192726, (Cavia: 0.180194, Dasyprocta: 0.180194) '0.166787-0.194315': 0.012532) '0.17889-0.207352': 0.078627, Erethizon: 0.271353) '0.25601-0.287213': 0.011175) '0.267049-0.298295': 0.058758, (Heterocephalus: 0.297964, (Petromus: 0.178602, Thryonomys: 0.178602) '0.163421-0.19399': 0.119362) '0.281059-0.314006': 0.043322) '0.324732-0.357577': 0.020806, Hystrix: 0.362092) '0.345288-0.378684': 0.149148, (Ctenodactylus: 0.355197, Laonastes: 0.355197) '0.336957-0.375383': 0.156042) '0.496069-0.523894': 0.073537, (((Anomalurus: 0.442160, Pedetes: 0.442160) '0.421788-0.460763': 0.096054, (((((Cricetus: 0.186165, Mus: 0.186165) '0.170811-0.203546': 0.019146, Petromyscus: 0.205311) '0.188935-0.223659': 0.013732, Calomyscidae: 0.219043) '0.199352-0.241543': 0.133713, Spalax: 0.352755) '0.336821-0.37039': 0.089110, Jaculus: 0.441866) '0.42487-0.458033': 0.096347) '0.523575-0.549316': 0.014510, (Geomyidae: 0.231810, Dipodomys: 0.231810) '0.205513-0.257594': 0.320912) '0.538236-0.56278': 0.032054) '0.57033-0.593445': 0.005640, ((Aplodontia: 0.420298, Sciuridae: 0.420298) '0.398612-0.44236': 0.122980, Gliridae: 0.543278) '0.52739-0.557084': 0.047138) '0.576442-0.599839': 0.062825) '0.639127-0.666248': 0.026816) '0.665165-0.692567': 0.055972) '0.71776-0.752774': 0.053120) '0.76639-0.81001': 0.860685) '1.55852-1.71845': 0.377356, (Ornithorhynchus: 0.329492, Tachyglossus: 0.329492) '0.226745-0.453677': 1.697698) '1.90873-2.10021': 1.099386, ((Gallus: 0.823778, Taeniopygia: 0.823778) '0.7124-0.873984': 1.865427, Anolis: 2.689205) '2.566-2.82159': 0.437372) '3.08135-3.17937': 0.605614, Xenopus: 3.732190) '3.62278-3.79626': 0.474440, Danio: 4.206631) '4.15973-4.2543': 0.013211;

B. Independent rates

(((((((((Didelphis: 0.162182, Monodelphis: 0.162182) '0.142418-0.180703': 0.040159, Glironia: 0.202342) '0.181727-0.222942': 0.022009, Caluromys: 0.224351) '0.203586-0.24506': 0.296808, (Dromiciops: 0.434939, ((Notoryctes: 0.403959, (((Isoodon: 0.085436, Echymipera: 0.085436) '0.0725905-0.100134': 0.095354, Macrotis: 0.180790) '0.161571-0.203375': 0.205609, (Dasyurus: 0.188230, Myrmecobius: 0.188230) '0.164519-0.214499':

0.198170) '0.366373-0.408639': 0.017559) '0.383115-0.426606': 0.009182, ((((Acrobates: 0.269271, ((Petaurus:
0.219300, Pseudochirops: 0.219300) '0.200811-0.238115': 0.023603, Tarsipes: 0.242903) '0.225235-0.261939':
0.026368) '0.251052-0.287434': 0.034052, ((Macropus: 0.116737, Aepyprymnus: 0.116737) '0.0999019-
0.134109': 0.085479, Hypsiprymnodon: 0.202217) '0.182245-0.221805': 0.101106) '0.28541-0.322873':
0.008956, (Cercartetus: 0.285265, Trichosurus: 0.285265) '0.26355-0.307465': 0.027015) '0.294133-0.332602':
0.033896, Phascolarctos: 0.346176) '0.326862-0.36754': 0.066966) '0.392519-0.435623': 0.021798) '0.412044-
0.45833': 0.086219) '0.49155-0.548379': 0.049239, (Caenolestes: 0.080542, Rhyncholestes: 0.080542)
'0.0667293-0.0956154': 0.489856) '0.540815-0.607765': 1.043923, (((((Echinops: 0.548342, (Amblysomus:
0.083151, Chrysochloris: 0.083151) '0.0705066-0.09819': 0.465191) '0.520226-0.575598': 0.059745,
(Elephantulus: 0.424880, Rhynchocyon: 0.424880) '0.39247-0.461523': 0.183208) '0.586692-0.637201':
0.028720, (Heterohyrax: 0.033925, Procavia: 0.033925) '0.0272371-0.0418031': 0.602883) '0.613219-0.665616':
0.134134, (Dasypus: 0.456646, ((Choloepus: 0.151114, Bradypus: 0.151114) '0.131288-0.168544': 0.243034,
Cyclopes: 0.394148) '0.360957-0.424744': 0.062499) '0.426782-0.488317': 0.314295) '0.747005-0.795826':
0.014163, ((((Tragulus: 0.635864, (((((Prionodon: 0.175472, Felis: 0.175472) '0.156353-0.195414': 0.045161,
((Fossa: 0.135969, Suricata: 0.135969) '0.117649-0.155938': 0.071655, Genetta: 0.207623) '0.187702-0.224589':
0.013010) '0.201855-0.236987': 0.053769, Nandinia: 0.274403) '0.256945-0.28775': 0.120654, ((Ailurus:
0.225240, Procyon: 0.225240) '0.208587-0.241978': 0.020039, Mephitis: 0.245279) '0.230181-0.259461':
0.149778) '0.374667-0.415641': 0.211828, (Manis_pentadactyla: 0.155959, Manis_tricuspis: 0.155959)
'0.130056-0.182177': 0.450926) '0.590058-0.627649': 0.028980) '0.618819-0.65538': 0.005067,
(((((Craseonycteris: 0.355095, Megaderma: 0.355095) '0.328508-0.385016': 0.042538, Rhinopoma: 0.397634)
'0.374257-0.421906': 0.020695, (Hipposideros: 0.324410, Rhinolophus: 0.324410) '0.296559-0.345501':
0.093919) '0.396253-0.441135': 0.051880, Nyctimene: 0.470209) '0.446228-0.49499': 0.031162,
(((((Miniopterus: 0.333223, Myotis: 0.333223) '0.308422-0.357754': 0.036226, Tadarida: 0.369448) '0.346975-
0.392862': 0.016526, Natalus: 0.385975) '0.364195-0.408428': 0.023195, Myzopoda: 0.409170) '0.389049-
0.43308': 0.011931, ((Emballonuridae: 0.378698, Nycteris: 0.378698) '0.35481-0.403939': 0.038333,
((((Furipterus: 0.291203, Noctilio: 0.291203) '0.267945-0.316977': 0.039118, (Pteronotus: 0.286099, Artibeus:
0.286099) '0.264456-0.310657': 0.044222) '0.310243-0.353084': 0.007316, Thyroptera: 0.337637) '0.317249-
0.360691': 0.026342, Mystacina: 0.363979) '0.343508-0.388233': 0.053052) '0.396928-0.440143': 0.004071)
'0.400532-0.444139': 0.080270) '0.478251-0.526863': 0.139560) '0.623866-0.65981': 0.018769, ((((Erinaceus:
0.312781, Podogymnura: 0.312781) '0.280237-0.345667': 0.240992, Sorex: 0.553773) '0.534919-0.573529':
0.043791, Talpa: 0.597564) '0.580427-0.614018': 0.010574, Solenodon: 0.608138) '0.592151-0.625942':
0.051563) '0.643003-0.677085': 0.069088, ((((Cynocephalus: 0.056233, Galeopterus: 0.056233) '0.046307-
0.0682817': 0.603795, ((Callithrix: 0.562702, Tarsius: 0.562702) '0.542208-0.580469': 0.037322, ((((Microcebus:
0.168558, Propithecus: 0.168558) '0.147976-0.19421': 0.012556, Lemur: 0.181114) '0.159769-0.206975':
0.167842, Daubentonia: 0.348956) '0.323829-0.382507': 0.038350, (Otolemur: 0.153899, Nycticebus: 0.153899)
'0.13297-0.174303': 0.233408) '0.367925-0.417031': 0.212717) '0.580132-0.615322': 0.060004) '0.643756-
0.676391': 0.009745, (Ptilocercus: 0.434981, Tupaia: 0.434981) '0.395089-0.481182': 0.234792) '0.653188-
0.684872': 0.008385, ((Leporidae: 0.407194, Ochotona: 0.407194) '0.373028-0.440228': 0.249599,
((((((((Abrocoma: 0.234932, ((Capromys: 0.158317, (Myocastor: 0.123160, Hoplomys: 0.123160) '0.108858-
0.137691': 0.035157) '0.144191-0.172195': 0.061149, (Ctenomys: 0.176188, Octodontomys: 0.176188)
'0.157943-0.194145': 0.043279) '0.2054-0.233987': 0.015465) '0.220537-0.249673': 0.096844, (Chinchilla:
0.228877, Dinomys: 0.228877) '0.203315-0.253197': 0.102899) '0.31488-0.348531': 0.023564, ((Agouti:
0.249254, (Cavia: 0.228806, Dasyprocta: 0.228806) '0.209053-0.251101': 0.020449) '0.228588-0.271078':
0.090173, Erethizon: 0.339427) '0.321471-0.357429': 0.015912) '0.338237-0.37292': 0.057433, (Heterocephalus:
0.365290, (Petromus: 0.228959, Thryonomys: 0.228959) '0.207716-0.251752': 0.136331) '0.344601-0.385172':
0.047482) '0.395369-0.429898': 0.023061, Hystrix: 0.435833) '0.418098-0.453545': 0.107944, (Ctenodactylus:
0.387339, Laonastes: 0.387339) '0.354658-0.414559': 0.156438) '0.528443-0.558475': 0.056942, (((Anomalurus:
0.429335, Pedetes: 0.429335) '0.397619-0.46195': 0.125025, (((((Cricetus: 0.236511, Mus: 0.236511) '0.217126-
0.258644': 0.028527, Petromyscus: 0.265038) '0.244192-0.285965': 0.016879, Calomyscidae: 0.281917)
'0.260755-0.307024': 0.119788, Spalax: 0.401705) '0.381926-0.420746': 0.080766, Jaculus: 0.482471) '0.463375-
0.501216': 0.071889) '0.541164-0.568376': 0.016479, (Geomyidae: 0.288809, Dipodomys: 0.288809) '0.2557-
0.325349': 0.282030) '0.55934-0.583935': 0.029879) '0.590733-0.611302': 0.006095, ((Aplodontia: 0.398929,
Sciuridae: 0.398929) '0.359291-0.431339': 0.150695, Gliridae: 0.549624) '0.529004-0.567057': 0.057190)
'0.596417-0.616742': 0.049979) '0.641905-0.67089': 0.021365) '0.663161-0.693171': 0.050631) '0.710804-
0.746997': 0.056316) '0.762958-0.808543': 0.829216) '1.50232-1.70687': 0.370784, (Ornithorhynchus: 0.218738,
Tachyglossus: 0.218738) '0.175533-0.261434': 1.766367) '1.85438-2.086': 1.144911, ((Gallus: 0.705245,
Taeniopygia: 0.705245) '0.655381-0.788153': 1.903845, Anolis: 2.609091) '2.53931-2.72842': 0.520926)

'3.08141-3.18422': 0.614747, Xenopus: 3.744764) '3.64952-3.80426': 0.471938, Danio: 4.216701) '4.16195, 4.25554': 0.013211;

dR40$_{Springer}$ trees with key calibration bounds from Springer et al. (2017)

Lorisiformes 38.0-56.0 Ma, Lagomorpha 53.7-61.6 Ma, Emballonuroidea 47.8-59.2 Ma and Erinaceidae-Soricidae 61.6-83.8 Ma. This calibration scheme also incorporates Springer et al.'s [2] maximum bounds for the basal crown divergences of Chiroptera and Primates, and the three deepest rodent clades, with each pushed back to 66 Ma, as well as the maximum bound for Haplorhini pushed back from 57 Ma to 59.2 Ma.

A. Autocorrelated rates

(((((((((Didelphis: 0.263862, Monodelphis: 0.263862) '0.231897-0.291019': 0.071798, Glironia: 0.335660) '0.29754-0.372125': 0.036751, Caluromys: 0.372411) '0.332083-0.411443': 0.374738, (Dromiciops: 0.639795, ((Notoryctes: 0.597964, (((Isoodon: 0.113728, Echymipera: 0.113728) '0.0972798-0.131004': 0.137818, Macrotis: 0.251546) '0.219232-0.282593': 0.320205, (Dasyurus: 0.271052, Myrmecobius: 0.271052) '0.239958-0.303337': 0.300699) '0.521955-0.604195': 0.026213) '0.546205-0.628034': 0.012721, ((((Acrobates: 0.410125, ((Petaurus: 0.324942, Pseudochirops: 0.324942) '0.294126-0.352851': 0.042395, Tarsipes: 0.367337) '0.333713-0.394821': 0.042788) '0.373597-0.438846': 0.055841, ((Macropus: 0.170405, Aepyprymnus: 0.170405) '0.1491-0.18921': 0.124399, Hypsiprymnodon: 0.294804) '0.264036-0.318059': 0.171161) '0.425183-0.493304': 0.014386, (Cercartetus: 0.438456, Trichosurus: 0.438456) '0.401316-0.466696': 0.041896) '0.43907-0.508449': 0.047566, Phascolarctos: 0.527918) '0.48203-0.557779': 0.082768) '0.55906-0.64044': 0.029109) '0.585132-0.670302': 0.107354) '0.689549-0.778307': 0.063524, (Caenolestes: 0.138971, Rhyncholestes: 0.138971) '0.114227-0.161417': 0.671702) '0.753331-0.844651': 0.882186, (((((Echinops: 0.611423, (Amblysomus: 0.124347, Chrysochloris: 0.124347) '0.105054-0.14542': 0.487076) '0.590346-0.635477': 0.055685, (Elephantulus: 0.472793, Rhynchocyon: 0.472793) '0.444826-0.501916': 0.194315) '0.647533-0.690352': 0.027867, (Heterohyrax: 0.041177, Procavia: 0.041177) '0.032015-0.0515909': 0.653798) '0.674879-0.717814': 0.165437, (Dasypus: 0.608603, ((Choloepus: 0.217962, Bradypus: 0.217962) '0.191974-0.250811': 0.305604, Cyclopes: 0.523566) '0.493789-0.562402': 0.085037) '0.583382-0.646175': 0.251809) '0.839396-0.882666': 0.016517, ((((Tragulus: 0.729930, (((((Prionodon: 0.255863, Felis: 0.255863) '0.231047-0.281658': 0.058346, ((Fossa: 0.197965, Suricata: 0.197965) '0.177911-0.219272': 0.095671, Genetta: 0.293636) '0.270637-0.319415': 0.020573) '0.289624-0.340501': 0.068142, Nandinia: 0.382351) '0.354815-0.409781': 0.133969, ((Ailurus: 0.283180, Procyon: 0.283180) '0.256817-0.307627': 0.027326, Mephitis: 0.310507) '0.284293-0.335877': 0.205813) '0.489973-0.541897': 0.186586, (Manis_pentadactyla: 0.260502, Manis_tricuspis: 0.260502) '0.225074-0.297216': 0.442404) '0.683854-0.721328': 0.027025) '0.713634-0.746191': 0.006722, (((((Craseonycteris: 0.450266, Megaderma: 0.450266) '0.420722-0.477445': 0.058874, Rhinopoma: 0.509139) '0.484296-0.531744': 0.029475, (Hipposideros: 0.390152, Rhinolophus: 0.390152) '0.358248-0.417914': 0.148463) '0.51554-0.559976': 0.060962, Nyctimene: 0.599577) '0.578626-0.616638': 0.036889, (((((Miniopterus: 0.412797, Myotis: 0.412797) '0.393056-0.434081': 0.045683, Tadarida: 0.458480) '0.439921-0.477179': 0.021783, Natalus: 0.480263) '0.463149-0.497462': 0.033359, Myzopoda: 0.513623) '0.49676-0.530088': 0.016124, ((Emballonuridae: 0.480491, Nycteris: 0.480491) '0.468341-0.495482': 0.043519, ((((Furipterus: 0.362789, Noctilio: 0.362789) '0.340645-0.383274': 0.043009, (Pteronotus: 0.359331, Artibeus: 0.359331) '0.33713-0.379197': 0.046468) '0.38699-0.424653': 0.009233, Thyroptera: 0.415031) '0.396609-0.434024': 0.036443, Mystacina: 0.451474) '0.433985-0.469749': 0.072536) '0.509236-0.539554': 0.005736) '0.514706-0.545453': 0.106720) '0.618054-0.65235': 0.100186) '0.721152-0.752479': 0.017553, ((((Erinaceus: 0.295210, Podogymnura: 0.295210) '0.269201-0.321617': 0.317732, Sorex: 0.612942) '0.597226-0.630036': 0.050158, Talpa: 0.663101) '0.64826-0.680607': 0.015140, Solenodon: 0.678240) '0.662421-0.695479': 0.075965) '0.739625-0.769813': 0.066586, ((((Cynocephalus: 0.116744, Galeopterus: 0.116744) '0.0947543-0.138723': 0.622824, ((Callithrix: 0.628363, Tarsius: 0.628363) '0.605036-0.650534': 0.048034, ((((Microcebus: 0.287167, Propithecus: 0.287167) '0.264767-0.310171': 0.021302, Lemur: 0.308468) '0.285379-0.332321': 0.182709, Daubentonia: 0.491178) '0.467191-0.514143': 0.051841, (Otolemur: 0.232139, Nycticebus: 0.232139) '0.206388-0.256962': 0.310879) '0.521685-0.562031': 0.133378) '0.66052-0.694119': 0.063171) '0.724707-

0.754805': 0.011470, (Ptilocercus: 0.517827, Tupaia: 0.517827) '0.487353-0.543791': 0.233210) '0.736593-0.765587': 0.009704, ((Leporidae: 0.522786, Ochotona: 0.522786) '0.503016-0.536756': 0.210103, ((((((((Abrocoma: 0.172036, ((Capromys: 0.108201, (Myocastor: 0.085111, Hoplomys: 0.085111) '0.0760419-0.0939967': 0.023090) '0.0978562-0.118409': 0.050465, (Ctenomys: 0.130840, Octodontomys: 0.130840) '0.119623-0.142759': 0.027827) '0.146306-0.171821': 0.013369) '0.159056-0.185627': 0.112786, (Chinchilla: 0.204566, Dinomys: 0.204566) '0.188946-0.220749': 0.080256) '0.27-0.301828': 0.026487, ((Agouti: 0.213018, (Cavia: 0.199185, Dasyprocta: 0.199185) '0.184621-0.214976': 0.013833) '0.198392-0.229227': 0.086151, Erethizon: 0.299169) '0.28336-0.317528': 0.012140) '0.295817-0.32976': 0.064916, (Heterocephalus: 0.328303, (Petromus: 0.196935, Thryonomys: 0.196935) '0.17947-0.216059': 0.131368) '0.310191-0.349974': 0.047922) '0.359183-0.396617': 0.023972, Hystrix: 0.400198) '0.38279-0.420046': 0.170392, (Ctenodactylus: 0.397690, Laonastes: 0.397690) '0.372609-0.419105': 0.172900) '0.555392-0.584008': 0.083106, (((Anomalurus: 0.495307, Pedetes: 0.495307) '0.472805-0.517538': 0.107044, (((((Cricetus: 0.211504, Mus: 0.211504) '0.192465-0.230519': 0.021731, Petromyscus: 0.233236) '0.212792-0.252759': 0.015725, Calomyscidae: 0.248960) '0.225609-0.273455': 0.148225, Spalax: 0.397185) '0.375342-0.417367': 0.098683, Jaculus: 0.495868) '0.47531-0.514534': 0.106483) '0.587553-0.614542': 0.016053, (Geomyidae: 0.259371, Dipodomys: 0.259371) '0.232191-0.287208': 0.359033) '0.604333-0.630139': 0.035291) '0.642316-0.662269': 0.006109, ((Aplodontia: 0.471125, Sciuridae: 0.471125) '0.445555-0.494635': 0.136834, Gliridae: 0.607959) '0.593608-0.620341': 0.051845) '0.649129-0.668209': 0.073084) '0.719447-0.745392': 0.027852) '0.74724-0.774269': 0.060050) '0.80505-0.836845': 0.056138) '0.857631-0.897541': 0.815930) '1.63498-1.73801': 0.359414, (Ornithorhynchus: 0.328039, Tachyglossus: 0.328039) '0.222702-0.470584': 1.724234) '1.97131-2.10786': 1.073830, ((Gallus: 0.828619, Taeniopygia: 0.828619) '0.739462-0.871603': 1.856984, Anolis: 2.685603) '2.56021-2.82414': 0.440500) '3.07834-3.17955': 0.609133, Xenopus: 3.735236) '3.62909-3.80081': 0.471734, Danio: 4.206970) '4.1601, 4.25459': 0.013211;

B. Independent rates

(((((((((Didelphis: 0.178344, Monodelphis: 0.178344) '0.15683-0.199969': 0.043579, Glironia: 0.221924) '0.197786-0.243909': 0.024426, Caluromys: 0.246350) '0.221381-0.268876': 0.303671, (Dromiciops: 0.469200, ((Notoryctes: 0.436424, (((Isoodon: 0.093845, Echymipera: 0.093845) '0.0796056-0.109478': 0.104122, Macrotis: 0.197967) '0.176786-0.222325': 0.219228, (Dasyurus: 0.205382, Myrmecobius: 0.205382) '0.180115-0.23384': 0.211813) '0.395755-0.438984': 0.019229) '0.415818-0.457426': 0.009977, ((((Acrobates: 0.290105, ((Petaurus: 0.234934, Pseudochirops: 0.234934) '0.21647-0.253675': 0.026185, Tarsipes: 0.261119) '0.243674-0.279286': 0.028986) '0.272972-0.309026': 0.037937, ((Macropus: 0.126858, Aepyprymnus: 0.126858) '0.109252-0.144067': 0.092675, Hypsiprymnodon: 0.219533) '0.199071-0.238992': 0.108509) '0.31109-0.346081': 0.009923, (Cercartetus: 0.309059, Trichosurus: 0.309059) '0.287435-0.329956': 0.028906) '0.320712-0.35624': 0.037451, Phascolarctos: 0.375416) '0.356125-0.394022': 0.070985) '0.426046-0.467371': 0.022799) '0.448702-0.491027': 0.080822) '0.531852-0.575076': 0.056818, (Caenolestes: 0.088359, Rhyncholestes: 0.088359) '0.0733345-0.105227': 0.518480) '0.58347-0.634737': 1.068789, (((((Echinops: 0.607726, (Amblysomus: 0.092321, Chrysochloris: 0.092321) '0.0779976-0.110513': 0.515405) '0.575296-0.638692': 0.067129, (Elephantulus: 0.473206, Rhynchocyon: 0.473206) '0.435756-0.51355': 0.201650) '0.647286-0.70613': 0.031460, (Heterohyrax: 0.037435, Procavia: 0.037435) '0.0301662-0.0456654': 0.668881) '0.678266-0.7379': 0.148591, (Dasypus: 0.499783, ((Choloepus: 0.159312, Bradypus: 0.159312) '0.140339-0.179513': 0.272154, Cyclopes: 0.431466) '0.398462-0.4653': 0.068317) '0.471968-0.535266': 0.355124) '0.828537-0.885014': 0.016139, ((((Tragulus: 0.710003, (((((Prionodon: 0.184345, Felis: 0.184345) '0.164452-0.20388': 0.046918, ((Fossa: 0.142026, Suricata: 0.142026) '0.124182-0.162194': 0.075497, Genetta: 0.217523) '0.199012-0.235006': 0.013740) '0.213334-0.248193': 0.050217, Nandinia: 0.281479) '0.267399-0.296534': 0.126450, ((Ailurus: 0.231728, Procyon: 0.231728) '0.213855-0.251312': 0.021675, Mephitis: 0.253403) '0.238812-0.2718': 0.154526) '0.38846-0.434319': 0.269573, (Manis_pentadactyla: 0.172451, Manis_tricuspis: 0.172451) '0.142235-0.204713': 0.505051) '0.656173-0.701928': 0.032500) '0.68998-0.733064': 0.005723, (((((Craseonycteris: 0.398822, Megaderma: 0.398822) '0.37041-0.428961': 0.047254, Rhinopoma: 0.446076) '0.419891-0.472813': 0.023062, (Hipposideros: 0.342804, Rhinolophus: 0.342804) '0.3266-0.363988': 0.126333) '0.444424-0.494798': 0.069212, Nyctimene: 0.538349) '0.512911-0.561548': 0.038196, (((((Miniopterus: 0.395887, Myotis: 0.395887) '0.36958-0.422548': 0.040797, Tadarida: 0.436683) '0.413305-0.459312': 0.018829, Natalus: 0.455512) '0.433229-0.479116': 0.026648, Myzopoda: 0.482160) '0.460873-0.503746': 0.014240, ((Emballonuridae: 0.461895, Nycteris: 0.461895) '0.439256-0.4815': 0.030562, ((((Furipterus: 0.343584, Noctilio: 0.343584) '0.314846-0.373931': 0.044978, (Pteronotus: 0.338729, Artibeus: 0.338729) '0.31357-0.366048': 0.049834) '0.366279-0.414954': 0.008531, Thyroptera: 0.397094) '0.376054-0.422419': 0.030984, Mystacina: 0.428078) '0.405627-

0.452488': 0.064379) '0.471281-0.513222': 0.003942) '0.475498-0.517145': 0.080145) '0.553354-0.598528': 0.139181) '0.695966-0.738093': 0.020295, ((((Erinaceus: 0.347446, Podogymnura: 0.347446) '0.313382-0.385408': 0.272778, Sorex: 0.620223) '0.604022-0.639389': 0.046758, Talpa: 0.666981) '0.649818-0.687186': 0.011848, Solenodon: 0.678829) '0.661966-0.699526': 0.057191) '0.717745-0.757204': 0.074899, ((((Cynocephalus: 0.062106, Galeopterus: 0.062106) '0.0509243-0.0758048': 0.673268, ((Callithrix: 0.606839, Tarsius: 0.606839) '0.587955-0.62524': 0.044897, ((((Microcebus: 0.192952, Propithecus: 0.192952) '0.165146-0.224701': 0.014081, Lemur: 0.207033) '0.178777-0.239168': 0.202062, Daubentonia: 0.409095) '0.37191-0.450403': 0.044584, (Otolemur: 0.237902, Nycticebus: 0.237902) '0.209032-0.268043': 0.215778) '0.418105-0.49282': 0.198056) '0.630464-0.664836': 0.083638) '0.717116-0.754775': 0.011772, (Ptilocercus: 0.484361, Tupaia: 0.484361) '0.439899-0.539521': 0.262785) '0.730081-0.766239': 0.009998, ((Leporidae: 0.537550, Ochotona: 0.537550) '0.527696-0.548681': 0.196055, ((((((((Abrocoma: 0.258961, ((Capromys: 0.174140, (Myocastor: 0.135252, Hoplomys: 0.135252) '0.120071-0.150798': 0.038888) '0.158526-0.190494': 0.067679, (Ctenomys: 0.194769, Octodontomys: 0.194769) '0.17517-0.216214': 0.047051) '0.225796-0.25955': 0.017141) '0.242047-0.276805': 0.107987, (Chinchilla: 0.252364, Dinomys: 0.252364) '0.223615-0.28172': 0.114583) '0.345344-0.385756': 0.025837, ((Agouti: 0.275300, (Cavia: 0.252685, Dasyprocta: 0.252685) '0.227722-0.278179': 0.022615) '0.250733-0.300187': 0.100065, Erethizon: 0.375365) '0.352216-0.39547': 0.017420) '0.370967-0.412074': 0.062132, (Heterocephalus: 0.402421, (Petromus: 0.251968, Thryonomys: 0.251968) '0.227946-0.27775': 0.150453) '0.37984-0.424333': 0.052496) '0.434416-0.474608': 0.026249, Hystrix: 0.481166) '0.460145-0.501423': 0.122830, (Ctenodactylus: 0.429645, Laonastes: 0.429645) '0.394139-0.462815': 0.174350) '0.587068-0.62232': 0.065915, (((Anomalurus: 0.481227, Pedetes: 0.481227) '0.444812-0.518704': 0.139883, (((((Cricetus: 0.263863, Mus: 0.263863) '0.239434-0.289447': 0.031875, Petromyscus: 0.295739) '0.270894-0.322271': 0.019910, Calomyscidae: 0.315649) '0.287233-0.342981': 0.133971, Spalax: 0.449619) '0.427735-0.471772': 0.091145, Jaculus: 0.540764) '0.520907-0.560705': 0.080345) '0.606389-0.639892': 0.019293, (Geomyidae: 0.325141, Dipodomys: 0.325141) '0.285963-0.362729': 0.315261) '0.626024-0.65748': 0.029509) '0.657667-0.68651': 0.005645, ((Aplodontia: 0.443162, Sciuridae: 0.443162) '0.398049-0.480492': 0.166873, Gliridae: 0.610035) '0.586458-0.63243': 0.065521) '0.662816-0.69191': 0.058049) '0.717695-0.751513': 0.023539) '0.740815-0.775843': 0.053775) '0.791638-0.832859': 0.060127) '0.846556-0.899072': 0.804581) '1.59335-1.73248': 0.367888, (Ornithorhynchus: 0.237522, Tachyglossus: 0.237522) '0.193868-0.282129': 1.805994) '1.94962-2.10522': 1.093163, ((Gallus: 0.741885, Taeniopygia: 0.741885) '0.663296-0.847784': 1.863681, Anolis: 2.605566) '2.53519-2.71975': 0.531113) '3.09245-3.20474': 0.617878, Xenopus: 3.754557) '3.6709-3.8126': 0.463666, Danio: 4.218223) '4.16249-4.25574': 0.013211;

dR40 trees with key maximum bounds from Springer et al. (2017)

This calibration scheme follows the dR40 calibration scheme, except also incorporating Springer et al.'s [2] maximum bounds for the basal crown divergences of Chiroptera and Primates, and for the three deepest rodent clades, with each pushed back to 66 Ma, as well as for the Haplorhini maximum bound being pushed back from 57 Ma to 59.2 Ma.

A. Autocorrelated rates

(((((((((Didelphis: 0.261826, Monodelphis: 0.261826) '0.223102-0.28723': 0.070319, Glironia: 0.332145) '0.285629-0.362608': 0.034992, Caluromys: 0.367137) '0.319061-0.400006': 0.351479, (Dromiciops: 0.613194, ((Notoryctes: 0.572824, (((Isoodon: 0.107739, Echymipera: 0.107739) '0.0926708-0.126589': 0.131671, Macrotis: 0.239410) '0.211861-0.269908': 0.307861, (Dasyurus: 0.258943, Myrmecobius: 0.258943) '0.228698-0.288003': 0.288328) '0.5141-0.586808': 0.025553) '0.538478-0.611538': 0.012417, ((((Acrobates: 0.391868, ((Petaurus: 0.310139, Pseudochirops: 0.310139) '0.284587-0.332466': 0.040629, Tarsipes: 0.350768) '0.324708-0.373392': 0.041100) '0.364509-0.416148': 0.053256, ((Macropus: 0.164776, Aepyprymnus: 0.164776) '0.145001-0.181548': 0.117418, Hypsiprymnodon: 0.282194) '0.258096-0.305917': 0.162930) '0.41687-0.472218': 0.013966, (Cercartetus: 0.419236, Trichosurus: 0.419236) '0.392071-0.447671': 0.039853) '0.430611-0.487353': 0.046421, Phascolarctos: 0.505510) '0.474472-0.538981': 0.079731) '0.550736-0.623828': 0.027953) '0.579501-0.654204': 0.105422) '0.679097-0.764474': 0.063146, (Caenolestes: 0.137220, Rhyncholestes: 0.137220) '0.10964-0.161356': 0.644542) '0.735903-0.831854': 0.877718, (((((Echinops: 0.554682, (Amblysomus: 0.112619,

Chrysochloris: 0.112619) '0.094648-0.132556': 0.442064) '0.531538-0.586276': 0.051169, (Elephantulus: 0.428132, Rhynchocyon: 0.428132) '0.401808-0.458772': 0.177719) '0.584559-0.634648': 0.025763, (Heterohyrax: 0.037544, Procavia: 0.037544) '0.0291674-0.0472291': 0.594069) '0.608579-0.659656': 0.154692, (Dasypus: 0.555046, ((Choloepus: 0.199461, Bradypus: 0.199461) '0.17696-0.226928': 0.277492, Cyclopes: 0.476954) '0.44904-0.515479': 0.078092) '0.52755-0.593207': 0.231259) '0.763495-0.811325': 0.015260, ((((Tragulus: 0.661249, (((((Prionodon: 0.232783, Felis: 0.232783) '0.210299-0.255123': 0.052692, ((Fossa: 0.179362, Suricata: 0.179362) '0.161359-0.19743': 0.087052, Genetta: 0.266414) '0.244773-0.28768': 0.019061) '0.261223-0.307193': 0.062162, Nandinia: 0.347637) '0.321099-0.369842': 0.120835, ((Ailurus: 0.258151, Procyon: 0.258151) '0.234782-0.280842': 0.024328, Mephitis: 0.282479) '0.259379-0.305851': 0.185993) '0.444243-0.490987': 0.167968, (Manis_pentadactyla: 0.234723, Manis_tricuspis: 0.234723) '0.196254-0.269353': 0.401716) '0.618051-0.657496': 0.024809) '0.643414-0.680686': 0.006090, (((((Craseonycteris: 0.404801, Megaderma: 0.404801) '0.380693-0.429043': 0.052722, Rhinopoma: 0.457523) '0.436013-0.478946': 0.026767, (Hipposideros: 0.353422, Rhinolophus: 0.353422) '0.334525-0.37528': 0.130868) '0.463357-0.504694': 0.054284, Nyctimene: 0.538574) '0.517025-0.5577': 0.033317, (((((Miniopterus: 0.365122, Myotis: 0.365122) '0.345475-0.389921': 0.041459, Tadarida: 0.406581) '0.386055-0.429374': 0.019509, Natalus: 0.426090) '0.405209-0.447933': 0.029963, Myzopoda: 0.456053) '0.435264-0.478717': 0.014574, ((Emballonuridae: 0.422238, Nycteris: 0.422238) '0.399591-0.445945': 0.042687, ((((Furipterus: 0.319129, Noctilio: 0.319129) '0.296657-0.346401': 0.039008, (Pteronotus: 0.316028, Artibeus: 0.316028) '0.295486-0.342777': 0.042109) '0.338968-0.384269': 0.008069, Thyroptera: 0.366207) '0.347333-0.39209': 0.032982, Mystacina: 0.399189) '0.379477-0.424485': 0.065736) '0.444392-0.487337': 0.005702) '0.449683-0.492688': 0.101263) '0.551203-0.59133': 0.095448) '0.65001-0.686117': 0.016574, ((((Erinaceus: 0.266156, Podogymnura: 0.266156) '0.242363-0.288863': 0.284401, Sorex: 0.550557) '0.53205-0.574588': 0.047717, Talpa: 0.598274) '0.581111-0.61992': 0.014366, Solenodon: 0.612639) '0.594165-0.633359': 0.071273) '0.667169-0.70215': 0.064302, ((((Cynocephalus: 0.105675, Galeopterus: 0.105675) '0.0837863-0.126327': 0.571697, ((Callithrix: 0.589636, Tarsius: 0.589636) '0.572445-0.605409': 0.039232, ((((Microcebus: 0.260441, Propithecus: 0.260441) '0.240116-0.281808': 0.019168, Lemur: 0.279609) '0.258319-0.301416': 0.165794, Daubentonia: 0.445403) '0.423181-0.467503': 0.046816, (Otolemur: 0.173025, Nycticebus: 0.173025) '0.153299-0.193079': 0.319194) '0.471799-0.512517': 0.136650) '0.6137-0.643656': 0.048503) '0.663242-0.69183': 0.008153, (Ptilocercus: 0.468755, Tupaia: 0.468755) '0.442146-0.494207': 0.216769) '0.671559-0.69973': 0.006901, ((Leporidae: 0.409673, Ochotona: 0.409673) '0.382158-0.436152': 0.253154, ((((((((Abrocoma: 0.157966, ((Capromys: 0.099527, (Myocastor: 0.078366, Hoplomys: 0.078366) '0.0698113-0.0870813': 0.021162) '0.0896765-0.109621': 0.046176, (Ctenomys: 0.120214, Octodontomys: 0.120214) '0.109419-0.131109': 0.025489) '0.133678-0.158212': 0.012263) '0.14525-0.171221': 0.102793, (Chinchilla: 0.186410, Dinomys: 0.186410) '0.172176-0.201676': 0.074349) '0.245389-0.276658': 0.023991, ((Agouti: 0.194143, (Cavia: 0.181505, Dasyprocta: 0.181505) '0.168078-0.196268': 0.012638) '0.180586-0.209151': 0.079350, Erethizon: 0.273493) '0.257752-0.289937': 0.011258) '0.269328-0.30136': 0.059086, (Heterocephalus: 0.300057, (Petromus: 0.179880, Thryonomys: 0.179880) '0.164939-0.195414': 0.120176) '0.283514-0.317014': 0.043780) '0.327464-0.360179': 0.020972, Hystrix: 0.364808) '0.348495-0.381799': 0.150400, (Ctenodactylus: 0.357781, Laonastes: 0.357781) '0.338734-0.376627': 0.157427) '0.501953-0.526999': 0.074557, (((Anomalurus: 0.445150, Pedetes: 0.445150) '0.425942-0.462898': 0.097123, (((((Cricetus: 0.187479, Mus: 0.187479) '0.171657-0.203552': 0.019252, Petromyscus: 0.206731) '0.189694-0.224278': 0.013738, Calomyscidae: 0.220469) '0.199318-0.241844': 0.134870, Spalax: 0.355339) '0.339021-0.372462': 0.089591, Jaculus: 0.444930) '0.429314-0.460366': 0.097343) '0.530432-0.551778': 0.014557, (Geomyidae: 0.232960, Dipodomys: 0.232960) '0.207955-0.259065': 0.323871) '0.54594-0.56523': 0.032935) '0.579668-0.596721': 0.006175, ((Aplodontia: 0.423703, Sciuridae: 0.423703) '0.401219-0.444495': 0.124566, Gliridae: 0.548269) '0.535024-0.559922': 0.047672) '0.585764-0.60357': 0.066887) '0.649125-0.675614': 0.029598) '0.678567-0.706413': 0.055789) '0.730918-0.766136': 0.053351) '0.78053-0.823623': 0.857915) '1.56803-1.72083': 0.375157, (Ornithorhynchus: 0.329626, Tachyglossus: 0.329626) '0.232049-0.459772': 1.705011) '1.91825-2.10405': 1.092261, ((Gallus: 0.821759, Taeniopygia: 0.821759) '0.711946-0.873098': 1.869078, Anolis: 2.690837) '2.56175-2.8295': 0.436061) '3.07966-3.18355': 0.604091, Xenopus: 3.730990) '3.61465-3.79778': 0.475511, Danio: 4.206501) '4.16012-4.25404': 0.013211;

B. Independent rates

(((((((((Didelphis: 0.163366, Monodelphis: 0.163366) '0.143332-0.182961': 0.040476, Glironia: 0.203842) '0.181332-0.225031': 0.022133, Caluromys: 0.225975) '0.203579-0.246926': 0.293314, (Dromiciops: 0.435013, ((Notoryctes: 0.403999, (((Isoodon: 0.086152, Echymipera: 0.086152) '0.0733559-0.0993837': 0.095444, Macrotis: 0.181595) '0.161649-0.205641': 0.204813, (Dasyurus: 0.188398, Myrmecobius: 0.188398) '0.165329-

0.215252': 0.198010) '0.364734-0.408541': 0.017591) '0.383086-0.426137': 0.009217, ((((Acrobates: 0.269940, ((Petaurus: 0.219883, Pseudochirops: 0.219883) '0.201241-0.238069': 0.023636, Tarsipes: 0.243519) '0.225883-0.261902': 0.026421) '0.251545-0.288588': 0.034042, ((Macropus: 0.117161, Aepyprymnus: 0.117161) '0.100395-0.134137': 0.086124, Hypsiprymnodon: 0.203285) '0.182403-0.223063': 0.100697) '0.285537-0.322181': 0.008975, (Cercartetus: 0.285890, Trichosurus: 0.285890) '0.26446-0.305304': 0.027067) '0.293976-0.331554': 0.033929, Phascolarctos: 0.346887) '0.32757-0.367253': 0.066330) '0.392594-0.435893': 0.021797) '0.412285-0.458442': 0.084276) '0.49077-0.54894': 0.048933, (Caenolestes: 0.080946, Rhyncholestes: 0.080946) '0.0672171-0.0957727': 0.487276) '0.540826-0.607847': 1.054173, (((((Echinops: 0.554696, (Amblysomus: 0.083857, Chrysochloris: 0.083857) '0.0702804-0.0998591': 0.470840) '0.526027-0.583426': 0.060549, (Elephantulus: 0.429881, Rhynchocyon: 0.429881) '0.398458-0.466898': 0.185364) '0.592081-0.646274': 0.028950, (Heterohyrax: 0.034195, Procavia: 0.034195) '0.0277417-0.0418803': 0.610000) '0.618531-0.675645': 0.134408, (Dasypus: 0.460107, ((Choloepus: 0.151824, Bradypus: 0.151824) '0.131515-0.170184': 0.245452, Cyclopes: 0.397276) '0.362746-0.428818': 0.062831) '0.429443-0.49334': 0.318496) '0.752372-0.808011': 0.014344, ((((Tragulus: 0.642599, (((((Prionodon: 0.176326, Felis: 0.176326) '0.156559-0.195851': 0.045560, ((Fossa: 0.136559, Suricata: 0.136559) '0.117918-0.156844': 0.072228, Genetta: 0.208787) '0.189179-0.225454': 0.013099) '0.202686-0.237941': 0.053347, Nandinia: 0.275234) '0.258219-0.288361': 0.120673, ((Ailurus: 0.226067, Procyon: 0.226067) '0.210642-0.241145': 0.020159, Mephitis: 0.246226) '0.232466-0.259597': 0.149681) '0.37563-0.415725': 0.217793, (Manis_pentadactyla: 0.156660, Manis_tricuspis: 0.156660) '0.129195-0.182111': 0.457040) '0.593485-0.638167': 0.028899) '0.621636-0.667315': 0.005093, (((((Craseonycteris: 0.355921, Megaderma: 0.355921) '0.329201-0.384158': 0.042709, Rhinopoma: 0.398630) '0.373787-0.424101': 0.020872, (Hipposideros: 0.324714, Rhinolophus: 0.324714) '0.298283-0.346096': 0.094788) '0.396873-0.444124': 0.052735, Nyctimene: 0.472237) '0.44724-0.499656': 0.031816, (((((Miniopterus: 0.335217, Myotis: 0.335217) '0.307843-0.362464': 0.036507, Tadarida: 0.371723) '0.34766-0.397122': 0.016636, Natalus: 0.388359) '0.365462-0.413599': 0.023322, Myzopoda: 0.411681) '0.389728-0.436721': 0.012054, ((Emballonuridae: 0.381113, Nycteris: 0.381113) '0.356355-0.407897': 0.038535, ((((Furipterus: 0.293398, Noctilio: 0.293398) '0.268323-0.320983': 0.039426, (Pteronotus: 0.288589, Artibeus: 0.288589) '0.264368-0.314904': 0.044236) '0.31045-0.356764': 0.007329, Thyroptera: 0.340154) '0.31809-0.364413': 0.026433, Mystacina: 0.366587) '0.344158-0.391698': 0.053061) '0.398428-0.443972': 0.004087) '0.402202-0.448049': 0.080319) '0.479705-0.532555': 0.143638) '0.626604-0.672082': 0.018964, ((((Erinaceus: 0.316510, Podogymnura: 0.316510) '0.282916-0.349946': 0.242943, Sorex: 0.559453) '0.539261-0.58177': 0.044382, Talpa: 0.603835) '0.582589-0.625565': 0.010734, Solenodon: 0.614569) '0.59434-0.636977': 0.052087) '0.645761-0.689947': 0.069733, ((((Cynocephalus: 0.056611, Galeopterus: 0.056611) '0.0463367-0.0681262': 0.612366, ((Callithrix: 0.575848, Tarsius: 0.575848) '0.548486-0.598602': 0.035708, ((((Microcebus: 0.168591, Propithecus: 0.168591) '0.147234-0.199265': 0.012580, Lemur: 0.181171) '0.159078-0.211366': 0.169568, Daubentonia: 0.350739) '0.324596-0.384039': 0.038820, (Otolemur: 0.155156, Nycticebus: 0.155156) '0.133526-0.176925': 0.234403) '0.368993-0.42108': 0.221996) '0.585832-0.633215': 0.057422) '0.649342-0.691041': 0.009536, (Ptilocercus: 0.439914, Tupaia: 0.439914) '0.399335-0.485441': 0.238599) '0.659193-0.700044': 0.008253, ((Leporidae: 0.410914, Ochotona: 0.410914) '0.374779-0.446214': 0.253069, ((((((((Abrocoma: 0.237037, ((Capromys: 0.159844, (Myocastor: 0.124393, Hoplomys: 0.124393) '0.109964-0.138233': 0.035452) '0.145737-0.174135': 0.061615, (Ctenomys: 0.177855, Octodontomys: 0.177855) '0.160484-0.196112': 0.043605) '0.206794-0.236439': 0.015577) '0.221471-0.252032': 0.097570, (Chinchilla: 0.230659, Dinomys: 0.230659) '0.205403-0.25788': 0.103947) '0.316665-0.352251': 0.023793, ((Agouti: 0.250786, (Cavia: 0.230151, Dasyprocta: 0.230151) '0.209728-0.251599': 0.020636) '0.230059-0.272068': 0.091591, Erethizon: 0.342378) '0.32333-0.360825': 0.016022) '0.340713-0.376168': 0.057835, (Heterocephalus: 0.368580, (Petromus: 0.231098, Thryonomys: 0.231098) '0.20985-0.254184': 0.137482) '0.3484-0.389388': 0.047654) '0.398361-0.434292': 0.023391, Hystrix: 0.439625) '0.420689-0.458943': 0.109244, (Ctenodactylus: 0.390554, Laonastes: 0.390554) '0.358807-0.418879': 0.158315) '0.530676-0.569334': 0.057615, (((Anomalurus: 0.433897, Pedetes: 0.433897) '0.401553-0.468915': 0.125653, (((((Cricetus: 0.238533, Mus: 0.238533) '0.217407-0.260551': 0.028778, Petromyscus: 0.267311) '0.244915-0.288855': 0.016912, Calomyscidae: 0.284224) '0.260001-0.309772': 0.121190, Spalax: 0.405414) '0.384183-0.427203': 0.081786, Jaculus: 0.487199) '0.466367-0.509614': 0.072351) '0.543667-0.580067': 0.016519, (Geomyidae: 0.291841, Dipodomys: 0.291841) '0.258578-0.330002': 0.284228) '0.560734-0.596189': 0.030416) '0.592008-0.625498': 0.006347, ((Aplodontia: 0.402320, Sciuridae: 0.402320) '0.363194-0.435585': 0.152518, Gliridae: 0.554838) '0.531781-0.578124': 0.057993) '0.598097-0.63155': 0.051152) '0.645623-0.685279': 0.022783) '0.667908-0.708861': 0.049622) '0.714987-0.76083': 0.056558) '0.768243-0.821779': 0.829448) '1.51493-1.7112': 0.369565, (Ornithorhynchus: 0.220539, Tachyglossus: 0.220539) '0.175269-0.264767': 1.771421) '1.86523-2.08896': 1.138328, ((Gallus: 0.707337, Taeniopygia:

0.707337) '0.655266-0.793324': 1.901489, Anolis: 2.608827) '2.53868-2.7254': 0.521461) '3.08712-3.18704': 0.615388, Xenopus: 3.745676) '3.65428-3.80485': 0.471298, Danio: 4.216974) '4.16204-4.25557': 0.013211;

128-taxon trees

These analyses restore taxa that are 10-30kg, but still <40 years maximum longevity. Including these additional moderately large mammals and their respective calibration priors (Additional file 2: Table S2) has a very small upwards impact on divergence estimates, which remain consistent with the soft explosive model. Relative to the average for the 122-taxon dr40trees, the midpoint for the primary placental interordinal diversification increases from 64.5 Ma to 65.3 Ma

A. Autocorrelated rates

(((((((((Didelphis: 0.217938, Monodelphis: 0.217938) '0.189-0.252': 0.057838, Glironia: 0.275776) '0.247-0.312': 0.018646, Caluromys: 0.294422) '0.261-0.333': 0.357596, (Dromiciops: 0.561700, ((Notoryctes: 0.540882, (((Isoodon: 0.100309, Echymipera: 0.100309) '0.085-0.120': 0.140383, Macrotis: 0.240692) '0.212-0.281': 0.287826, (Dasyurus: 0.250721, Myrmecobius: 0.250721) '0.214-0.301': 0.277797) '0.502-0.582': 0.012364) '0.514-0.593': 0.005280, ((((Acrobates: 0.371076, ((Petaurus: 0.306265, Pseudochirops: 0.306265) '0.286-0.341': 0.024042, Tarsipes: 0.330307) '0.310-0.366': 0.040769) '0.351-0.408': 0.040335, ((Macropus: 0.145159, Aepyprymnus: 0.145159) '0.125-0.170': 0.109298, Hypsiprymnodon: 0.254457) '0.236-0.283': 0.156954) '0.390-0.451': 0.009754, (Cercartetus: 0.387480, Trichosurus: 0.387480) '0.364-0.429': 0.033685) '0.400-0.461': 0.043940, (Phascolarctos: 0.274429, Vombatus: 0.274429) '0.246-0.313': 0.190676) '0.441-0.511': 0.081057) '0.519-0.596': 0.015538) '0.533-0.615': 0.090318) '0.618-0.710': 0.046761, (Caenolestes: 0.095444, Rhyncholestes: 0.095444) '0.074-0.125': 0.603335) '0.662-0.766': 1.018432, (((((Echinops: 0.546325, (Amblysomus: 0.087584, Chrysochloris: 0.087584) '0.065-0.107': 0.458740) '0.523-0.584': 0.043715, (Elephantulus: 0.391864, Rhynchocyon: 0.391864) '0.358-0.439': 0.198176) '0.573-0.621': 0.017981, (Heterohyrax: 0.030859, Procavia: 0.030859) '0.022-0.040': 0.577163) '0.590-0.638': 0.159508, (Dasypus: 0.507199, ((Choloepus: 0.168744, Bradypus: 0.168744) '0.147-0.197': 0.276559, (Cyclopes: 0.288827, Myrmecophaga: 0.288827) '0.252-0.324': 0.156477) '0.420-0.464': 0.061896) '0.476-0.530': 0.260329) '0.751-0.786': 0.005542, ((((((Moschus: 0.313777, Tragulus: 0.313777) '0.285-0.337': 0.198291, Pecari: 0.512069) '0.498-0.531': 0.142618, (((((Prionodon: 0.244682, Felis: 0.244682) '0.224-0.267': 0.030185, ((Fossa: 0.164963, Suricata: 0.164963) '0.144-0.186': 0.100718, Genetta: 0.265680) '0.247-0.286': 0.009187) '0.256-0.296': 0.054735, Nandinia: 0.329602) '0.312-0.353': 0.109972, ((Ailurus: 0.249086, (Procyon: 0.229851, Meles: 0.229851) '0.213-0.250': 0.019236) '0.234-0.268': 0.010287, Mephitis: 0.259373) '0.245-0.278': 0.180201) '0.419-0.459': 0.199976, (Manis_pentadactyla: 0.176998, Manis_tricuspis: 0.176998) '0.138-0.221': 0.462552) '0.624-0.655': 0.015137) '0.640-0.669': 0.000731, (((((Craseonycteris: 0.392016, Megaderma: 0.392016) '0.369-0.415': 0.049520, Rhinopoma: 0.441535) '0.426-0.458': 0.015038, (Hipposideros: 0.366741, Rhinolophus: 0.366741) '0.347-0.386': 0.089832) '0.442-0.473': 0.061818, Nyctimene: 0.518391) '0.505-0.532': 0.021839, (((((Miniopterus: 0.378393, Myotis: 0.378393) '0.361-0.396': 0.026621, Tadarida: 0.405014) '0.390-0.421': 0.015510, Natalus: 0.420523) '0.407-0.435': 0.020096, Myzopoda: 0.440619) '0.427-0.455': 0.014639, ((Emballonuridae: 0.417830, Nycteris: 0.417830) '0.401-0.435': 0.035827, ((((Furipterus: 0.321873, Noctilio: 0.321873) '0.303-0.339': 0.040345, (Pteronotus: 0.319471, Artibeus: 0.319471) '0.301-0.335': 0.042747) '0.348-0.376': 0.005010, Thyroptera: 0.367228) '0.353-0.381': 0.028446, Mystacina: 0.395674) '0.382-0.410': 0.057983) '0.441-0.467': 0.001601) '0.443-0.468': 0.084972) '0.525-0.554': 0.115187) '0.641-0.669': 0.013687, ((((Erinaceus: 0.277165, Podogymnura: 0.277165) '0.246-0.312': 0.299818, Sorex: 0.576983) '0.558-0.597': 0.035557, Talpa: 0.612541) '0.596-0.628': 0.006745, Solenodon: 0.619286) '0.604-0.635': 0.049818) '0.655-0.683': 0.057026, ((((Cynocephalus: 0.055888, Galeopterus: 0.055888) '0.043-0.072': 0.606905, ((Callithrix: 0.584318, Tarsius: 0.584318) '0.572-0.597': 0.031735, ((((Microcebus: 0.249924, Propithecus: 0.249924) '0.232-0.269': 0.007665, Lemur: 0.257589) '0.239-0.276': 0.174178, Daubentonia: 0.431767) '0.417-0.447': 0.029018, (Otolemur: 0.191179, Nycticebus: 0.191179) '0.165-0.219': 0.269605) '0.448-0.475': 0.155268) '0.609-0.624': 0.046740) '0.653-0.673': 0.003034, (Ptilocercus: 0.446257, Tupaia: 0.446257) '0.414-0.469': 0.219569) '0.656-0.676':

0.003307, ((Leporidae: 0.380274, Ochotona: 0.380274) '0.350-0.415': 0.269648, (((((((Abrocoma: 0.186099, ((Capromys: 0.124939, (Myocastor: 0.094771, Hoplomys: 0.094771) '0.086-0.104': 0.030169) '0.115-0.135': 0.048523, (Ctenomys: 0.152276, Octodontomys: 0.152276) '0.141-0.164': 0.021186) '0.163-0.184': 0.012637) '0.175-0.198': 0.091680, (Chinchilla: 0.215920, Dinomys: 0.215920) '0.200-0.231': 0.061859) '0.266-0.290': 0.022579, ((Agouti: 0.216843, (Cavia: 0.208064, Dasyprocta: 0.208064) '0.195-0.222': 0.008779) '0.204-0.230': 0.077132, Erethizon: 0.293975) '0.282-0.306': 0.006383) '0.289-0.312': 0.056920, (Heterocephalus: 0.322113, (Petromus: 0.181071, Thryonomys: 0.181071) '0.162-0.200': 0.141041) '0.310-0.334': 0.035166) '0.346-0.368': 0.017967, Hystrix: 0.375245) '0.363-0.387': 0.137981, (Ctenodactylus: 0.365450, Laonastes: 0.365450) '0.343-0.385': 0.147776) '0.500-0.525': 0.070965, (((Anomalurus: 0.451360, Pedetes: 0.451360) '0.431-0.468': 0.095496, (((((Cricetus: 0.200208, Mus: 0.200208) '0.185-0.217': 0.016638, Petromyscus: 0.216846) '0.202-0.233': 0.010926, Calomyscidae: 0.227772) '0.209-0.248': 0.144251, Spalax: 0.372024) '0.358-0.387': 0.081012, Jaculus: 0.453035) '0.439-0.466': 0.093821) '0.535-0.556': 0.005567, (Castor: 0.509792, (Geomyidae: 0.229782, Dipodomys: 0.229782) '0.200-0.260': 0.280010) '0.496-0.522': 0.042632) '0.541-0.562': 0.031767) '0.573-0.593': 0.002799, ((Aplodontia: 0.431487, Sciuridae: 0.431487) '0.402-0.455': 0.115890, Gliridae: 0.547377) '0.535-0.559': 0.039613) '0.576-0.596': 0.062933) '0.639-0.661': 0.019211) '0.659-0.678': 0.056997) '0.713-0.739': 0.046940) '0.758-0.789': 0.944141) '1.674-1.766': 0.324199, (Ornithorhynchus: 0.302551, Tachyglossus: 0.302551) '0.185-0.510': 1.738859) '1.960-2.099': 1.089116, ((Gallus: 0.756747, Taeniopygia: 0.756747) '0.662-0.865': 1.936147, Anolis: 2.692894) '2.558-2.849': 0.437632) '3.085-3.190': 0.606820, Xenopus: 3.737346) '3.631-3.804': 0.466153, Danio: 4.203499) '4.160-4.254': 0.000004;

B. Independent rates

(((((((((Didelphis: 0.162017, Monodelphis: 0.162017) '0.141-0.187': 0.039839, Glironia: 0.201855) '0.180-0.227': 0.014537, Caluromys: 0.216393) '0.193-0.242': 0.302095, (Dromiciops: 0.432836, ((Notoryctes: 0.415125, (((Isoodon: 0.084020, Echymipera: 0.084020) '0.070-0.099': 0.099201, Macrotis: 0.183221) '0.161-0.206': 0.222759, (Dasyurus: 0.197929, Myrmecobius: 0.197929) '0.174-0.226': 0.208051) '0.386-0.426': 0.009145) '0.396-0.435': 0.003841, ((((Acrobates: 0.275369, ((Petaurus: 0.231799, Pseudochirops: 0.231799) '0.214-0.252': 0.019117, Tarsipes: 0.250916) '0.234-0.271': 0.024453) '0.258-0.295': 0.033349, ((Macropus: 0.119242, Aepyprymnus: 0.119242) '0.101-0.137': 0.086585, Hypsiprymnodon: 0.205827) '0.185-0.227': 0.102891) '0.291-0.328': 0.006762, (Cercartetus: 0.291795, Trichosurus: 0.291795) '0.271-0.312': 0.023685) '0.297-0.334': 0.034272, (Phascolarctos: 0.213407, Vombatus: 0.213407) '0.183-0.242': 0.136345) '0.332-0.368': 0.069214) '0.401-0.438': 0.013869) '0.413-0.453': 0.085652) '0.495-0.545': 0.036556, (Caenolestes: 0.073916, Rhyncholestes: 0.073916) '0.061-0.088': 0.481128) '0.535-0.583': 1.096340, (((((Echinops: 0.569204, (Amblysomus: 0.078258, Chrysochloris: 0.078258) '0.066-0.093': 0.490946) '0.538-0.614': 0.048705, (Elephantulus: 0.429371, Rhynchocyon: 0.429371) '0.397-0.472': 0.188539) '0.591-0.662': 0.020452, (Heterohyrax: 0.031522, Procavia: 0.031522) '0.025-0.039': 0.606839) '0.610-0.680': 0.138100, (Dasypus: 0.448157, ((Choloepus: 0.151460, Bradypus: 0.151460) '0.131-0.169': 0.241655, (Cyclopes: 0.262222, Myrmecophaga: 0.262222) '0.231-0.299': 0.130893) '0.363-0.423': 0.055042) '0.425-0.481': 0.328304) '0.751-0.803': 0.005800, ((((((Moschus: 0.328840, Tragulus: 0.328840) '0.301-0.360': 0.179645, Pecari: 0.508485) '0.493-0.532': 0.144662, (((((Prionodon: 0.192961, Felis: 0.192961) '0.172-0.212': 0.032029, ((Fossa: 0.140347, Suricata: 0.140347) '0.120-0.160': 0.077403, Genetta: 0.217750) '0.199-0.235': 0.007241) '0.206-0.241': 0.046696, Nandinia: 0.271687) '0.253-0.285': 0.127315, ((Ailurus: 0.236771, (Procyon: 0.220304, Meles: 0.220304) '0.203-0.235': 0.016466) '0.222-0.251': 0.008400, Mephitis: 0.245170) '0.231-0.258': 0.153831) '0.378-0.424': 0.234206, (Manis_pentadactyla: 0.158387, Manis_tricuspis: 0.158387) '0.133-0.189': 0.474820) '0.609-0.653': 0.019940) '0.634-0.671': 0.000599, (((((Craseonycteris: 0.383034, Megaderma: 0.383034) '0.357-0.407': 0.030559, Rhinopoma: 0.413592) '0.390-0.435': 0.016372, (Hipposideros: 0.334253, Rhinolophus: 0.334253) '0.311-0.354': 0.095711) '0.408-0.450': 0.048368, Nyctimene: 0.478333) '0.452-0.502': 0.030584, (((((Miniopterus: 0.356171, Myotis: 0.356171) '0.331-0.381': 0.027737, Tadarida: 0.383909) '0.361-0.406': 0.011708, Natalus: 0.395617) '0.373-0.417': 0.023497, Myzopoda: 0.419114) '0.398-0.439': 0.009502, ((Emballonuridae: 0.391484, Nycteris: 0.391484) '0.366-0.414': 0.035388, ((((Furipterus: 0.310659, Noctilio: 0.310659) '0.283-0.335': 0.033486, (Pteronotus: 0.306410, Artibeus: 0.306410) '0.281-0.330': 0.037735) '0.322-0.365': 0.004613, Thyroptera: 0.348758) '0.326-0.370': 0.023165, Mystacina: 0.371924) '0.351-0.393': 0.054948) '0.407-0.446': 0.001745) '0.409-0.447': 0.080300) '0.485-0.532': 0.144829) '0.634-0.671': 0.014623, ((((Erinaceus: 0.331142, Podogymnura: 0.331142) '0.295-0.364': 0.242291, Sorex: 0.573433) '0.553-0.597': 0.033541, Talpa: 0.606973) '0.590-0.627': 0.006452, Solenodon: 0.613426) '0.597-0.634': 0.054943) '0.651-0.686': 0.061404, ((((Cynocephalus: 0.052745, Galeopterus: 0.052745) '0.042-0.065': 0.616187, ((Callithrix: 0.576025, Tarsius: 0.576025) '0.555-0.594': 0.033084, ((((Microcebus: 0.180315, Propithecus: 0.180315) '0.152-0.206': 0.008776,

Lemur: 0.189090) '0.161-0.216': 0.172658, Daubentonia: 0.361749) '0.335-0.401': 0.032663, (Otolemur: 0.154470, Nycticebus: 0.154470) '0.133-0.177': 0.239942) '0.372-0.430': 0.214697) '0.590-0.618': 0.059823) '0.654-0.685': 0.003497, (Ptilocercus: 0.438620, Tupaia: 0.438620) '0.401-0.492': 0.233809) '0.658-0.688': 0.003456, ((Leporidae: 0.407062, Ochotona: 0.407062) '0.364-0.440': 0.250289, (((((((Abrocoma: 0.238375, ((Capromys: 0.162607, (Myocastor: 0.128518, Hoplomys: 0.128518) '0.112-0.147': 0.034089) '0.146-0.181': 0.062992, (Ctenomys: 0.188012, Octodontomys: 0.188012) '0.169-0.205': 0.037586) '0.210-0.242': 0.012776) '0.223-0.255': 0.099639, (Chinchilla: 0.236871, Dinomys: 0.236871) '0.211-0.263': 0.101143) '0.320-0.356': 0.021949, ((Agouti: 0.250722, (Cavia: 0.241060, Dasyprocta: 0.241060) '0.222-0.267': 0.009662) '0.231-0.275': 0.099074, Erethizon: 0.349796) '0.331-0.369': 0.010167) '0.343-0.378': 0.057310, (Heterocephalus: 0.371402, (Petromus: 0.238332, Thryonomys: 0.238332) '0.215-0.263': 0.133069) '0.351-0.391': 0.045871) '0.401-0.434': 0.016486, Hystrix: 0.433759) '0.416-0.451': 0.111956, (Ctenodactylus: 0.391844, Laonastes: 0.391844) '0.359-0.423': 0.153871) '0.529-0.562': 0.056189, (((Anomalurus: 0.432250, Pedetes: 0.432250) '0.402-0.465': 0.130943, (((((Cricetus: 0.247204, Mus: 0.247204) '0.222-0.277': 0.023180, Petromyscus: 0.270384) '0.245-0.300': 0.010590, Calomyscidae: 0.280974) '0.253-0.312': 0.131249, Spalax: 0.412223) '0.389-0.434': 0.080913, Jaculus: 0.493136) '0.472-0.513': 0.070057) '0.550-0.577': 0.006254, (Castor: 0.515306, (Geomyidae: 0.273589, Dipodomys: 0.273589) '0.238-0.309': 0.241717) '0.496-0.536': 0.054141) '0.558-0.583': 0.032457) '0.591-0.614': 0.003501, ((Aplodontia: 0.407173, Sciuridae: 0.407173) '0.369-0.439': 0.153047, Gliridae: 0.560220) '0.541-0.576': 0.045185) '0.594-0.616': 0.051946) '0.643-0.672': 0.018534) '0.662-0.691': 0.053887) '0.711-0.750': 0.052489) '0.758-0.808': 0.869122) '1.544-1.723': 0.355493, (Ornithorhynchus: 0.221442, Tachyglossus: 0.221442) '0.182-0.263': 1.785435) '1.888-2.091': 1.126253, ((Gallus: 0.703248, Taeniopygia: 0.703248) '0.659-0.790': 1.917406, Anolis: 2.620654) '2.543-2.756': 0.512476) '3.087-3.196': 0.608338, Xenopus: 3.741468) '3.639-3.804': 0.475562, Danio: 4.217030) '4.163-4.256': 0.000004;

57-taxon trees

These analyses on Liu et al.'s [11] 200-gene "1st quintile" DNA alignments exclude taxa >10kg and/or >40 years maximum longevity, and employ our dR40 calibrations that are compatible with the 57 taxa (Additional file 2). All age values are averages over two separate MCMCtree runs for each of Liu et al.'s [11] three alignments. Compared to the original analyses [11] the midpoint for the primary placental interordinal diversification falls from 68.0 Ma to 63.2 Ma with the independent rates model, and from 94.7 Ma to 69.8 Ma with the autocorrelated rates model.

A. Autocorrelated rates

((danio_rer:4.009951167,gaste_acu:4.009951167)'3.901915-4.098745':0.153061,(xenop_tro:2.992037167,((anoli_car:2.235592,(pelod_sin:2.074507833,(melea_gal:0.630073667,gallu_gal:0.630073667)'0.560042-0.705630167':1.444434167)'1.974931667-2.179188333':0.161084)'2.136506667-2.340271667':0.274776667,(ornit_ana:1.961570167,((monod_dom:0.858418667,(macro_eug:0.721901333,sarco_har:0.721901333)'0.6876665-0.7722115':0.1365175)'0.824001333-0.909996667':0.786791833,(((dasyp_nov:0.642396667,cholo_hof:0.642396667)'0.6217875-0.662141667':0.167905,(proca_cap:0.695113,(echin_tel:0.6559355,(eleph_edw:0.6302145,chrys_asi:0.6302145)'0.61194-0.648303':0.025720833)'0.6381375-0.6734135':0.039177667)'0.677990833-0.712359333':0.115188667)'0.794834833-0.8264565':0.0159955,(((condy_cri:0.649287333,(erina_eur:0.578980167,sorex_ara:0.578980167)'0.5618925-0.5952485':0.070307333)'0.634486-0.663438833':0.0766,(((ptero_par:0.541694667,(eptes_fus:0.249276667,myoti_luc:0.249276667)'0.2343395-0.264716333':0.292418)'0.525374833-0.5571655':0.092581833,((megad_lyr:0.538334167,rhino_fer:0.538334167)'0.5197005-0.555794667':0.068168667,(eidol_hel:0.313999667,(ptero_ale:0.164221167,ptero_vam:0.164221167)'0.150771833-0.178123333':0.1497785)'0.296381833-0.331669667':0.292503)'0.591754-

0.620634667':0.027773667)'0.620136667-0.647733':0.076622,(manis_pen:0.678470833,(felis_cat:0.519982833,muste_put:0.519982833)'0.502780333-0.537512833':0.158487667)'0.663186-0.692583833':0.032428)'0.6972925-0.72352':0.014988667)'0.712554-0.738672833':0.0499495,(((tupai_bel:0.116492667,tupai_chi:0.116492667)'0.103172683-0.130656833':0.590893833,((ochot_pri:0.4496455,oryct_cun:0.4496455)'0.43451-0.464646333':0.2251665,(ictid_tri:0.6178115,((heter_gla:0.378906667,(cavia_por:0.319763,(chinc_lan:0.284177,octod_deg:0.284177)'0.273031-0.295333':0.035585833)'0.3083465-0.3312255':0.059144)'0.367387667-0.390659833':0.220072333,(dipod_ord:0.559421,(jacul_jac:0.486974167,((mus_mus:0.179309333,rattu_nor:0.179309333)'0.1704165-0.188362833':0.1136225,(perom_man:0.247835333,(micro_och:0.236000667,(crice_gri:0.166356833,mesoc_aur:0.166356833)'0.1581175-0.174942333':0.069643833)'0.2266905-0.245639833':0.011835)'0.238454-0.257550667':0.0450965)'0.2830045-0.303048':0.194042167)'0.477885167-0.4964675':0.072447)'0.551882-0.5675155':0.039558167)'0.592421833-0.606956333':0.018832333)'0.612064833-0.625620167':0.0570005)'0.666282-0.684486667':0.0325745)'0.697721667-0.718041333':0.009152833,(galeo_var:0.7031305,((daube_mad:0.558675833,(otole_gar:0.523555667,micro_mur:0.523555667)'0.5094505-0.536934':0.035119833)'0.5461475-0.5707385':0.100583833,(tarsi_syr:0.631945333,((saimi_bol:0.261519,calli_jac:0.261519)'0.246731667-0.276653167':0.1607955,(chlor_sab:0.167150833,macac_fas:0.167150833)'0.152353667-0.1823455':0.255163833)'0.406906833-0.437700667':0.209630833)'0.619986833-0.644151833':0.027314)'0.648272833-0.670448667':0.043871167)'0.692608167-0.714245':0.013409)'0.7065795-0.727328':0.059298)'0.763064667-0.788878167':0.050460333)'0.811315333-0.841789':0.818912833)'1.583878333-1.706136667':0.316359667)'1.884631667-2.039041667':0.548798333)'2.409576667-2.617465':0.481668833)'2.880176667-3.11035':1.170975)'4.1496-4.182095':0.008608333;

B. Independent rates

((danio_rer: 2.337138667,gaste_acu: 2.337138667) '2.210826667-2.462608333': 1.911249667,(xenop_tro: 3.367744333,((anoli_car: 2.090833833,(pelod_sin: 1.827375833,(melea_gal: 0.188194,gallu_gal: 0.188194) '0.171294333-0.2060885': 1.639181833) '1.739108333-1.917586667': 0.263458167) '2.007205-2.176666667': 0.4956765,(ornit_ana: 1.8877875,((monod_dom: 0.569510167,(macro_eug: 0.470279833,sarco_har: 0.470279833) '0.441405833-0.5009595': 0.099230333) '0.541951667-0.5996785': 0.976389,(((dasyp_nov: 0.562060333,cholo_hof: 0.562060333) '0.529321667-0.5896955': 0.186194667,(proca_cap: 0.640712333,(echin_tel: 0.602574667,(eleph_edw: 0.5767225,chrys_asi: 0.5767225) '0.558176333-0.597686167': 0.025852333) '0.58503-0.623663833': 0.038137667) '0.623321167-0.6613925': 0.107542667) '0.732549-0.7642195': 0.015781333,(((condy_cri: 0.576353333,(erina_eur: 0.516992333,sorex_ara: 0.516992333) '0.498698-0.5354105': 0.059361) '0.560484833-0.592026833': 0.065655167,(((ptero_par: 0.435838333,(eptes_fus: 0.1722355,myoti_luc: 0.1722355) '0.157904-0.186840167': 0.263602667) '0.4167115-0.454971667': 0.089710667,((megad_lyr: 0.441583833,rhino_fer: 0.441583833) '0.421265333-0.461663667': 0.0553595,(eidol_hel: 0.1419665,(ptero_ale: 0.056139833,ptero_vam: 0.056139833) '0.049872367-0.0628484': 0.085827) '0.129736-0.154871': 0.354976667) '0.4797575-0.514033833': 0.028605333) '0.509051167-0.541886': 0.097732,(manis_pen: 0.593892833,(felis_cat: 0.419861167,muste_put: 0.419861167) '0.398086333-0.4428665': 0.174031833) '0.578232167-0.608442667': 0.029388) '0.609522-0.636383': 0.018727667) '0.628384667-0.654977667': 0.068986667,(((tupai_bel: 0.053537333,tupai_chi: 0.053537333) '0.047432933-0.060089767': 0.605529833,((ochot_pri: 0.4099565,oryct_cun: 0.4099565) '0.387889167-0.431859833': 0.229617,(ictid_tri: 0.602961833,((heter_gla: 0.367709667,(cavia_por: 0.307600167,(chinc_lan: 0.2717485,octod_deg: 0.2717485) '0.2581035-0.284907667': 0.035851333) '0.294123833-0.321123': 0.0601095) '0.3539265-0.381933': 0.221362,(dipod_ord: 0.555639333,(jacul_jac: 0.491408,((mus_mus: 0.199733,rattu_nor: 0.199733) '0.188198-0.211354833': 0.128977667,(perom_man: 0.274097,(micro_och: 0.259868,(crice_gri: 0.178707833,mesoc_aur: 0.178707833) '0.16784-0.189664833': 0.081160167) '0.249429167-0.270370833': 0.014228833) '0.263653667-0.284523333': 0.054613833) '0.318220333-0.339025167': 0.162697167) '0.481361-0.500817833': 0.064231333) '0.547172333-0.562835': 0.033432333) '0.581537833-0.595900167': 0.01389) '0.594850167-0.610568833': 0.0366115) '0.630087333-0.648673667': 0.019493833) '0.648935333-0.668789667': 0.005308833,(galeo_var: 0.649667667,((daube_mad: 0.487389333,(otole_gar: 0.458905667,micro_mur: 0.458905667) '0.437971-0.478437833': 0.028483667) '0.466248167-0.506433833': 0.108057167,(tarsi_syr: 0.569089833,((saimi_bol: 0.1210405,calli_jac: 0.1210405) '0.110788833-0.131860167': 0.089967833,(chlor_sab: 0.047499833,macac_fas: 0.047499833) '0.04189815-0.053605567': 0.163508667)

'0.1970255-0.225762167': 0.358081333) '0.552792167-0.583611667': 0.026357) '0.580445833-0.608920833': 0.054220833) '0.637951167-0.660823667': 0.014708333) '0.654091833-0.674326167': 0.046619333) '0.698792833-0.7232255': 0.053040833) '0.748805-0.7794075': 0.7818625) '1.486786667-1.607661667': 0.341888667) '1.820058333-1.95854': 0.698722833) '2.511966667-2.665633333': 0.781234167) '3.294781667-3.461041667': 0.880644) '4.224871667-4.262103333': 0.008608333;

References

1. Phillips MJ. Geomolecular dating and the origin of placental mammals. Syst Biol. 2016;65(3):546-557.
2. Springer MS, Emerling CA, Meredith RW, Janečka JE, Eizirik E, Murphy WJ. Waking the undead: Implications of a soft explosive model for the timing of placental mammal diversification. Mol Phylogenet Evol. 2017;106:86-102.
3. Meredith RW, Janečka JE, Gatesy J, Ryder OA, Fisher CA, Teeling EC, Goodbla A, Eizirik E, Simão TLL, Stadler T et al. Impacts of the Cretaceous terrestrial revolution and KPg extinction on mammal diversification. Science. 2011;334(6055):521-524.
4. dos Reis M, Inoue J, Hasegawa M, Asher RJ, Donoghue PCJ, Yang Z. Phylogenomic datasets provide both precision and accuracy in estimating the timescale of placental mammal phylogeny. Proc Roy Soc B. 2012;279(1742):3491-3500.
5. Halliday TJD, Upchurch P, Goswami A. Eutherians experienced elevated evolutionary rates in the immediate aftermath of the Cretaceous - Palaeogene mass extinction. Proc Roy Soc B. 2016;283:20153026.
6. Bininda-Emonds ORP, Cardillo M, Jones KE, MacPhee RDE, Beck RMD, Grenyer R, Price SA, Vos RA, Gittleman JL, Purvis A. The delayed rise of present-day mammals. Nature. 2007;446(7135):507-512.
7. Murphy WJ, Pringle TH, Crider TA, Springer MS, Miller W. Using genomic data to unravel the root of the placental mammal phylogeny. Genome Res. 2007;17(4):413-421.
8. Springer MS, Murphy WJ, Eizirik E, O'Brien SJ. Placental mammal diversification and the Cretaceous–Tertiary boundary. Proc Natl Acad Sci USA. 2003;100(3):1056-1061.
9. Lartillot N, Phillips MJ, Ronquist F. A mixed relaxed clock model. Phil Trans Roy Soc B. 2016. 371:20150132.

10. Tarver JE, dos Reis M, Mirarab S, Moran RJ, Parker S, O'Reilly JE, King BL, O'Connell MJ, Asher RJ, Warnow T et al. The interrelationships of placental mammals and the limits of phylogenetic inference. Genome Biol Evol. 2016;8(2):330-344.
11. Liu L, Zhang J, Rheindt FE, Lei FM, Qu YH, Wang Y, Zhang Y, Sullivan C, Nie WH, Wang JH et al. Genomic evidence reveals a radiation of placental mammals uninterrupted by the KPg boundary. Proc Natl Acad Sci U S A. 2017;114(35):E7282-E7290.
12. Ronquist F, Lartillot N, Phillips MJ. Closing the gap between rocks and clocks using total-evidence dating. Phil Trans Roy Soc B. 2016;371:20150136.
13. Phillips MJ, Bennett TH, Lee MSY. Molecules, morphology and ecology indicate a recent, amphibious ancestry for echidnas. Proc Natl Acad Sci U S A. 2009;106(40):17089-17094.
14. Barba-Montoya J, dos Reis M, Schneider H, Donoghue PCJ, Yang Z. Constraining uncertainty in the timescale of angiosperm evolution and the veracity of a Cretaceous Terrestrial Revolution. New Phytol. 2018;218:819-834.
15. Meredith RW, Janečka JE, Gatesy J, Ryder OA, Fisher CA, Teeling EC, Goodbla A, Eizirik E, Simão TLL, Stadler T et al. Impacts of the Cretaceous terrestrial revolution and KPg extinction on mammal diversification. Science. 2011;334(6055):521-524.
16. Nguyen L-T, Schmidt HA, von Haeseler A, Minh BQ. IQ-TREE: a fast and effective stochastic algorithm for estimating maximum likelihood phylogenies. Mol Biol Evol. 2015;32:268-274
