# Supplementary material for: The soft explosive model of placental mammal evolution
Source: BMC Evol Biol. 2018 Jul 3;18:104. doi: 10.1186/s12862-018-1218-x (PMC6029115; doi:10.1186/s12862-018-1218-x)
Supplement: Supplementary file 3 — Fossil calibration schemes. Table S3. and Figure S4. (PDF 583 kb) [file 12862_2018_1218_MOESM3_ESM.pdf]

Additional File 3: Fossil calibration schemes

The soft explosive model of placental mammal evolution

Matthew J. Phillips*,[1] and Carmelo Fruciano[1]

[1]School of Earth, Environmental and Biological Sciences, Queensland University of Technology, Brisbane, Australia

*Corresponding author: E-mail: m9.phillips@qut.edu.au

Contents

Fossil Calibration schemes....1
  Table S3....1
  New calibrations....4
  Figure S4....8
References....11

Calibration schemes

Table S3. Soft-bound calibrations employed for the MCMCtree analyses of the 122-taxon, and 128-taxon empirical datasets. Calibrations among placental mammals are largely based on dos Reis et al. [1], hence the designations dR32 and dR40 (numbers indicating the number of calibrations). Several new calibrations, including some inspired by Springer *et al.* [2] are described below the table. Also note that the 122-taxon dR40 and 128-taxon analyses employ bounds as listed below, whereas the dR32 analyses, which are intended to be directly comparable with Phillips [3] use a previous chronostratigraphic timescale, with slightly different bounds. The Campanian maximum bound has moved from 84.2 Ma to 83.8 Ma, minimum Selandian bound from 61.5 Ma to 61.6 Ma, maximum Selandian bound from 61.7 Ma to 61.6 Ma, maximum Eocene bound from 55.6 Ma to 56.0 Ma, and the KPg boundary is set to 66.0 Ma (previously min 65.2 Ma, max 65.8 Ma).

Clade	Bounds (Ma)	Reference	122-taxon analyses dR32			128-taxon analysis
			dR32	dR40	dR40 Springer	
Placentalia	<131.5	[1]	✓	✓	✓	✓
Xenarthra	>55.6	[1]	✓			
Xenarthra	>47.8	New		✓	✓	✓
Folivora	15.97-41.3	[2]		✓	✓	✓
Afrotheria	>55.6	[1][a]	✓			
Afrotheria	>59.2	New		✓	✓	✓
Fereuungulata	>62.5	[1][b]	✓	✓	✓	✓
Carnivora	39.68-66	[1]	✓	✓	✓	✓
Feliformia	28.1-41.3	New		✓	✓	✓
Musteloidea	24.8-41.3	New		✓	✓	✓
Erinaceidae-Soricidae	>57.8	[3]	✓	✓		✓
Erinaceidae-Soricidae	61.6-83.8	[2]			✓	
Haplorhini	33.9-57.0	[1][c]	✓	✓		✓

Haplorhini	33.9-59.2	[2]			✓	
Primates	56-61.6	Min [1] Max [3]	✓	✓		✓
Primates	56-66	Min [1] Max [2]			✓	
Strepsirrhini	37.8-56	[1]	✓[d]	✓	✓	✓
Lorisiformes	38-56	[2]			✓	
Archonta	>61.6	[1]	✓	✓	✓	✓
Glires	>61.6	[1]	✓	✓	✓	✓
Lagomorpha	53.7-61.6	[2]			✓	
Rodentia	56-61.6	Min [1] Max [3]	✓	✓		✓
Rodentia	56-66	Min [1] Max [2]			✓	
Myomorpha-Hystricomorpha	52.5-59.2	[1]	✓	✓		✓
Myomorpha-Hystricomorpha	52.5-66.0[e]	Min [1] Max [2]			✓	
Myomorpha-Castorimorpha	40.2-56.0	[1]	✓	✓		✓
Myomorpha-Castorimorpha	40.2-66.0	Min [1] Max [2]			✓	
Caviomorpha-Phiomorpha	>35.8	New		✓	✓	✓
Chinchilloidea-Octodontoidea	>28.1	New		✓	✓	✓
Chiroptera	45.0-59.2	[3]	✓	✓		✓
Chiroptera	47.8-66.0	[2]			✓	
Rhinolophidae-Hipposideridae	33.9-53.0	New		✓	✓	✓
Emballonuroidea	47.8-59.2	[2]			✓	
Theria	124.0-171.2	[1]	✓	✓	✓	✓
Mammalia	162.9-191.1	[1]	✓			
Mammalia	162.9-208.5	New		✓	✓	✓
Suina-Cetruminatia	50.0-61.6	New				✓
Ruminantia	>33.9	New				✓
Osteichthyes	416-425.4	[4]	✓	✓	✓	✓

Tetrapoda	330.4-377.1	[4]	✓	✓	✓	✓
Amniota	312.3-347.4	[4]	✓	✓	✓	✓
Sauropsida	255.9-299.8	[4]	✓	✓	✓	✓
Neognathae	66-86.5	[4]	✓	✓	✓	✓
Caenolestidae	0.0-15.97	[4]	✓	✓	✓	✓
Australidelphia-Didelphimorphia	65.18-84.2	[4]	✓			
Australidelphia-Didelphimorphia	54.55-83.8	New		✓	✓	✓
Marsupialia	54.55-83.8	New		✓	✓	✓
Didelphimorphia	11.608-66	[4]	✓	✓	✓	✓
Didelphidae	11.608-28.5	[4]	✓	✓	✓	✓
Peramelidae	4.36-23.8	[4]	✓	✓	✓	✓
Peramelemorphia	4.36-54.65	[4]	✓	✓	✓	✓
Dasyuromorphia	15.97-54.65	[4]	✓	✓	✓	✓
Phalangeridae-Burramyidae	25-54.65	[4]	✓	✓	✓	✓
Petauridae-Pseudocheiridae	25.5-54.65	[4]	✓	✓	✓	✓
Macropodoidea	24.7-54.65	[4]	✓			
Macropodoidea	17.79-54.65	New		✓	✓	✓
Macropodiformes	24.7-54.65	New		✓	✓	✓
Vombatiformes	25.5-54.65	[4]				✓

[a]dos Reis et al. [1] and Phillips [3] used this bound for elephant/hyrax. With elephants excluded from the 122-taxon and 128-taxon analyses, instead of deleting the calibration we used the minimum bound only, for the next deepest included node (Afrotheria).

[b]dos Reis et al. [1] and Phillips [3] used this bound for horse/cat (Zoomata). With perissodactyls excluded from the 122-taxon and 128-taxon analyses, instead of deleting the calibration we used the minimum bound only, for the next deepest included node (Fereuungulata).

[c]dos Reis et al. [1] and Phillips [3] used this bound for Anthropoidea. With Catarrhini excluded from the 122-taxon and 128-taxon analyses, instead of deleting the calibration we used the minimum bound only for the next deepest included node, Haplorhini (tarsier/new world monkeys). The 57 Ma maximum bound covers the absence of crown haplorhines from relatively well sampled North American Clarkforkian and equivalent aged Eurasian primatomorph faunas

[5, 6], as well as the age of *Altiatlasius koulchii*, which is known from just a few isolated teeth and a jaw fragment, and is likely a stem primate [7], but has also been placed within haplorines [8].

[d]33.7-55.6 Ma following dos Reis et al. [1] and as used in Phillips [3].

[e]Springer et al. [2] used an alternative basal rodent relationship, Sciuromorpha-Hystricomorpha and employed a 66 Ma maximum bound. They also employed this bound for older (Rodentia) and younger (Myomorpha-Castorimorpha) rodent clades, and so the same bound may be inferred for Myomorpha-Hystricomorpha.

Calibration tree for the 57-taxon analyses of the Liu et al. [9] datasets

((danio_rer,gaste_acu),(xenop_tro,((anoli_car,(pelod_sin,(melea_gal,gallu_gal)))'2.559<2.998',(ornit_ana,((monod_dom,(macro_eug,sarco_har))'>0.5455<0.838',(((dasyp_nov,cholo_hof)'>0.478',(proca_cap,(echin_tel,(eleph_edw,chrys_asi)))'>0.592'),(((condy_cri,(erina_eur,sorex_ara)'>0.578'),(((ptero_par,(eptes_fus,myoti_luc)),((megad_lyr,rhino_fer),(eidol_hel,(ptero_ale,ptero_vam))))'>0.45<0.589',(manis_pen,(felis_cat,muste_put)'>0.3968<0.66'))'>0.625'),(((tupai_bel,tupai_chi),((ochot_pri,oryct_cun),(ictid_tri,((heter_gla,(cavia_por,(chinc_lan,octod_deg)'>0.281'))'>0.358',(dipod_ord,(jacul_jac,((mus_mus,rattu_nor),(perom_man,(micro_och,(crice_gri,mesoc_aur))))))'>0.402<0.56')'>0.525<0.589')'>0.56<0.616')'>0.616'),(galeo_var,((daube_mad,(otole_gar,micro_mur))'>0.378<0.56',(tarsi_syr,((saimi_bol,calli_jac),(chlor_sab,macac_fas)))'>0.337')'>0.56<0.616')'>0.616')))'<1.315')'>1.24<1.712')'>1.629<2.085')'3.123<3.474')'3.304<3.771')'>4.16<4.254';

New calibrations

Fourteen calibrations in Table S3 are listed as "New", and are described below. In many cases these are minor modifications of previously suggested calibration priors. Even more minor are changes that update several stratigraphic boundaries to the International Stratigraphic Chart (v.2016/10). The dates listed in Table S3 are for the 122-taxon dR40 and 128-taxon analyses.

1. Xenarthra (armadillo-sloth): minimum bound only, >47.8 Ma. This follows Springer et al. [2], and acknowledges the uncertainty in the dating of the Itaborai Fauna, which includes *Riostegotherium*. The placement of *Riostegotherium* with armadillos is also somewhat questionable, being based on osteoderms, which are also known from some extinct sloths. However, the date is further supported by *Astegotherium* likely being late Early Eocene [10]. *Astegotherium* osteoderms are also found in association with slightly younger jaw material, and so this reference taxon is preferable to *Riostegotherium*. For now we use Springer et al.'s [2] minimum bound, although radiometric dates (47.89 ± 1.21 Ma, [11]) overlying the Laguna Fría fossils could provide a more secure minimum age.

2. Afrotheria (elephant-tenrec): minimum bound only, >59.2 Ma. Here we follow Springer et al. [2] on the updated, late Selandian age of *Eritherium* [12, 13]. Although Cooper et al. [14] found that *Eritherium* may not be a stem proboscidean as was favoured by Gheerbrant [12], it is clearly a crown afrotherian.

3. Feliformia (cat-African palm civet): 28.1-41.3 Ma. This calibration is based on *Proailurus* and *Stenogale* for the minimum bound, and the absence of putative feliforms from well-sampled European and North American Bartonian carnivoran faunas for the maximum bound. Springer et al. [2] used both taxa as (stem felid) reference fossils for the shallower Felidae-Prionodontidae clade, however, there are no studies that place these fossil taxa within this clade with solid statistical support, while other studies (e.g. Spaulding and Flynn [15]) place them outside the feliform clade that includes felids, herpestids, hyaenas and viverrids, and hence also euplerids and *Prionodon*.

The thesis of Holliday [16] that Springer et al. [2] cite provides a highly unstable phylogeny. Both *Proailurus* and *Stenogale* are placed on the felid stem in an analysis that includes poorly sampled taxa (≥ 20% character completeness). However, in the more complete (≥ 50% character completeness) analysis, *Proailurs* falls well outside Felidae and into an implausibly shallow placement among euplerids. *Stenogale* was excluded for the more complete analysis, but its sister taxon in the 20% completeness analysis, *Herpestides*, also fell outside Felidae (among viverrids) in the more complete analysis. It is nevertheless generally agreed that *Proailurus* and *Stenogale* are feliforms.

4. Musteloidea (skunk-badger): 24.8-41.3 Ma. The minimum bound follows Springer et al. [2], and is based on *Promartes* (e.g. Finarelli [17]). In a combined DNA-morphology analysis Finarelli [17] nested *Promartes* implausibly, well within crown Mustelidae, a clade that is less than half the age of the fossil [18]. Some other studies also place oligobunines (which include *Promartes*) outside musteloids (e.g. [15, 19]). Thus, further investigation is warranted. However, we maintain the bound for now, given that another musteloid, *Amphictis* is known from similar aged deposits among the Quercy Phosphorites [20, 21].

We extend Springer et al.'s [2] maximum bound from 38 Ma to 41.3 Ma, to account for the perhaps unlikely event that *Mustelavus* (which is known from Priabonian sites) is a crown

musteloid. Our maximum bound acknowledges that putative musteloids are absent from well-sampled Bartonian and older carnivoran faunas.

5. Caviomorpha-Phiomorpha (Guinea pig-naked mole rat): minimum bound only, >35.8 Ma, based on *Cachiyacuy contamanensis* [22]. Phillips [23] tentatively suggested a minimum age of 40.94 Ma, in line with Antoine et al.'s [22] $^{40}Ar/^{39}Ar$ dating of biotite grains overlying the fossil-bearing sediments (43.44 ± 2.5 Ma), but cautioned against a hard bound, because of potential radiometric dating incongruence with biocorrelation for the fauna, and absence of rodents from well-sampled younger faunas. Further concerns with the radiometric dating of the Contamana fauna have been raised by Bond et al. [24], particularly around the distant association of the dated biotite grains to the fossiliferous strata, the high variance of the date estimates, and the possibility of reworking. At present we prefer to employ Antoine et al.'s [22] more cautions biocorrelation minimum (35.8 Ma).

6. Chinchilloidea-Octodontoidea (Chinchilla-degu): minimum bound only, >28.1 Ma, based on *Eoviscaccia frassinettii* from the Tiguiririca Fauna of Chile [25]. There is some uncertainty in the age of these fossils, because they were from a site on the opposite side of the Tinguiririca River (and without outcrop continuity) from sites with underlying 31.5 Ma dates. Nevertheless, mammal fossil bicorrelation indicates at least an Early Oligocene age (>28.1 Ma).

Springer et al. [2] used a younger *Eoviscaccia* fossil to place a 24.5 Ma minimum bound on the shallower Chinchillidae-Dinomyidae divergence. We caution against this, because these relationships based on morphology are highly confused. Dinomyids have typically been placed by morphology with cavioids, very distantly from chinchilloids (e.g. [26]), yet, on molecular evidence, the monotypic living *Dinomys branickii* diverges so close to chinchillids as to almost provide a basal polytomy with the deepest diverging chinchillids [27]. When Opazo [28] did not constrain the age of the *Dinomys*/Chinchillidae, their divergence was 19.1 ± 2.7 Ma, substantially younger than *Eoviscaccia* fossils. A possible explanation for the vastly differing apparent molecular and morphological closeness of *Dinomys* to crown chinchillids, is that *Eoviscaccia* diverged prior to the divergence of *Dinomys* from crown chinchillids, after which either *Dinomys* converged on more cavioid-like molars, or crown chinchillids and *Eoviscaccia* converged on higher crowned molars. Kramarz et al. [29] already show that such hypsodonty evolved independently among crown chinchillids. Moreover, the link between the living *Dinomys* and many of its supposed fossil dinomyid relatives (including *Scleromys* and neoepiblemids) has been

questioned (e.g. [25, 30]). This emphasizes the need to test the affinities of *Eoviscaccia* alongside both extant and extinct dinomyids as well as a boarder scope of caviomoprh taxa before it can be confidently used as a reference taxon for calibrating Chinchilloidea alone.

7. Rhinolophidae-Hipposideridae (horseshoe bats-leaf nosed bats): 33.9-53.0 Ma. This clade is difficult to calibrate, because they are dentally plesiomorphic [31] and apparently as a consequence, morphological phylogeny has typically grouped these rhinolophoids with other dentally plesiomorphic bats, the nycterids and emballonurids (e.g. [32]). Molecular studies instead reveal that rhinolophoids fall on the opposite side of the chiropteran root from nycterids and emballonurids (e.g. [33]. Recent morphological studies avoid these deeper level problems by including rhinolophoids without other crown bats (e.g. [31, 34, 35]), but this practice may also prevent identification of any true relationships of some fossils with modern bat taxa outside of the rhinolophid-hipposiderid clade. Some bats previously assigned to Hipposideridae (and Emballonuridae) have already been placed in a new family of uncertain affinities [36]. Another study [34] does favour stem hipposiderid placements for primarily dental *Hipposideros (Pseudorhinolophus)* and *Palaeophyllophora* specimens that they report from close to the Ypresian/Lutetian boundary (47.8 Ma). However, our reanalysis (Figure S4) shows that when only taxa with cranial or post-cranial material were included, both *Hipposideros (Pseudorhinolophus)* and *Palaeophyllophora* fell outside (or formed a basal polytomy with) the rhinolophid-hipposisderid clade. The proposed stem rhinolophid, *Protorhinolophus* also fell outside this clade, depending on taxon sampling (not shown). The oldest taxon that fell stably within crown Rhinolophidae-Hipposideridae was *Vaylatsia frequens*, which received 70% bootstrap support as sister to the extant rhinolophids, with all taxa included (not shown in Figure S4, due to lack of cranial and post-cranial sampling).

As our minimum bound for Rhinolophidae-Hipposideridae we employ *Vaylatsia frequens* [37], which is known from the Late Eocene (at least 33.9 Ma). This is preliminary, because other species of *Vaylatsia* are known from the Middle Eocene. Conversely, other authors consider *Vaylatsia* to be a hipposiderid, and when affinities cannot be confidently pinned to either basal crown lineage it is usually wise to also consider the possibility that the taxon may be excluded from both. Further non-dental material would also assist to confirm relationships among Eocene rhinolophoids. At present we use a very conservative 53 Ma maximum bound, acknowledging that putative members of Rhinolophidae-Hipposideridae are absent from relatively well-sampled middle-late Ypresian bat faunas.

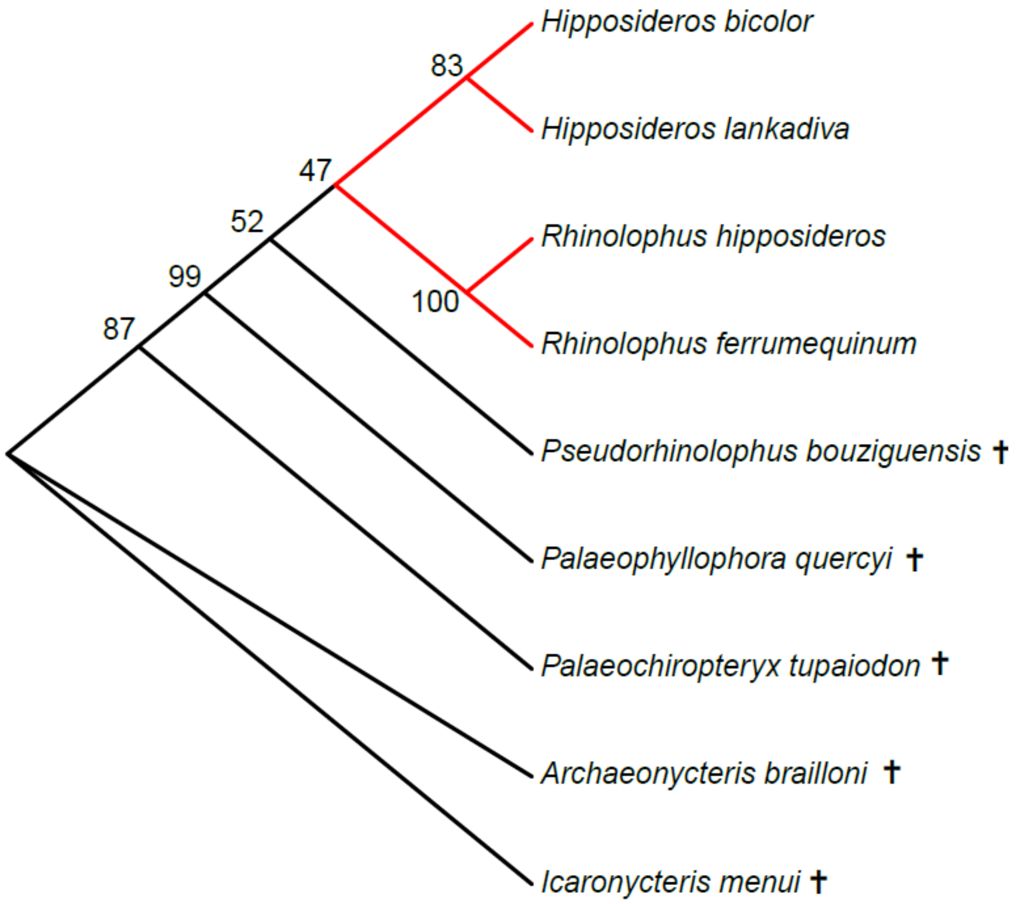

Figure S4. Rhinolophoid phylogeny from 500 maximum parsimony bootstrap pseudoreplicates (random addition heuristic searches with 50 replicates), with the crown branches indicated red. All characters from Ravel et al. [34] are included, but rhinolophoids scored primarily for dental characters and without cranial or postcranial characters are excluded here. The putative hipposiderid, *Pseudorhinolophus* and putative rhinolophid, *Palaeophyllophora* essentially form an unresolved polytomy with modern *Hipposideros* and *Rhinolophus*.

8. Mammalia: 162.9-208.5 Ma. Only the maximum bound is revised from dos Reis et al. [1], who used 191.1 Ma based on [38]. We agree with [1] that no good candidates for crown mammal fossils pre-date the Pliensbachian, however, there are very few Mammaliaformes sampled from this stage. Haramiyids have been linked to crown mammals (multituberculates), but Luo et al. [39] show that dental similarities are convergent, and Ramírez-Chaves et al. [40] show strong Bayesian posterior probability (1.00) for placing early haramiyids outside Mammalia based on non-molar characters. There is, however, only weak support for excluding the slightly older (Sinemurian) *Hadrocodium* from crown Mammalia [40]. The Hettangian and Rhaetian stages together provide relatively well sampled mammaliaform faunas for inferring the absence of putative crown mammals, which would allow for a 208.5 Ma maximum bound. Kuehneotheriids are generally thought to fall outside crown Mammalia. Although the cladistic analysis of Rougier

et al. [41] grouped *Kuehneotherium* close to monotremes and shuotheriids, reanalysis by Ramírez-Chaves et al. [40] without the apparently fastest evolving and most homoplastic molar characters restored *Kuehneotherium* to a stem mammal placement. Nevertheless, our maximum bound also covers kuehneotheriids.

9. Suina-Cetruminatia (pig-whale): 50.0-61.6 Ma. The minimum bound is based on *Himalayacetus*, the earliest stem cetacean [42]. A minimum age of ~52.5 Ma has been proposed (e.g. [4, 38]), based on nannoplankton biocorrelation [42]. However, concerns raised by van Tuinen and Hadly [43] about the validity of these biomarkers have yet to be addressed, and these authors noted a minimum (50.0 Ma) based on radiometric dates for strata that can be correlated with updated biomarkers. This revised minimum also more closely fits the ages of phylogenetically more robust cetruminants, such as *Indohyus* and *Pakicetus*. The maximum bound acknowledges the absence of crown artiodactyls from well-sampled early Thanetian and Selandian ungulate faunas across potential geographic origins for the clade in Eurasia and North America.

10. Ruminantia (mouse deer-musk dear): minimum bound only, >33.9 Ma, advocated by Springer et al. [2], based on Late Eocene Lophiomerycidae, an extinct group related to mouse deer [44].

11. Australidelphia-Didelphimorphia (kangaroo-opossum): 54.55-83.8 Ma. The minimum bound is based on the stem or crown australidelphian, *Djarthia murgonensis* [45, 46]. *Djarthia* is from the earliest Cenozoic Australian mammal fauna, dated at 54.6±0.05 Ma [47]. The maximum bound is retained from Springer et al. [2], although we caution against using their minimum bound (65.18 Ma) based on Horovitz et al.'s [48] inference for *Peradectes*. Beck [45] and Jansa et al. [49] note that Horovitz et al. [48] used traits from a mixed assemblage of individuals from different locations, which is problematic given Williamson et al.'s [50] finding that *Peradectes* is paraphyletic. Jansa et al. ([49], SI) also questioned the presence of traits that had been attributed to *Peradectes*, such as a tympanic wing of the alisphenoid, while Beck's [45] analysis favoured placing *Peradectes* outside crown Marsupialia (maximum parsimony) or unresolved relative to crown or stem affinities (Bayesian inference).

12. Marsupialia (kangaroo-shrew opossum): 54.55-83.8 Ma. The same minimum and maximum bounds, reference taxon and arguments are employed as for the Australidelphia-Didelphimorphia

clade above. Several proposed stem-paucituberculatans including *Bardalestes* and *Riolestes* [51] are possibilities for slightly older (Late Paleocene) crown marsupials. However, revised timing for these fossils to 50-53 Ma [52] and uncertainty over dental homologies also in the case of *Riolestes* [53] preclude using these taxa to define the minimum bound for Marsupialia.

13. Macropodoidea (kangaroo-bettong): 17.79-54.65 Ma. This calibration prior is discussed at length in [23], with the minimum based on the Early Miocene *Ganguroo bilamina*, but here we update the conservative biocorrelation-based age of 15.97 Ma (top of the Early Miocene) to 17.79 Ma, based on new radiometric dates for the Neville's Garden site at Riversleigh [54]. The maximum bound remains the maximum age of the Tingamarra Fauna from Murgon, which includes only far more "primitive" forms among marsupials [55]. The maximum is necessarily conservative, because of a long fossil record hiatus prior to several ~25Ma faunas that include putative macropodoids.

14. Macropodiformes (kangaroo-musky rat kangaroo): 24.7-54.65 Ma. The minimum bound is based on *Ngamaroo archeri* [56] from the Etadunna Formation, Zone D, which is radiometrically dated to 24.7-25.0 Ma [57]. Several species of *Nambaroo* are slightly older (Etadunna Formation Zone C), but are not as confidently placed within Macropodiformes [58] as is *Ngamaroo archeri*. The maximum bound remains as described above for Macropodoidea.

Note on Sirenia: Sirenia was excluded by Springer et al. [2] and from our analysis, based on size and longevity criteria (and the influence of these life history traits as evolutionary rate correlates). However, we do note in the main text a minimum age of 28.1 Ma for Sirenia, which is younger than the 41.3 Ma date that Springer et al. [2] based on *Eotheroides aegyptiacum*. The placement of *Eotheroides* is insufficiently well-supported for use as a strong prior. The phylogenetic analysis of Voss [59] placed *Eotheroides* as a stem sirenian, and although Springer et al. [60] favoured crown placement, majority-rule of their bootstrap analysis does not resolve stem or crown placement. More generally, the placement of early sirenians relative to the modern Dugongidae (dugongs) and Trichechidae (manatees) is potentially complicated by the plesiomorphic feeding and habitat ecology of early fully aquatic sirenians being more closely retained by dugongs than manatees [61].

We prefer to use the Early Oligocene (>28.1 Ma) *Priscosiren atlantica* [62] as the reference taxon for the crown Sirenia divergence. *Priscosiren* is generally agreed to be a stem member of

Dugongidae, and was the oldest taxon with majority-rule bootstrap support as a crown sirenian in Springer et al.'s [60] bootstrap analysis.

References

1. dos Reis M, Inoue J, Hasegawa M, Asher RJ, Donoghue PCJ, Yang Z. Phylogenomic datasets provide both precision and accuracy in estimating the timescale of placental mammal phylogeny. Proc Roy Soc B. 2012;279(1742):3491-3500.
2. Springer MS, Emerling CA, Meredith RW, Janečka JE, Eizirik E, Murphy WJ. Waking the undead: Implications of a soft explosive model for the timing of placental mammal diversification. Mol Phylogenet Evol. 2017;106:86-102.
3. Phillips MJ. Geomolecular dating and the origin of placental mammals. Syst Biol. 2016;65(3):546-557.
4. Meredith RW, Janečka JE, Gatesy J, Ryder OA, Fisher CA, Teeling EC, Goodbla A, Eizirik E, Simão TLL, Stadler T et al. Impacts of the Cretaceous terrestrial revolution and KPg extinction on mammal diversification. Science. 2011;334(6055):521-524.
5. Prufrock KA, Boyer DM, Silcox MT. The first major primate extinction: an evaluation of paleoecological dynamics of North American stem primates using a homology free measure of tooth shape. Am J Phys Anthropol. 2016;159:683-697.
6. Smith T, Quesnel F, De Plöeg G, De Franceschi D, Métais G, De Bast E, Folie A, Boura A, Claude J, Dupuis C et al. First Clarkforkian equivalent land mammal age in the latest Paleocene basal Sparnacian facies of Europe: fauna, flora, paleoenvironment and (bio)stratigraphy. 2014;PLoS ONE 9(1):e86229.
7. Herrera JP, Dávalos LM. Phylogeny and divergence times of lemurs inferred with recent and ancient fossils in the tree. Syst Biol. 2016;65(5):772-791.
8. Marivaux L, Ramdarshan A, Essid EM, Marzougui W, Ammar HK, Lebrun R, Marandat B, Merzeraud G, Tabuce R, Vianey-Liaud M. *Djebelemur*, a tiny pre-tooth-combed primate from the Eocene of Tunisia: a glimpse into the origin of crown strepsirhines. PLoS One. 2013;8(12):e80778.
9. Liu L, Zhang J, Rheindt FE, Lei FM, Qu YH, Wang Y, Zhang Y, Sullivan C, Nie WH, Wang JH et al. Genomic evidence reveals a radiation of placental mammals uninterrupted by the KPg boundary. Proc Natl Acad Sci U S A. 2017;114(35):E7282-E7290.
10. Tejedor MF, Goin FJ, Gelfo JN, López G, Bond M, Carlini AA, Scillato-Yané GJ, Woodburne MO, Chornogubsky L, Aragón E et al. New Early Eocene mammalian fauna from Western Patagonia, Argentina. Am Mus Novit. 2009;3638:1-43.

11. Gosses J. The Laguna del Hunco Formation: lacustrine and sub-aerial caldera fill, Chubut Province, Argentina. In: Geol Soc Am Ann Meeting Abstracts 2006. p. 502.
12. Gheerbrant E. Paleocene emergence of elephant relatives and the rapid radiation of African ungulates. Proc Natl Acad Sci USA. 2009;106(26):10717-10721.
13. Kocsis L, Gheerbrant E, Mouflih M, Cappetta H, Yans J, Amaghzaz M. Comprehensive stable isotope investigation of marine biogenic apatite from the late Cretaceous–Early Eocene phosphate series of Morocco. Palaeogeogr, Palaeoclimatol Palaeoecol. 2014;394:74-88.
14. Cooper LN, Seiffert ER, Clementz M, Madar SI, Bajpai S, Hussain ST, Thewissen JGM. Anthracobunids from the Middle Eocene of India and Pakistan Are Stem Perissodactyls. PLoS One. 2014;9(10):e109232.
15. Spaulding M, Flynn JJ. Phylogeny of the Carnivoramorpha: The impact of postcranial characters. J Syst Palaeontol. 2012;10(4):653-677.
16. Holliday JA. Phylogeny and character change in the feloid Carnivora. Unpublished Ph.D. Dissertation. The Florida State University. 2007.
17. Finarelli JA. A total evidence phylogeny of the Arctoidea (Carnivora: Mammalia): relationships among basal taxa. J Mamm Evol. 2008;15(4):231.
18. Koepfli K-P, Deere KA, Slater GJ, Begg C, Begg K, Grassman L, Lucherini M, Veron G, Wayne RK. Multigene phylogeny of the Mustelidae: Resolving relationships, tempo and biogeographic history of a mammalian adaptive radiation. BMC Biology. 2008;6(1):10.
19. Tomiya S. A new basal caniform (Mammalia: Carnivora) from the Middle Eocene of North America and remarks on the phylogeny of early carnivorans. PLoS One. 2011;6(9):e24146.
20. Wang X, McKenna MC, Dashzeveg D. *Amphicticeps* and *Amphicynodon* (Arctoidea, Carnivora) from Hsanda Gol Formation, central Mongolia and phylogeny of basal arctoids with comments on zoogeography. Am Mus Novit. 2005;3483:1-60.
21. Salesa MJ, Antón M, Peigné S, Morales J. Evidence of a false thumb in a fossil carnivore clarifies the evolution of pandas. Proc Natl Acad Sci U S A. 2006;103(2):379-382.
22. Antoine P-O, Marivaux L, Croft DA, Billet G, Ganerød M, Jaramillo C, Martin T, Orliac MJ, Tejada J, Altamirano AJ et al. Middle Eocene rodents from Peruvian Amazonia reveal the pattern and timing of caviomorph origins and biogeography. Proc Roy Soc B. 2012;279(1732):1319-1326.
23. Phillips MJ. Four mammal fossil calibrations: balancing competing palaeontological and molecular considerations. Palaeontol Electronica 2015;18(1):1-16.

24. Bond M, Tejedor MF, Campbell KE, Chornogubsky L, Novo N, Francisco G. Eocene primates of South America and the African origins of New World monkeys. Nature. 2015;520(7548):538-541.
25. Bertrand OC, Flynn JJ, Croft DA, Wyss AR. Two new taxa (Caviomorpha, Rodentia) from the Early Oligocene Tinguiririca Fauna (Chile). Am Mus Novit. 2012;3750:1-36.
26. Kramarz A. *Neoreomys* and *Scleromys* (Rodentia, Hystricognathi) from the Pinturas Formation, late Early Miocene of Patagonia, Argentina. Rev Mus Argent Cienc Nat Nueva Ser. 2006;8(1):53-62.
27. Alvarez A, Arevalo RLM, Verzi DH. Diversification patterns and size evolution in caviomorph rodents. Biol J Linn Soc. 2017;121(4):907-922.
28. Opazo JC. A molecular timescale for caviomorph rodents (Mammalia, Hystricognathi). Mol Phylogenet Evol. 2005;37(3):932-937.
29. Kramarz AG, Vucetich MG, Arnal M. A New Early Miocene chinchilloid hystricognath rodent; an approach to the understanding of the early chinchillid dental evolution. J Mamm Evol. 2013;20(3):249-261.
30. Vucetich MG, Verzi DH, Hartenberger JL. Review and analysis of the radiation of the South American Hystricognathi (Mammalia, Rodentia). CR Acad Sci IIA 1999, 329(10):763-769.
31. Ravel A, Marivaux L, Qi T, Wang YQ, Beard KC. New chiropterans from the middle Eocene of Shanghuang (Jiangsu Province, Coastal China): new insight into the dawn horseshoe bats (Rhinolophidae) in Asia. Zool Scr. 2014;43(1):1-23.
32. Gunnell GF, Simmons NB. Fossil evidence and the origin of bats. J Mamm Evol. 2005;12(1):209-246.
33. Teeling EC, Springer MS, Madsen O, Bates P, Brien SJ, Murphy WJ. A molecular phylogeny for bats illuminates biogeography and the fossil record. Science. 2005, 307:580-584.
34. Ravel A, Adaci M, Bensalah M, Charruault A-L, Essid EM, Ammar HK, Marzougui W, Mahboubi M, Mebrouk F, Merzeraud G et al. Origine et radiation initiale des chauves-souris modernes: nouvelles découvertes dans l'Éocène d'Afrique du Nord. Geodiversitas 2016;38(3):355-434.
35. Wilson LAB, Hand SJ, López-Aguirre C, Archer M, Black KH, Beck RMD, Armstrong KN, Wroe S. Cranial shape variation and phylogenetic relationships of extinct and extant Old World leaf-nosed bats. Alcheringa. 2016;40(4):509-524.

36. Maitre E, Sigé B, Escarguel G. A new family of bats in the Paleogene of Europe: systematics and implications for the origin of emballonurids and rhinolophoids. Neues Jahrbuch fur Geologie und Palaontologie-Abhandlungen. 2008;250(2):199.
37. Maitre E. Western European middle Eocene to early Oligocene Chiroptera: systematics, phylogeny and palaeoecology based on new material from the Quercy (France). Swiss J Palaeontol. 2014;133(2):141-242.
38. Benton MJ, Donoghue PCJ, Asher RJ. Calibrating and constraining molecular clocks. In: Hedges B, Kumar S, editors. The timetree of life. Oxford: Oxford University Press. 2009. p. 35-86.
39. Luo Z-X, Gatesy SM, Jenkins FA, Amaral WW, Shubin NH. Mandibular and dental characteristics of Late Triassic mammaliaform *Haramiyavia* and their ramifications for basal mammal evolution. Proc Natl Acad Sci USA 2015;112(51):E7101-E7109.
40. Ramírez-Chaves HE, Weisbecker V, Wroe S, Phillips MJ. Resolving the evolution of the mammalian middle ear using Bayesian inference. Front Zool. 2016;13(1):39.
41. Rougier GW, Apesteguia S, Gaetano LC. Highly specialized mammalian skulls from the Late Cretaceous of South America. Nature. 2011;479(7371):98-102.
42. Bajpai S, Gingerich PD. A new Eocene archaeocete (Mammalia, Cetacea) from India and the time of origin of whales. Proc Natl Acad Sci USA. 1998;95(26):15464-15468.
43. van Tuinen M, Hadly EA. Calibration and error in placental molecular clocks: a conservative approach using the cetartiodactyl fossil record. J Hered. 2004;95(3):200-208.
44. Janis CM, Theodor JM. Cranial and postcranial morphological data in ruminant phylogenetics. Zitteliana. 2014;B32:15-31.
45. Beck RMD. An ‘ameridelphian’ marsupial from the early Eocene of Australia supports a complex model of Southern Hemisphere marsupial biogeography. Naturwissenschaften 2012;99(9):715-729.
46. Beck RMD, Godthelp H, Weisbecker V, Archer M, Hand SJ. Australia's oldest marsupial fossils and their biogeographical implications. PLoS One 2008;3(3):e1858.
47. Godthelp H, Archer M, Cifelli R, Hand SJ, Gilkeson CF. Earliest known Australian Tertiary mammal fauna. Nature. 1992;356(6369):514-516.
48. Horovitz I, Martin T, Bloch J, Ladevèze S, Kurz C, Sánchez-Villagra MR. Cranial anatomy of the earliest marsupials and the origin of opossums. PLoS One. 2009;4(12):e8278.
49. Jansa SA, Barker FK, Voss RS. The early diversification history of didelphid marsupials: a window into South America's "splendid isolation". Evolution. 2014;68(3):684-695.

50. Williamson TE, Brusatte SL, Carr TD, Weil A, Standhardt BR. The phylogeny and evolution of Cretaceous–Palaeogene metatherians: cladistic analysis and description of new early Palaeocene specimens from the Nacimiento Formation, New Mexico. J Syst Palaeontol. 2012;10(4):625-651.
51. Goin FJ, Candela AM, Abello MA, Oliveira EV. Earliest South American paucituberculatans and their significance in the understanding of 'pseudodiprotodont' marsupial radiations. Zool J Linn Soc. 2009;155(4):867-884.
52. Woodburne MO, Goin FJ, Raigemborn MS, Heizler M, Gelfo JN, Oliveira EV. Revised timing of the South American early Paleogene land mammal ages. J South Am Earth Sci. 2014;54:109-119.
53. Beck RMD. Current understanding of the phylogeny of Metatheria: a review. In: Goin FJ, Forasiepi AM, editors. New World marsupials and their extinct relatives: 100 million years of evolution. Berlin: Springer. in press.
54. Woodhead J, Hand SJ, Archer M, Graham I, Sniderman K, Arena DA, Black KH, Godthelp H, Creaser P, Price E. Developing a radiometrically-dated chronologic sequence for Neogene biotic change in Australia, from the Riversleigh World Heritage Area of Queensland. Gondwana Research. 2016;29(1):153-167.
55. Black KH, Archer M, Hand SJ, Godthelp H. The Rise of Australian Marsupials: A synopsis of biostratigraphic, phylogenetic, palaeoecologic and palaeobiogeographic understanding. In: Talent JA, editor. Earth and life: global biodiversity, extinction intervals and biogeographic perturbations through time. Dordrecht: Springer Netherlands. 2012. p. 983-1078.
56. Kear BP, Pledge NS. A new fossil kangaroo from the Oligocene-Miocene Etadunna Formation of Ngama Quarry, Lake Palankarinna, South Australia. Aust J Zool. 2008;55(6):331-339.
57. Woodburne MO, Macfadden BJ, Case JA, Springer MS, Pledge NS, Power JD, Woodburne JM, Springer KB. Land mammal biostratigraphy and magnetostratigraphy of the Etadunna Formation (late Oligocene) of South Australia. J Vertebr Paleontol. 1994;13(4):483-515.
58. Butler K, Travouillon KJ, Price GJ, Archer M, Hand SJ. *Cookeroo*, a new genus of fossil kangaroo (Marsupialia, Macropodidae) from the Oligo-Miocene of Riversleigh, northwestern Queensland, Australia. J Vertebr Paleontol. 2016;36(3):e1083029.
59. Voss M. On the invalidity of *Halitherium schinzii* Kaup, 1838 (Mammalia, Sirenia), with comments on systematic consequences. Zoosyst Evol. 2014;90:87-93.

60. Springer MS, Signore AV, Paijmans JLA, Vélez-Juarbe J, Domning DP, Bauer CE, He K, Crerar L, Campos PF, Murphy WJ et al. Interordinal gene capture, the phylogenetic position of Steller's sea cow based on molecular and morphological data, and the macroevolutionary history of Sirenia. Mol Phylogenet Evol. 2015;91:178-193.
61. Domning D. A phylogenetic analysis of the Sirenia. Proc S Diego Soc Nat Hist. 1994;29:177-189.
62. Vélez-Juarbe J, Domning DP. Fossil Sirenia of the West Atlantic and Caribbean region: X. *Priscosiren atlantica*, gen. et sp. nov. J Vertebr Paleontol. 2014;34(4):951-964.
