# Supplementary material for: The soft explosive model of placental mammal evolution
Source: BMC Evol Biol. 2018 Jul 3;18:104. doi: 10.1186/s12862-018-1218-x (PMC6029115; doi:10.1186/s12862-018-1218-x)
Supplement: Supplementary file 4 — GC3 conservation and estimating maximum longevity. Figure S5. (PDF 251 kb) [file 12862_2018_1218_MOESM4_ESM.pdf]

Additional File 4: GC3 conservation and estimating maximum longevity

The soft explosive model of placental mammal evolution

Matthew J. Phillips*[,1] and Carmelo Fruciano[1]

[1]School of Earth, Environmental and Biological Sciences, Queensland University of Technology, Brisbane, Australia

*Corresponding author: E-mail: m9.phillips@qut.edu.au

Contents

GC3 conservation and estimating maximum longevity 1

Figure S5 1

References 4

GC3 conservation and estimating maximum longevity

Romiguier et al. [1] examined protein coding GC content at 3rd positions (GC3) among mammals, and presented a time-correlated index of GC3 conservation,

$$\gamma = -t/\log(\tau) \qquad (1)$$

where t is time since divergence and τ is Kendall's correlation coefficient for GC3 conservation among genes, between species. We recalculated γ for each of Romiguier et al.'s [1] taxon pairs, using their GC3 conservation coefficients (τ), but now with divergence estimates from Phillips [2]. We confirm the strong correlation between γ and maximum longevity (R^2=0.91), which can be expressed as equation (2).

$$\text{maximum longevity} = 0.0683\gamma - 10.243 \qquad (2)$$

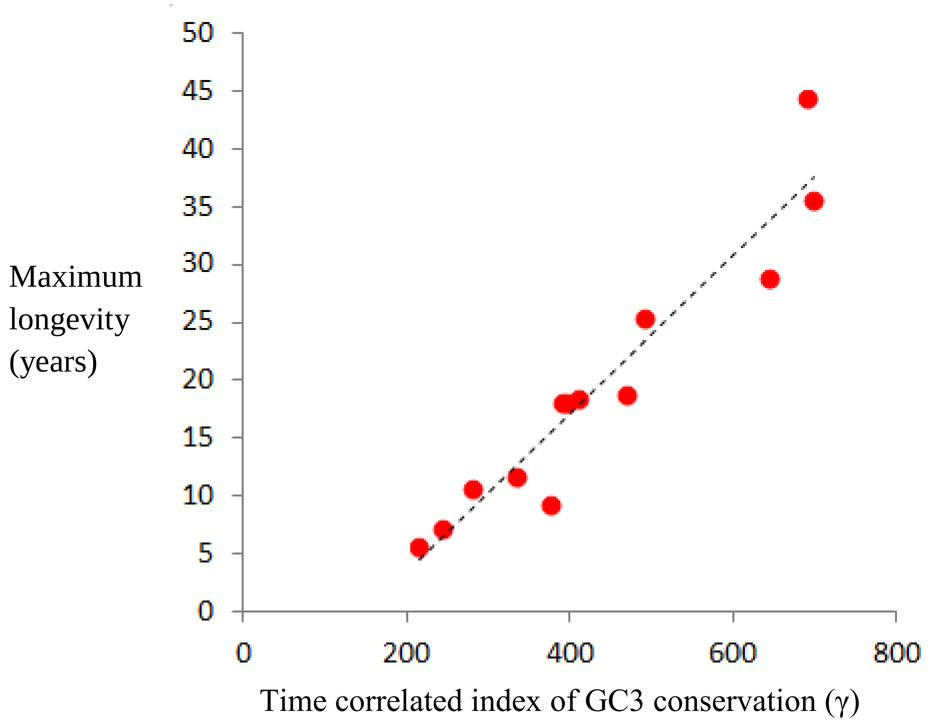

Figure S5. Relationship between maximum longevity and GC3 conservation (γ) for the same 13 placental mammal taxon pairs employed by Romiguier et al. [1], and with γ calculated in the same way, with γ = -t/log(τ). The values for τ (Kendall's correlation coefficient for GC3 conservation among genes, between species) are taken from Romiguier et al. [1], and the values for t (time since divergence between pairs) are taken from Phillips ([2], fig. 5). Maximum longevity is the average of family means for the taxon pairs (taken from [1]).

Romiguier et al. [1] focused on the path of GC3 conservation across the placental root from catarrhine primates to paenungulate afrotherians. They found the GC3 conservation coefficient (τ) for these two stem lineages back to the placental root to be 0.84, giving $\log(\tau) = -0.07572$. We refer to this as $\log(\tau)_{\text{Cat-to-Paen.}}$

The $\log(\tau)$ values are additive, and so can be considered as the sum of $\log(\tau)$ values for the catarrhine stem, the paenungulate stem and the basal placental lineages from the haplorhine primate and afrotherian nodes back to the placental root. Thus,

$$\log(\tau)_{\text{Cat-to-Paen}} = \log(\tau)_{\text{BasalPlacentals}} + \log(\tau)_{\text{CatStem}} + \log(\tau)_{\text{PaenStem}} \quad (3)$$

It is possible to obtain $\log(\tau)$ for both the catarrhine stem and the paenungulate stem by first using the correlation equation (2) to estimate γ from maximum longevity, for which we used the AnAge database (accessed 9 February 2017, [3]) averages for the two catarrhine clades (32.96 years) and for the elephant and hyrax clades (39.725 years). This gives values of γ of 632.55 for the catarrhines and 731.60 for the paenungulates.

Next, inverting the GC3 conservation equation (1) to $\log(\tau) = -t/\gamma$ we estimated $\log(\tau)$ for the catarrhine stem, $\log(\tau)_{\text{CatStem}} = -0.02490$ and for the paenungulate stem, $\log(\tau)_{\text{PaenStem}} = -0.00653$. The Divergence path-times (t) were taken from Phillips ([2], fig. 5) for catarrhines from the haplorhine primate origin (from 56.5 to 25.0 Ma) and similarly for paenungulates from the afrotherian origin (from 64.86 to 55.3 Ma). Note that t is averaged over the divergence of two lineages, and so contributions from individual lineages require values to be halved. For example, t for 56.5 to 25.0 Ma is (56.5-25.0)/2 Ma.

The remaining contribution to GC3 conservation among the basal placentals, along the lineages from the placental root to both the haplorhine primate and afrotherian crown origins, given equation (3) and the $\log(\tau)$ values calculated above, is:

$$\log(\tau)_{\text{BasalPlacentals}} = \log(\tau)_{\text{Cat-to-Paen}} - (\log(\tau)_{\text{CatStem}} + \log(\tau)_{\text{PaenStem}}) = -0.07572 - (-0.02490 - 0.00653) = -0.04429.$$

For the soft explosive model, using our mean 77 Ma estimate for the placental origin from the dR32/dR40 analyses, the average time since divergence (t) back to the placental origin for catarrhines and afrotherians is

((77-56.5) + (77-64.86))/2 = 16.32 Ma.

Substituting these $\log(\tau)_{BasalPlacentals}$ and t values into equation (1) gives the time-correlated index of GC3 conservation, γ (368.51). In turn, substituting γ into the correlation equation (2) gives the estimate for maximum longevity of 14.9 years, on average over the basal placental lineages. Calculating γ instead with the lower and upper bounds of the 95% CI for the regression equation (2) gives a confidence interval of 7.9 – 21.9 years for this maximum longevity estimate. As discussed in the main text, this result is consistent with expectations from longevity data for small mammals within the size range known for Campanian and earlier placentals (<250 g), and in turn, lends confidence to Romiguier et al's [1] GC3 conservation method for inferring maximum longevity.

Romiguier et al. [1] also inferred lineage specific maximum longevity for internal branches from regression of longevity against d*N*/d*S* for external branches. The average maximum longevity estimate along the 10 branches from the catarrhine to paenungulate stems was 29.0 years, which is older than both our GC3 conservation estimate and the expectations from the fossil record, given Cretaceous mammal sizes (see main text). However, d*N*/d*S* may be expected to be less reliable than GC3 conservation, so long as the latter is based on a relatively well-dated tree. The problems for the d*N*/d*S* method in the present context are at least three-fold.

(1) The non-synonymous rate is highly subject to selection. It has been hypothesised that the primary diversification of placentals coincided with major adaptive shifts (associated with the KPg event or not). Positive or relaxed selection would inflate d*N*/d*S*, but would have little influence on GC3 conservation.

(2) Forcing a fixed tree and positive branch lengths in the presence of alternative phylogenetic signals that are "off the tree" (in the sense of Penny et al. [4]) can bias branch-length and therefore rate estimates, and might be expected to be relatively greater with less signal, such as for d*N* relative to d*S*. Any such branch-length

estimation bias will be inflated for Romiguier et al. [1] because the method of Jobson et al. [5] that they use employs the shorter amino acid branch-lengths in both the numerator (for d*N*) and in the denominator (subtracted from nucleotide branch-length, giving d*S*). This may explain why Romiguier et al.'s [1] highest d*N*/d*S* values for lineages connecting Primates and Afrotheria were inferred for the two shortest internal branches along this path, the archontan and atlantogenatan stems.

(3) Substitution models are always misspecified to some extent for empirical data. However, it is well established that the influences of saturation, and non-stationarity (which both tend to inflate d*N*/d*S*) increase with phylogenetic depth (e.g. [6]).

The bias these three points describe may also explain the unexpected finding of Romiguier et al. [1] that average internal and external branch d*N*/d*S* values were almost identical, despite the latter including several very large, long-lived mammals, such as apes, dolphin and elephant.

References

1. Romiguier J, Ranwez V, Douzery EJP, Galtier N: Genomic evidence for large, long-lived ancestors to placental mammals. Mol Biol Evol. 2013;30(1):5-13.
2. Phillips MJ: Geomolecular dating and the origin of placental mammals. Syst Biol. 2016;65(3):546-557.
3. de Magalhaes JP, Costa J. A database of vertebrate longevity records and their relation to other life-history traits. J Evol Biol. 2009;22(8):1770-1774.
4. Penny D, White WT, Hendy MD, Phillips MJ: A bias in ML estimates of branch lengths in the presence of multiple signals. Mol Biol Evol. 2008;25(2):239-242.
5. Jobson RW, Nabholz B, Galtier N: An evolutionary genome scan for longevity-related natural selection in mammals. Mol Biol Evol. 2010;27(4):840-847.
6. Phillips MJ: Branch-length estimation bias misleads molecular dating for a vertebrate mitochondrial phylogeny. Gene. 2009;441(1–2):132-140.
