# Supplementary material for: The soft explosive model of placental mammal evolution
Source: BMC Evol Biol. 2018 Jul 3;18:104. doi: 10.1186/s12862-018-1218-x (PMC6029115; doi:10.1186/s12862-018-1218-x)
Supplement: Supplementary file 5 — Table S4. Fossil record species richness for Eutheria and Mammalia from Albian through to Lutetian. (PDF 90 kb) [file 12862_2018_1218_MOESM5_ESM.pdf]

Additional File 5: Fossil record species richness for Eutheria and Mammalia from Albian through to Lutetian

The soft explosive model of placental mammal evolution

Matthew J. Phillips*[,1] and Carmelo Fruciano[1]

[1]School of Earth, Environmental and Biological Sciences, Queensland University of Technology, Brisbane, Australia
*Corresponding author: E-mail: m9.phillips@qut.edu.au

Contents

Table S4..1

Table S4. Fossil record species richness for Eutheria and Mammalia from Albian through to Lutetian. From The Paleobiology Database, accessed 29 March, 2017.

Stages	Interval (Ma)	New eutherian species	New mammal species	Standing eutherian species richness	Eutherian species diversificat-ion rate[a]	Eutheria/ Mammalia new species appearances
Albian-Cenomanian	113.0-93.9	11	91	13	0.144[b]	0.121
Turonian-Santonian	93.9-83.6	22	64	25	0.164	0.344
Campanian	83.6-72.1	32	135	38	0.111	0.237
Maastrichtian	72.1-66.0	30	94	43	0.129	0.319
Paleocene	66.0-56.0	778	925	940	1.809	0.841
Ypresian	56.0-47.8	1118	1215	1258	0.144	0.920
Lutetian	47.8-41.2	914	996	1156	0.110	0.918

[a]New appearances per Ma, per standing eutherian diversity in the *previous* time bin.

[b]Eutherian standing species richness from the previous time bin, Aptian-Barremian is four.
